# Transcriptome Profiling of Khat (*Catha edulis*) and *Ephedra sinica* Reveals Gene Candidates Potentially Involved in Amphetamine-Type Alkaloid Biosynthesis

**DOI:** 10.1371/journal.pone.0119701

**Published:** 2015-03-25

**Authors:** Ryan A. Groves, Jillian M. Hagel, Ye Zhang, Korey Kilpatrick, Asaf Levy, Frédéric Marsolais, Efraim Lewinsohn, Christoph W. Sensen, Peter J. Facchini

**Affiliations:** 1 Department of Biological Sciences, University of Calgary, Calgary, Alberta, Canada; 2 Department of Biochemistry and Molecular Biology, University of Calgary, Calgary, Alberta, Canada; 3 Department of Biology, Western University, London, Ontario, Canada; 4 Southern Crop Protection and Food Research Centre, Agriculture and Agri-Food, London, Ontario, Canada; 5 The Robert H. Smith Institute of Plant Science and Genetics in Agriculture, The Hebrew University of Jerusalem, Rehovot, 76100, Israel; 6 Department of Aromatic, Medicinal and Spice Crops, Newe Ya’ar Research Center, Agricultural Research Organization, P.O. Box 1021, Ramat Yishay, 30095, Israel; Cankiri Karatekin University, TURKEY

## Abstract

Amphetamine analogues are produced by plants in the genus *Ephedra* and by khat (*Catha edulis*), and include the widely used decongestants and appetite suppressants (1*S*,2*S*)-pseudoephedrine and (1*R*,2*S*)-ephedrine. The production of these metabolites, which derive from L-phenylalanine, involves a multi-step pathway partially mapped out at the biochemical level using knowledge of benzoic acid metabolism established in other plants, and direct evidence using khat and *Ephedra* species as model systems. Despite the commercial importance of amphetamine-type alkaloids, only a single step in their biosynthesis has been elucidated at the molecular level. We have employed Illumina next-generation sequencing technology, paired with Trinity and Velvet-Oases assembly platforms, to establish data-mining frameworks for *Ephedra sinica* and khat plants. Sequence libraries representing a combined 200,000 unigenes were subjected to an annotation pipeline involving direct searches against public databases. Annotations included the assignment of Gene Ontology (GO) terms used to allocate unigenes to functional categories. As part of our functional genomics program aimed at novel gene discovery, the databases were mined for enzyme candidates putatively involved in alkaloid biosynthesis. Queries used for mining included enzymes with established roles in benzoic acid metabolism, as well as enzymes catalyzing reactions similar to those predicted for amphetamine alkaloid metabolism. Gene candidates were evaluated based on phylogenetic relationships, FPKM-based expression data, and mechanistic considerations. Establishment of expansive sequence resources is a critical step toward pathway characterization, a goal with both academic and industrial implications.

## Introduction

**Fig 1:**
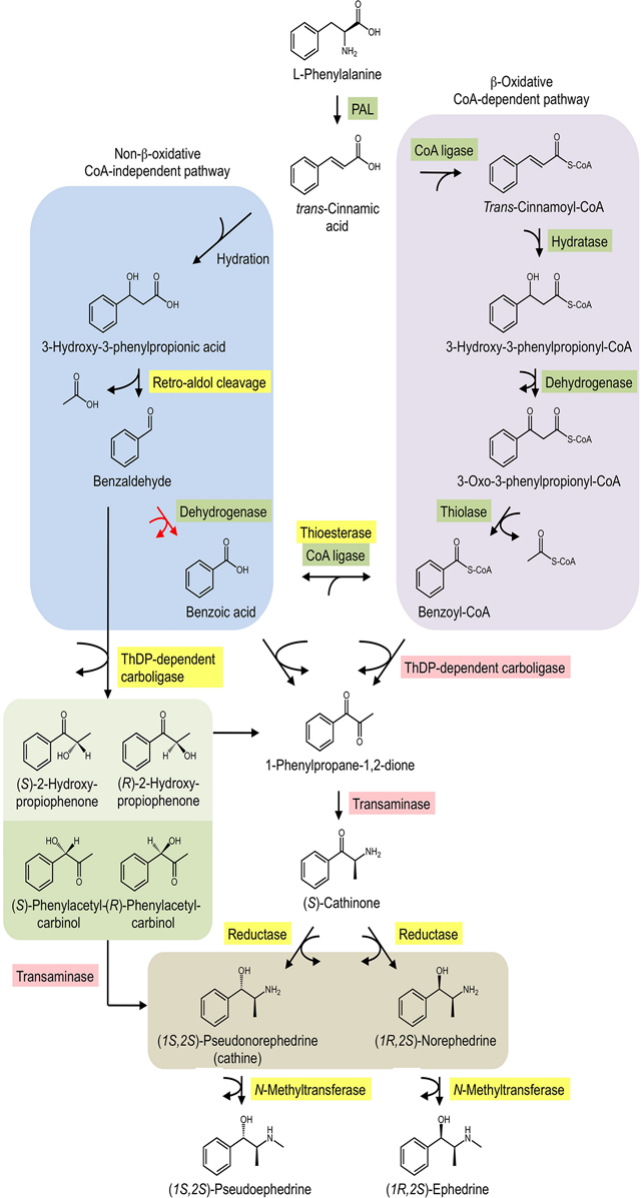
Proposed biosynthetic routes leading from L-phenylalanine to amphetamine-type alkaloids in khat and *Ephedra sinica*. A CoA-independent, non-β-oxidative pathway of L-phenylalanine side chain-shortening is shown in blue, whereas a CoA-dependent, β-oxidative route is shown in purple. Benzaldehyde, benzoic acid and/or benzoyl-CoA undergo condensation with pyruvate, a reaction putatively catalyzed by a ThDP-dependent carboligase. 1-Phenylpropane-1,2-dione undergoes transamination to yield (*S*)-cathinone, which is reduced to cathine and (1*R*,2*S*)-norephedrine. *N*-Methylation is restricted to *Ephedra* spp. and does not occur in khat. Activity has been detected for enzymes highlighted in yellow, and corresponding genes are available for enzymes highlighted in green. Enzymes highlighted in red have not been isolated, although database mining revealed numerous potential candidates (Tables 2 and 3). Abbreviations: CoA, Coenzyme A; NAD(H), nicotinamide adenine dinucleotide; NADP(H), nicotinamide adenine dinucleotide phosphate. PAL, phenylalanine ammonia lyase; ThDP, thiamine diphosphate.

Plants produce a wide variety of specialized nitrogenous metabolites, including a large number of pharmacologically important alkaloids. Ephedrine alkaloids, also termed phenylpropylamino alkaloids or substituted amphetamines, are a diverse compound class featuring a phenethylamine backbone with a methyl group at the α-position relative to the nitrogen [1]. Myriad functional group substitutions have yielded a series of synthetic drugs with diverse pharmacological properties as stimulants, empathogens, and hallucinogens [2]. Although most analogues are synthetic, certain plants have evolved a capacity for their biosynthesis. Two prominent examples include khat (*Catha edulis*) and *Ephedra sinica*, each of which have been cultivated for centuries owing to their mild stimulant and medicinal properties. The chewing of khat leaves as a social activity dates back at least a thousand years [3], a practice that might even predate the use of coffee [4]. Khat chewing remains an important tradition in parts of East Africa and the Middle East, although possession of khat is illegal in many Western countries. (*S*)-Cathinone (Fig. 1) is the principle active neurostimulant in khat, although other alkaloids such as (1*S*,2*S*)-pseudonorephedrine (cathine) and (1*R*,2*S*)-norephedrine also occur. Alkaloid biosynthesis in *E*. *sinica* extends beyond that of khat to include *N*-methylated enantiomers (1*S*,2*S*)-pseudoephedrine and (1*R*,2*S*)-ephedrine (Fig. 1). *N*,*N*-Dimethylated versions of these alkaloids also occur, although in lesser quantities [5]. *E*. *sinica*, known as Ma Huang in Chinese medicine, also has a long history of traditional use, particularly in the treatment of asthma, coughs, congestion, headaches and edema [6]. Today, (1*S*,2*S*)-pseudoephedrine is a common ingredient in cold and allergy formulations, whereas (1*R*,2*S*)-ephedrine is marketed as decongestant, diet aid and sports-enhancing drug.

Despite their occurrence in plants, the modern manufacture of (1*S*,2*S*)-pseudoephedrine and (1*R*,2*S*)-ephedrine relies predominantly on a process involving fermentation coupled with synthetic chemistry [1]. The fact that industrial production of these compounds is at least partially completed through fermentation raises the intriguing possibility of employing synthetic biology for the production of these alkaloids. Such an endeavor requires thorough knowledge of the enzyme catalysts responsible for alkaloid biosynthesis in plants. The first step in the pathway is catalyzed by L-phenylalanine ammonia lyase (PAL), a well-characterized enzyme in many species including *E*. *sinica* [7] (Fig. 1). Recent work in the field of floral scent biosynthesis has largely elucidated benzoic acid metabolism, a process occurring either in the peroxisomes as part of the core β-oxidative CoA-dependent pathway, or in the cytosol as part of the non-β-oxidative, CoA-independent pathway [8]. Alternatively, benzaldehyde may be formed via phenylpyruvate, a transamination product of L-phenylalanine (Phe). This route occurs in lactic acid bacteria [9] but has not been confirmed in plants. Whereas Phe serves as the initial precursor for the aromatic C_6_-C_1_ component of ephedrine alkaloids, the C_2_-C_3_ portion derives from pyruvate through a carboligation mechanism, likely catalyzed by a ThDP-dependent enzyme (Fig. 1). It was previously suggested that either benzoic acid or benzoyl-CoA serves as the C_6_-C_1_ carboligation co-substrate [10], although recent evidence supports the involvement of benzaldehyde [11], at least in *Ephedra* species. Stem extracts of *E*. *sinica* and *E*. *foeminea* catalyzed the conversion of benzaldehyde and pyruvate to five distinct carboligation products, including 1-phenylpropane-1,2-dione. Although the gene encoding the benzaldehyde carboligase (BCL) was not identified, the *in vitro* turnover of diverse C_6_-C_3_ backbone structures prompted a reconsideration of possible route(s) leading to alkaloid formation *in planta*. It has long been assumed that 1-phenylpropane-1,2-dione, a naturally occurring metabolite of both khat [12,13] and *E*. *sinica* [5], is a key intermediate. Transamination of this diketone yields (*S*)-cathinone, which undergoes stereospecific reduction to either cathine or (1*R*,2*S*)-norephedrine. *N*-Methylation of these diastereomers completes the pathway in *E*. *sinica*. An alternative route circumventing 1-phenylpropane-1,2-dione is possible, although the natural occurrence of alternative transaminase substrates (Fig. 1) has not been confirmed.

Despite the commercial importance of amphetamine analogues and the potential for synthetic biology applications [1], PAL remains the only step characterized at the molecular level in plants accumulating these compounds [7]. We recently reported a Sanger-based, expressed sequence tag (EST) library from khat containing 3,293 unigenes [14]. Here we report a dramatic expansion of our functional genomics platform to include over 200,000 unigenes, derived from Illumina-based, next-generation sequencing (NGS, or RNA-seq) of both khat and *E*. *sinica* plants. Illumina NGS technology remains the most popular platform owing to its ever-growing read lengths and overall number of reads per run, allowing both transcriptome assembly in the absence of a reference genome (*de novo* assembly) and quantitative gene expression analysis [15]. The results of this study provide an unprecedented opportunity to identify novel alkaloid biosynthetic genes and quantify gene expression. Further, we evaluate the performance of two different assembly programs, Trinity *de novo* RNA-seq assembler [16] and Velvet-Oases v0.1.16 [17].

## Materials and Methods

### Plant materials

Khat shrubs (*Catha edulis*, Forsk.) cv “Mahanaim” were grown in open field conditions under commercial growing practices, including drip irrigation and fertilization, at the Newe Ya’ar Research Center in Northern Israel. The shrubs were approximately 10 years old at the time of harvest. The *Ephedra sinica* plants used in this study were germinated from seeds acquired from wild, openly pollinated varieties originating in Northern China (Horizon Herbs, OR, USA). Cultivation was carried out in Canada at the Southern Crop Protection and Food Research Centre (SCPFRC; London, Ontario), where plants were grown under standard greenhouse conditions in pots containing a 50:50 blend of sand and commercial cactus soil mix.

### Poly(A)+ RNA purification, cDNA library preparation and Illumina GA sequencing

Young khat leaves, approximately 1–3 cm in length, were harvested during daylight hours, and total RNA was isolated using an RNeasy Midi kit (Qiagen). For *E*. *sinica*, freshly emerging, light green “young” stems up to 5 cm in length were harvested for total RNA extraction using essentially the same procedure as for the khat tissue. Poly(A)+ RNA purification, cDNA library preparation, emulsion-based PCR (emPCR) and sequencing were performed at the McGill University and Génome Québec Innovation Center (Montréal, Canada). RNA quality was assessed using an RNA 6000 Nano chip on a BioAnalyzer 2100 (Agilent Technologies) to ensure an RNA Integrity Number (RIN) of > 7.5. Poly(A)+ RNA was prepared using a Dynabead mRNA Purification kit (Invitrogen). cDNA library preparation and Illumina GA sequencing were performed as described [18].

### *De novo* transcriptome assembly

Sequence quality control and cleaning were performed as described [18]. Short-read sequence data corresponding to khat cultivated at the Newe Ya’ar Research Center were assembled using the Trinity *de novo* RNA-seq assembler [16]. In contrast, data corresponding to *E*. *sinica* plants cultivated at SCPFRC were assembled using both Trinity *de novo* RNA-seq assembler and Velvet-Oases v0.1.16 [17], respectively, generating two distinct libraries. The parameters used for Trinity-based assemblies (module “Butterfly”) were as follows: graph compaction option: edge-thr = 0.26; path extension mode = compatible_path_extention; min_contig_length = 300; paired_fragment_length = 270 (50 bp + estimated median fragment size of readset). Standard settings were used otherwise. The Velvet-based assembly was performed as described previously [18].

### Functional annotation and GO analysis

Annotation of the three assembled transcriptome datasets was performed using the Magpie Automated Genomics Project Investigation Environment (MAGPIE) as described [18,19]. Briefly, MAGPIE automates sequence similarity searches against major public and local target databases. The TimeLogic Tera-BLAST algorithm (http://www.timelogic.com) was used to compare transcripts to the NCBI database NR (non-redundant) and the viridiplantae subset of RefSeq [20]. An expected e-value of 1e-3 and a minimum alignment length of 30 bp were used. Information regarding sequence motifs was collected using accelerated Hidden Markov Model (HMM) searches against local instances of Interpro HMM libraries at an e-value of 1e-10. The NCBI Conserved Domain Database (CDD) was also queried for further structural information. Finally, a functional description was assigned to each contig, based on a weighted summary of all search results. GO annotations were compiled from GIDs extracted from “level 1” evidence (i.e. annotations based on BLAST matches > 1e-35, HMM matches > 1e-20, and sequence similarities > 65%). GO terms assigned to each contig were used to sort the contigs into one of 13 functional categories, including “secondary metabolism” (ID GO:0019748) and an “unknown” category comprised of contigs assigned to the GO term “Biological Process Unknown” (ID GO:0008150). In addition contigs that did not match to any GO term were grouped separately. All the raw transcriptome data have been deposited in the NCBI Short Read Archive (SRA).

### Identification and expression analyses of gene candidates

Khat and *E*. *sinica* databases (CED-Trinity and ESI-Velvet, respectively) were searched for contigs representing genes potentially involved in phenylpropylamino alkaloid biosynthesis. ESI-Trinity was not queried in the first round, since contigs in this database were relatively short compared with those found in ESI-Velvet. Full-length query sequences representing functionally characterized enzymes were used for tBLASTn searches. When available, gymnosperm sequences were used to query ESI-Velvet, whereas only angiosperm sequences were used to query CED-Trinity. Of the 40 gene candidates identified from CED-Trinity, two were comprised of more than one contig, which were assembled manually based on obvious regions of identity. All *E*. *sinica* gene candidates corresponded to auto-assembled contigs or singlets. To assign FPKM values to *E*. *sinica* gene candidates (available only through Trinity-based assembly) a second round of tBLASTn was performed using gene candidates identified in ESI-Velvet to query ESI-Trinity. In cases where a single query revealed more than one identical match in ESI-Trinity, a single FPKM was re-calculated representing all contigs within the group.

### Phylogenetic analysis

Amino acid alignments were performed using ClustalW [21], and phylogenetic trees were built from bootstrap values generated using Geneious software package (Biomatters, Newark, NJ). GenBank accession numbers for sequences used to construct the trees are as follows: BDH outgroup, NP_035853.2; AmBDH *Antirrhinum majus*, ACM89738.1; AtBDH *Arabidopsis thaliana*, NP_563711; BL outgroup, Q5SKN9.1; BL *Arabidopsis thaliana*, NP_176763.1; 4CL outgroup, Q336M7.3; 4CL *Arabidopsis thaliana*, Q42524.1; 4CL *Nicotiana tabacum*, O24145.1; 4CL *Petroselinum crispum*, P14912.1; 4CL *Pinus taeda*, AAA92669.1; 4CL *Solanum tuberosum*, P31684.1; KAT outgroup, P45362.2; 2WUA; KAT *Arabidopsis thaliana*, 2WU9; KAT *Helianthus annuus*, KAT *Petunia x hybrida*, ACV70032.1; NMT outgroup, Q5C9L6.1; NMT *Arabidopsis thaliana* 1, NP_196113; NMT *Arabidopsis thaliana* 2, NP_199713; NMT *Atropa belladonna*, BAA82264; NMT *Coffea arabica*, BAC75663; NMT *Papaver somniferum*, AAY79177; NMT *Solanum lycopersicon*, AAG59894; PAL outgroup, P10248.2; PAL *Arabidopsis thaliana*, P35510.3; PAL *Cunninghamia lanceolata*, AFX98070.1; PAL *Ephedra sinica*, AB300199.1; PAL *Eucalyptus robusta*, BAL49995.1; PAL *Larix kaempferi*, AHA44840.1; PAL *Petroselinium crispum*, P45729.1; PAL *Pinus taeda*, P52777.1; PAL *Selaginella moellendorffii*, XP_002982907.1; RED outgroup, P39640.2; RED *Datura stramonium* TRI, TRI_L20473; RED *Datura stramonium* TRII, RED *Eschscholzia californica*, ADE41047.1; TRII_L20474; RED *Hyoscyamus niger* TRI, P50164.1; RED *Papaver somniferum*, AAF13739.1; TA outgroup, Q4J8X2.1; CmTA *Cucumis melo*, ADC45389.1; TA *Papaver somniferum*, ADC33123.1; PxhTA *Petunia x hybrida*, E9L7A5.1; PpTA *Pinus pinaster*, CAF31327.1; ThDPC outgroup, P27868.1; AtAHAS *Arabidopsis thaliana*, ABJ80681.1; AtPDC *Arabidopsis thaliana*, NP_200307.1; BnAHAS *Brassica napus*, P27818.1; NtPDC *Nicotiana tabacum*, P51846.1; PsPDC *Pisum sativum*, P51850.1; ZmPDC *Zymomonas mobilis*, 1ZPD.

## Results and Discussion

### Tissue selection for enrichment of biosynthetic genes

In khat, pathway intermediates 1-phenylpropane-1,2-dione and (*S*)-cathinone, and end products cathine and (1*R*,2*S*)-norephedrine, accumulate mainly in young leaves and flowers with lesser quantities in young stems [12,13]. In contrast, mature leaves lack (*S*)-cathinone and accumulate only cathine and (1*R*,2*S*)-norephedrine suggesting that alkaloid biosynthetic gene expression is highest in young tissues. For this reason, young khat leaves were selected for RNA extraction. Precursors 1-phenylpropane-1,2-dione and (*S*)-cathinone are present in young *E*. *sinica* stems, but not mature stems or roots [5]. In contrast, downstream metabolites including cathine, (1*R*,2*S*)-norephedrine, *N*-methylated or *N*,*N*-dimethylated products occur in both young and mature stems, although accumulation is much greater in mature stems. This pattern suggests that alkaloid biosynthesis is carried out predominantly in younger tissue, resulting in the presence of end products in older tissue as the stems elongate. We targeted a transciptome presumably enriched in alkaloid biosynthetic gene transcripts by choosing young *E*. *sinica* stems for RNA extraction. Marked diversity in alkaloid stereochemical composition has been noted for different *E*. *sinica* accessions. For example, some varieties accumulate only 1*R* isomers, whereas others accumulate both 1*S* and 1*R* forms [5]. Assaying for (*S*)-cathinone reductase activity in extracts of these *E*. *sinica* accessions revealed that the proportion of (1*S*,2*S*)-cathine and/or (1*R*,2*S*)-norephedrine product corresponded directly with plant accumulation profiles, supporting the existence of stereospecific reductases. The *E*. *sinica* used in this study originates with seeds harvested from wild populations native to the steppes of north and northwestern China, and accumulates both isomers. Therefore, if stereospecific reactions are involved, representation for enzymes favoring both 1*S* and 1*R* epimers is expected.

### Transcriptome sequencing, assembly and annotation

RNA was screened for sufficient quality (S1 Fig.) prior to Illumina sequencing to generate 272,586,558 and 191,352,154 short sequence reads for khat and *E*. *sinica*, respectively (Table 1). Quality filtering these data yielded “clean” reads representing 79% (khat) and 86% (*E*. *sinica*) of the original number of raw reads. We initially opted to use the newer Trinity *de novo* RNA-seq software [16] instead of an older package, Velvet-Oases v0.1.16 [17], to assemble khat and *E*. *sinica* transcriptomes. Comparison of Trinity and Velvet-Oases platforms has found that the Trinity assembler is superior at resolving splice alternates, and produces less duplicates or assembly chimeras, which are often introduced in a Velvet-Oases multi-run merging stage, hence generating a lower total number of long reads than Velvet-Oases [22]. Furthermore, Velvet-Oases v.0.1.16 [17] does not allow calculation of FPKM (fragments mapped per kilobase of exon per million reads mapped), a normalizing statistic measuring gene expression while accounting for variation in gene length [23]. It is noteworthy that newer versions of Velvet-Oases [24] (https://www.ebi.ac.uk/~zerbino/oases/), which were not available at the time of assembly, are routinely used to calculate normalizing statistics. Trinity-based assembly of khat and *E*. *sinica* libraries yielded 77,290 and 63,344 unigenes respectively, proportionately in line with the number of reads (CED-Trinity and ESI-Trinity; Table 1). However, it was discovered during full-length coding sequence (CDS) analysis that unlike CED-Trinity where half (50%) of the unigenes represented full-length clones, less than one third (29%) of ESI-Trinity unigenes contained a complete CDS. BLAST searches of CED-Trinity for gene candidates using full-length homologues from various plant species yielded a modest number of hits per query (<10) (Table 2), but searches of ESI-Trinity yielded large lists of hits (>50), many of which were identical except for 1 or 2 base pairs (data not shown). A possible reason for the ability of Trinity to assemble full-length clones for khat data but not *E*. *sinica* data is the occurrence of single nucleotide polymorphisms (SNPs) within *E*. *sinica* transcripts. Unlike the khat tissue, which derived from a single commercial cultivar with a long history of breeding, the *E*. *sinica* plants used in this study were derived from wild populations possibly representing more than one accession. Nonetheless, a marked lack of fully assembled, full-length contigs in ESI-Trinity impeded our ability to reliably identify candidate biosynthetic genes. Therefore, Velvet-Oases was used to assemble a third library, ESI-Velvet, which contained 59,448 unigenes representing a larger number (41,671) of CDSs (Table 1). BLAST performed with ESI-Velvet assemblies showed a greater number of full-length contigs and fewer unassembled singlets compared with ESI-Trinity (Table 3), and were thus used for gene candidate searches.

**Table 1:** Summary of the construction and assembly for three Illumina NGS libraries.

Abbrev.	Plant	Tissue	SRA accession number	No. of raw reads	No. of cleaned reads	Average transcript read depth (reads/bp)	Unigenes	Predicted no. of full-length CDS
CED-Trinity	*Catha edulis*	Young leaf	SRX485764	272,586,558	215,532,092	119.0	77,290	38,322
ESI-Trinity	*Ephedra sinica*	Shoot tip	SRX485643	191,352,154	164,533,710	121.3	63,344	18,342
ESI-Velvet	*Ephedra sinica*	Shoot tip	SRX485643	191,352,154	164,533,710	62.9	59,448	41,671

Abbreviations: CED, *Catha edulis*; ESI, *Ephedra sinica*; SRA, short-read archive; CDS, coding sequence.

**Table 2:** Khat unigenes representing enzymes putatively involved in ephedrine alkaloid biosynthesis.

Identifier	Database ID	Candidate reaction	Identity	Query	Accession number
CePAL1–1	comp191_c0_seq1	PAL	592/729 (81%)	AtPAL1	P35510.3
CePAL1–2	comp2625_c0_seq1	PAL	491/513 (95%)	AtPAL1	P35510.3
Ce4CL1–1	comp2198_c0_seq1	4CL	414/563 (73%)	At4CL1	Q42524.1
Ce4CL1–2	comp1320_c0_seq1	4CL	396/564 (70%)	At4CL1	Q42524.1
Ce4CL1–3	comp24405_c0_seq1	4CL	343/573 (59%)	At4CL1	Q42524.1
CeBDH1–1	comp9812_c0_seq2	BDH	710/1367 (51%)	AtAAO4	NP_563711
CeBDH2–1	comp1221_c0_seq1	BDH	423/537 (78%)	AmBALDH	ACM89738.1
CeBDH2–2	comp15067_c0_seq1	BDH	360/535 (67%)	AmBALDH	ACM89738.1
CeBDH2–3	comp6403_c0_seq2	BDH	276/534 (51%)	AmBALDH	ACM89738.1
CeCHD1–1	comp2279_c1_seq1	CHD	579/724 (79%)	PhCHD	JX142126.1
CeCHD1–2	comp1276_c0_seq1	CHD	423/728 (58%)	PhCHD	JX142126.1
CeKAT1–1	comp4442_c0_seq2	KAT	368/464 (79%)	PhKAT1	ACV70032.1
CeKAT1–2	comp3066_c1_seq1	KAT	363/463 (78%)	PhKAT1	ACV70032.1
CeBL1–1	comp2535_c0_seq1	BL	347/581 (59%)	AtBZO1	NP_176763.1
CeBL1–2	comp13850_c0_seq1	BL	273/585 (46%)	AtBZO1	NP_176763.1
CeBL1–3	comp18626_c0_seq1	BL	268/625 (42%)	AtBZO1	NP_176763.1
CeBL1–4	comp10832_c0_seq1	BL	273/583 (46%)	AtBZO1	NP_176763.1
CeBL1–5	comp3351_c0_seq1	BL	271/584 (46%)	AtBZO1	NP_176763.1
CeBL1–6	comp5650_c0_seq1	BL	262/591 (44%)	AtBZO1	NP_176763.1
CeThDPC1–1	comp2318_c0_seq1	ThDPC	513/678 (75%)	AtAHAS	ABJ80681.1
CeThDPC2–1	comp2120_c0_seq1	ThDPC	506/608 (83%)	AtPDC2	NP_200307.1
CeThDPC2–2	Manual Assembly	ThDPC	468/608 (76%)	AtPDC2	NP_200307.1
CeTA1–1	comp1545_c0_seq1	TA	368/480 (76%)	PhPPA-AT	E9L7A5.1
CeTA1–2	comp1191_c0_seq1	TA	128/490 (26%)	PhPPA-AT	E9L7A5.1
CeTA1–3	comp29507_c0_seq1	TA	120/493 (24%)	PhPPA-AT	E9L7A5.1
CeTA2–1	comp7370_c0_seq1	TA	328/419 (78%)	CmArAT1	ADC45389.1
CeTA2–2	comp25340_c0_seq1	TA	253/427 (59%)	CmArAT1	ADC45389.1
CeRED1–1	comp5248_c0_seq1	RED	155/273 (56%)	DsTRI	AAA33281.1
CeRED1–2	comp6446_c0_seq1	RED	155/280 (55%)	DsTRI	AAA33281.1
CeRED1–3	comp24582_c0_seq1	RED	148/273 (54%)	DsTRI	AAA33281.1
CeRED1–4	comp24893_c0_seq1	RED	147/278 (52%)	DsTRI	AAA33281.1
CeRED1–5	comp601_c1_seq1	RED	143/284 (50%)	DsTRI	AAA33281.1
CeRED1–6	comp2523_c1_seq1	RED	137/273 (50%)	DsTRI	AAA33281.1
CeRED1–7	Manual Assembly	RED	140/276 (50%)	DsTRI	AAA33281.1
CeRED2–1	comp1063_c0_seq1	RED	179/324 (55%)	PsCOR1	AAF13739.1
CeRED2–2	comp7109_c0_seq1	RED	180/382 (47%)	PsCOR1	AAF13739.1
CeRED2–3	comp7266_c0_seq1	RED	148/323 (45%)	PsCOR1	AAF13739.1
CeRED2–4	comp33459_c0_seq1	RED	130/327 (39%)	PsCOR1	AAF13739.1
CeRED2–5	comp3071_c2_seq1	RED	123/328 (37%)	PsCOR1	AAF13739.1
CeRED2–6	comp1646_c0_seq1	RED	118/331 (35%)	PsCOR1	AAF13739.1
CeRED3–1	comp673_c0_seq1	RED	176/274 (64%)	EcSanR	ADE41047.1
CeRED3–2	comp4872_c0_seq1	RED	86/372 (23%)	EcSanR	ADE41047.1

Each unigene is assigned an identifier, which corresponds to a database ID in the CED-Trinity library. Percent amino acid identity between unigenes and queries is provided. Abbreviations: AAO4, aromatic aldehyde oxidase 4; Am, *Antirrhinum majus*; AHAS, acetohydroxyacid synthase; ArAT, aromatic amino acid transaminase; At, *Arabidopsis thaliana*; BALDH, benzaldehyde dehydrogenase; BDH, benzaldehyde dehydrogenase; BL, benzoate CoA-ligase; BZO, benzoyloxyglucosinolate; CHD, cinnamoyl-CoA hydratase-dehydrogenase; 4CL, 4-coumaroyl-CoA ligase; Ce, *Catha edulis*; Cm, *Cucumis melo*; COR, codeinone reductase; Ds, *Datura stramonium*; Ec, *Eschscholzia californica*; Es, *Ephedra sinica*; KAT, 3-ketoacyl-CoA thiolasae; Ps, *Papaver somniferum*; PAL, L-phenylalanine ammonia lyase; PDC, pyruvate decarboxylase; Ph, *Petunia x hybrida*; PPA-AT, prephenate aminotransferase; RED, reductase; SanR, sanguinarine reductase; TA, transaminase; ThDPC, thiamin diphosphate-dependent carboligase; TR, tropinone reducase.

**Table 3:** *Ephedra sinica* unigenes representing enzymes putatively involved in ephedrine alkaloid biosynthesis.

Identifier	Database ID	Candidate reaction	Identity	Query	Accession number
EsPAL1–1	Contig5	PAL	718/723 (99%)	EsPAL1	AB300199.1
EsPAL1–2	Contig20298	PAL	445/728 (61%)	EsPAL1	AB300199.1
Es4CL1–1	Contig18937	4CL	368/545 (67%)	Pt4CL	AAB42382
Es4CL1–2	Contig547	4CL	350/554 (63%)	Pt4CL	AAB42382
Es4CL1–3	Contig5701	4CL	353/551 (64%)	Pt4CL	AAB42382
EsBDH1–1	Singlet106372	BDH	657/1444 (45%)	AtAAO4	NP_563711
EsBDH1–2	Singlet88157	BDH	332/1346 (24%)	AtAAO4	NP_563711
EsBDH1–3	Contig2002	BDH	427/1423 (30%)	AtAAO4	NP_563711
EsBDH2–1	Contig9169	BDH	431/536 (80%)	AmBALDH	ACM89738.1
EsBDH2–2	Singlet9479	BDH	396/541 (73%)	AmBALDH	ACM89738.1
EsBDH2–3	Contig9201	BDH	396/545 (72%)	AmBALDH	ACM89738.1
EsBDH2–4	Contig12745	BDH	292/535 (54%)	AmBALDH	ACM89738.1
EsBDH2–5	Contig22940	BDH	290/537 (54%)	AmBALDH	ACM89738.1
EsCHD1–1	Contig833	CHD	351/724 (48%)	PhCHD	JX142126.1
EsCHD1–2	Singlet85794	CHD	427/731 (58%)	PhCHD	JX142126.1
EsCHD1–3	Singlet68699	CHD	376/725 (51%)	PhCHD	JX142126.1
EsKAT1–1	Contig6801	KAT	365/463 (78%)	PhKAT1	ACV70032.1
EsKAT1–2	Contig24534	KAT	344/463 (74%)	PhKAT1	ACV70032.1
EsKAT1–3	Contig31090	KAT	329/466 (70%)	PhKAT1	ACV70032.1
EsKAT1–4	Contig2319	KAT	307/463 (66%)	PhKAT1	ACV70032.1
EsKAT1–5	Singlet7228	KAT	298/462 (64%)	PhKAT1	ACV70032.1
EsBL1–1	Contig433	BL	312/603 (51%)	AtBZO1	NP_176763.1
EsBL1–2	Contig10250	BL	305/593 (51%)	AtBZO1	NP_176763.1
EsBL1–3	Singlet7007	BL	273/582 (46%)	AtBZO1	NP_176763.1
EsBL1–4	Contig33805	BL	281/586 (47%)	AtBZO1	NP_176763.1
EsBL1–5	Contig6255	BL	265/584 (45%)	AtBZO1	NP_176763.1
EsBL1–6	Singlet85705	BL	246/593 (41%)	AtBZO1	NP_176763.1
EsThDPC1–1	Contig5434	ThDPC	457/671 (68%)	AtAHAS	ABJ80681.1
EsThDPC1–2	Contig23037	ThDPC	413/672 (61%)	AtAHAS	ABJ80681.1
EsThDPC2–1	Contig35903	ThDPC	454/610 (74%)	AtPDC2	NP_200307.1
EsThDPC2–2	Contig5589	ThDPC	250/608 (41%)	AtPDC2	NP_200307.1
EsTA1–1	Contig4104	TA	309/485 (63%)	PhPPA-AT	E9L7A5.1
EsTA1–2	Singlet66014	TA	237/486 (48%)	PhPPA-AT	E9L7A5.1
EsTA2–1	Contig13244	TA	187/416 (44%)	CmArAT1	ADC45389.1
EsTA2–2	Singlet18529	TA	175/413 (42%)	CmArAT1	ADC45389.1
EsTA2–3	Singlet87150	TA	178/416 (42%)	CmArAT1	ADC45389.1
EsRED1–1	Contig36780	RED	90/274 (32%)	DsTRI	AAA33281.1
EsRED1–2	Contig143	RED	82/293 (27%)	DsTRI	AAA33281.1
EsRED1–3	Contig14099	RED	73/313 (23%)	DsTRI	AAA33281.1
EsRED1–4	Contig19440	RED	82/278 (29%)	DsTRI	AAA33281.1
EsRED1–5	Singlet71271	RED	83/288 (28%)	DsTRI	AAA33281.1
EsRED1–6	Contig16252	RED	78/292 (26%)	DsTRI	AAA33281.1
EsRED2–1	Contig30213	RED	66/261 (25%)	DsTRII	AAA33282
EsRED2–2	Singlet88092	RED	76/266 (28%)	DsTRII	AAA33282
EsRED2–3	Singlet14224	RED	49/262 (18%)	DsTRII	AAA33282
EsRED3–1	Singlet88290	RED	132/327 (40%)	PsCOR1	AAF13739.1
EsRED3–2	Singlet12882	RED	133/325 (40%)	PsCOR1	AAF13739.1
EsRED3–3	Singlet16920	RED	122/341 (35%)	PsCOR1	AAF13739.1
EsRED3–4	Contig35277	RED	124/331 (37%)	PsCOR1	AAF13739.1
EsRED3–5	Contig20961	RED	120/357 (33%)	PsCOR1	AAF13739.1
EsRED4–1	Contig37733	RED	165/274 (60%)	EcSanR	ADE41047.1
EsNMT1–1	Singlet3659	NMT	152/360 (42%)	PsTNMT	AAY79177
EsNMT2–1	Singlet112119	NMT	354/497 (71%)	SlPEAMT	AAG59894
EsNMT2–2	Contig24415	NMT	312/496 (62%)	SlPEAMT	AAG59894
EsNMT3–1	Contig17099	NMT	150/395 (37%)	CaCS	BAC75663
EsNMT3–2	Contig29630	NMT	120/406 (29%)	CaCS	BAC75663
EsNMT3–3	Contig17536	NMT	131/397 (32%)	CaCS	BAC75663
EsNMT3–4	Contig1597	NMT	118/401 (29%)	CaCS	BAC75663
EsNMT4–1	Contig29277	NMT	186/341 (54%)	AbPMT	BAA82264
EsNMT4–2	Contig12525	NMT	176/354 (49%)	AbPMT	BAA82264
EsNMT5–1	Contig6426	NMT	242/689 (35%)	AtSUVH	NP_196113
EsNMT5–2	Contig4615	NMT	228/715 (31%)	AtSUVH	NP_196113
EsNMT5–3	Contig8760	NMT	227/680 (33%)	AtSUVH	NP_196113
EsNMT6–1	Contig20402	NMT	330/563 (58%)	AtPRMT	NP_199713
EsNMT6–2	Contig29347	NMT	127/530 (23%)	AtPRMT	NP_199713
EsNMT6–3	Contig36061	NMT	117/548 (21%)	AtPRMT	NP_199713
EsNMT6–4	Singlet15333	NMT	113/561 (20%)	AtPRMT	NP_199713
EsNMT6–5	Singlet13096	NMT	124/661 (18%)	AtPRMT	NP_199713

Each unigene is assigned an identifier, which corresponds to a database ID in the ESI-Velvet library. Percent amino acid identity between unigenes and queries is provided. Abbreviations: AAO4, aromatic aldehyde oxidase 4; Ab, *Atropa belladonna*; Am, *Antirrhinum majus*; AHAS, acetohydroxyacid synthase; ArAT, aromatic amino acid transaminase; At, *Arabidopsis thaliana*; BALDH, benzaldehyde dehydrogenase; BDH, benzaldehyde dehydrogenase; BL; benzoate CoA ligase; BZO, benzoyloxyglucosinolate; Ca, *Caffea arabica*; Ce, *Catha edulis*; CHD, cinnamoyl-CoA hydratase-dehydrogenase; 4CL, 4-coumaroyl-CoA ligase; Cm, *Cucumis melo*; CS, caffeine synthase; COR, codeinone reductase; Ds, *Datura stramonium*; Ec, *Eschscholzia californica*; Es, *Ephedra sinica*; KAT, 3-ketoacyl-CoA thiolasae; NMT, *N*-methyltransferase; PEANMT, phosphoethanolamine *N*-methyltransferase; PMT, putrescine *N*-methyltransferase; Ps, *Papaver somniferum*; PAL, L-phenylalanine ammonia lyase; PDC, pyruvate decarboxylase; Ph, *Petunia x hybrida*; PPA-AT, prephenate aminotransferase; PRMT, protein arginine *N*-methyltransferase; Pt, *Pinus taeda*; RED, reductase; SanR, sanguinarine reductase; Sl, *Solanum lycopersicon*; SUVH, histone lysine *N*-methyltransferase, H3L9-specific; TA, transaminase; ThDPC, thiamin diphosphate-dependent carboligase; TNMT, (*S*)-tetrahydroprotoberberine *N*-methyltransferase; TR, tropinone reducase.

**Fig 2:**
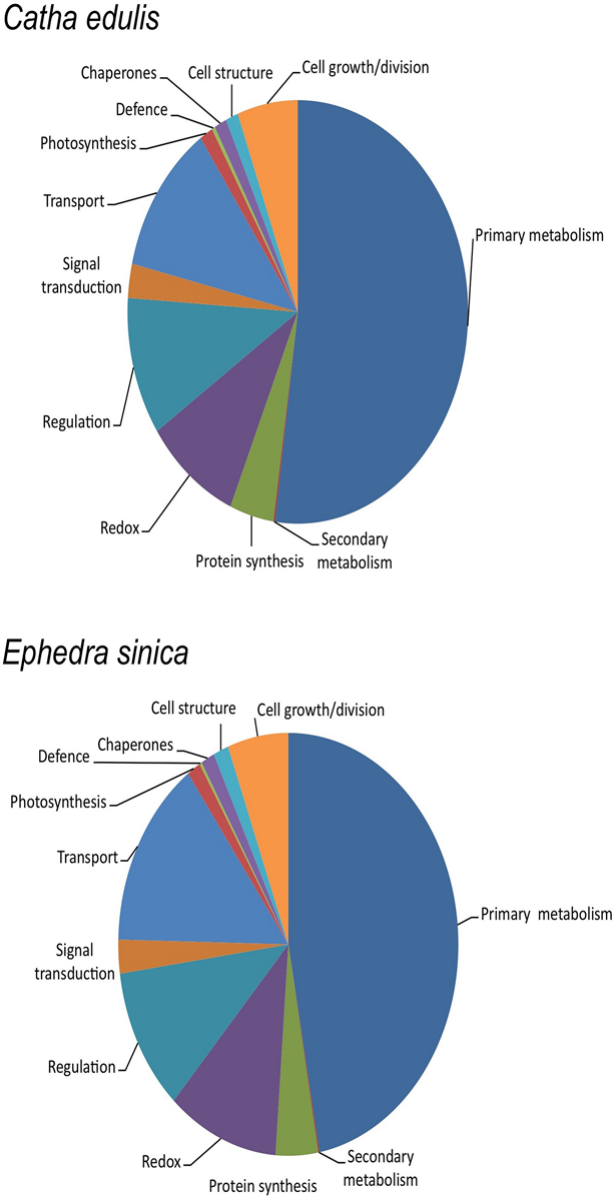
Functional category analysis based on Gene Ontology (GO) annotations of CED-Trinity (upper panel) and ESI-Trinity (lower panel) unigenes. Results for ESI-Velvet are found in S2 Fig.

The MAGPIE-based pipeline used to annotate the three libraries included gene- and domain-level searches against NCBI and RefSeq [20] databases, in addition to Hidden Markov Models searches [18]. All search results were summarized and weighted to arrive at a single annotation per unigene. Additionally, GO (Gene Ontology) annotations were assigned to individual contigs, based on continually updated criteria (http://www.geneontology.org). Fig. 2 illustrates the GO annotation results for CED-Trinity and ESI-Trinity databases. The GO pie chart for ESI-Velvet is found in S2 Fig. GO annotation analysis yielded very similar results for all three libraries, with primary metabolism comprising the largest category of unigenes (Fig. 2, S2 Fig.). The GO analysis results for CED-Trinity are very similar to those reported for a Sanger sequencing-based khat cDNA library [14]. This finding is not surprising, as source tissue was the same in both cases.

### Evidence for benzoic acid metabolism in khat and *Ephedra sinica*

**Fig 3:**
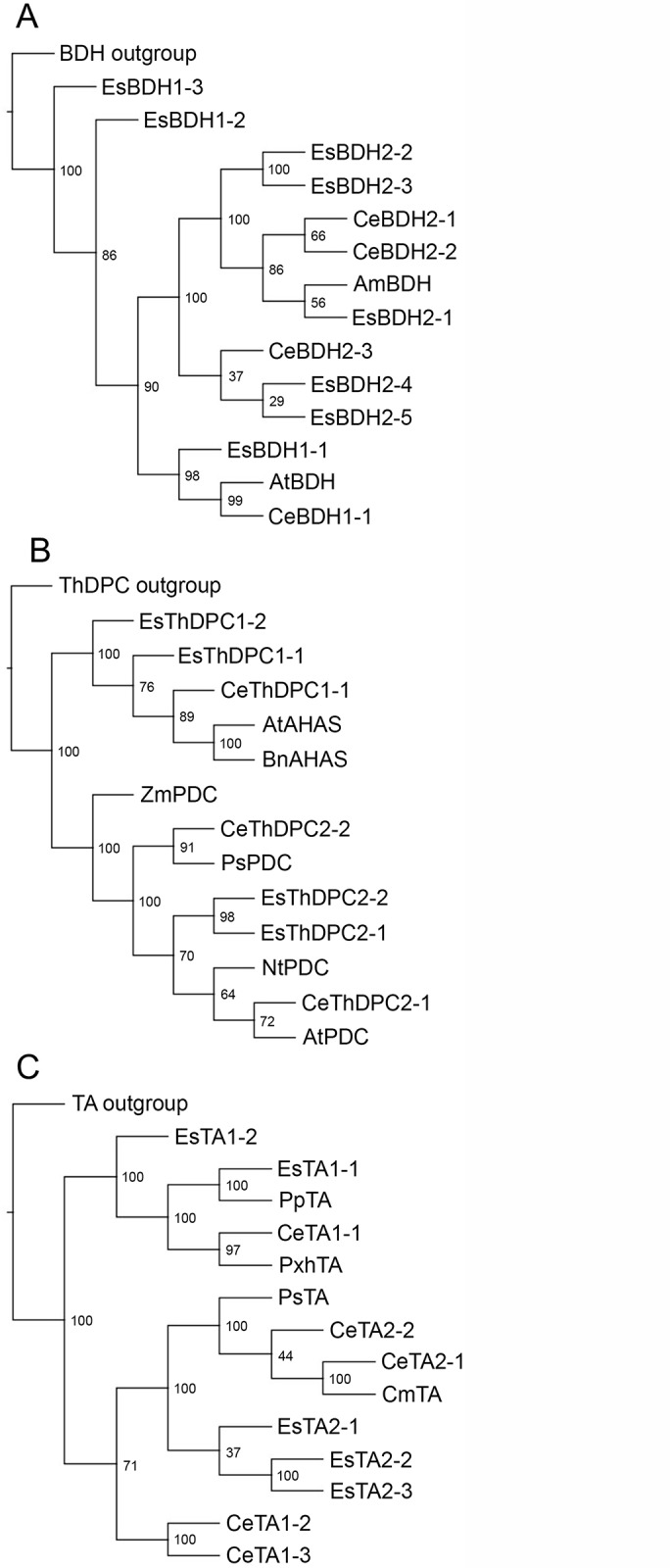
Phylogenetic analysis of gene candidates. Abbreviations: benzaldehyde dehydrognase (BDH) (A), thiamin diphosphate-dependent carboligase (ThDPC) (B) and transaminase (TA) (C). Similar analyses for remaining candidates are found in S3 Fig. Sequences were aligned and analyzed for phylogenetic relationships using the neighbor-joining algorithm. Numbers at each node represent bootstrap values calculated using 1000 iterations. Accession numbers are found under Experimental section, and abbreviations are defined in Tables 2 and 3.

**Fig 4:**
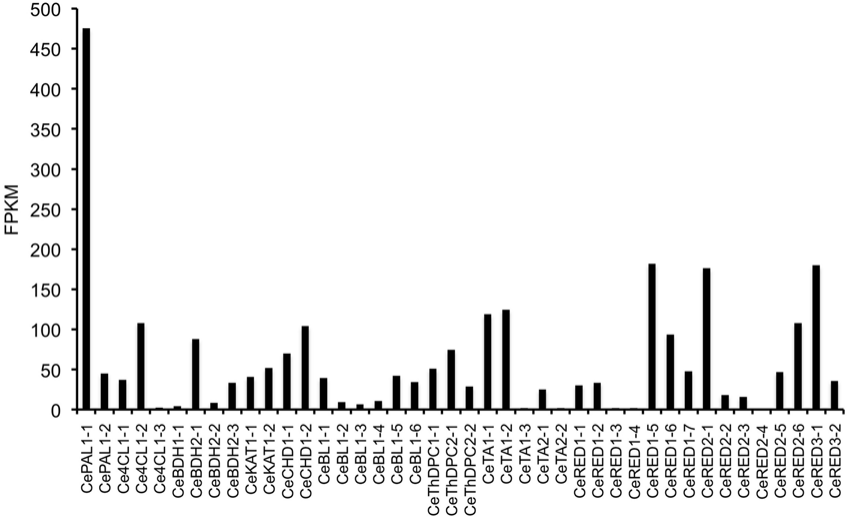
Relative expression of gene candidates identified in khat (CED-Trinity). FPKM (fragments mapped per kilobase of exon per million reads mapped) is a normalizing statistic measuring gene expression while accounting for variation in gene length [23]. Abbreviations are defined in Tables 2 and 3.

Toward the goal of identifying genes putatively involved in ephedrine alkaloid metabolism, khat and *E*. *sinica* libraries were queried using amino acid sequences corresponding to enzymes of known function. Search results are listed in Table 2 (khat) and Table 3 (*E*. *sinica*) with accompanying phylogenetic analyses presented in Fig. 3 and S3 Fig. Full sequences of these search results are listed in S1 Dataset and S2 Dataset for khat and *E*. *sinica*, respectively. Complete sequences of the queries used for data mining of khat and *E*. *sinica* libraries are found in S3 Dataset and S4 Dataset, respectively. As illustrated in Fig. 1, many steps of benzoic acid metabolism have been characterized at the molecular genetic level from such plants as *Arabidopsis thaliana*, snapdragon (*Antirrhinum majus*) and petunia (*Petunia x hybrida*). This enabled the use of queries with established roles in the pathway, at least in source plant species, to find putative homologues in khat and *E*. *sinica*. In some cases, more than one enzyme is known to catalyze a particular step; for example, dehydrogenation of benzaldehyde to benzoic acid may occur in *Arabidopsis* seeds through aldehyde oxidase-4 (AtAAO4) [25] or via benzaldehyde dehydrogenase in snapdragon (AmBALDH) [26]. Previous analysis of a modest (3,293 unigenes) khat transcriptomic library indicated that only a AmBALDH homologue was present, suggesting a pathway more similar to snapdragon petals than seeds of *Arabidopsis* [14]. The more extensive sequencing performed in this study revealed the presence of both AtAAO4 (CeBDH1–1) and AmBALDH (CeBDH2–1, 2–3, 2–4) homologues in CED-Trinity (Table 2). Phylogenetic analysis of dehydrogenase candidates distinguished these khat sequences in separate clades (Fig. 3A). Nonetheless, CeBDH1–1 identity with AtAA04 was low (51%) compared with that between AmBALDH and the top khat homologue CeBDH2–1 (78%). Relative expression of CeBDH2–1 was > 80-fold greater than that of CeBDH1–1 (Fig. 4), perhaps underscoring an important role for BALDH activity in khat leaves. In *E*. *sinica*, a similar pattern emerged where a close homologue was found for AmBALDH (EsBDH2–1, 80% identity), but not for AtAA04 (Table 3).

Khat sequences closely related (~80% identity) to petunia cinnamoyl-CoA hydratase-dehydrogenase (PhCHD) and 3-ketoacyl-CoA thioliase (PhKAT1) were identified (Table 2) suggesting that a β-oxidative, CoA-dependent pathway is also operative in this species. Several putative PhKAT1 homologues were identified in *E*. *sinica*, ranging in identity up to 78% (Table 3). Less identity (<60%) was observed between *E*. *sinica* candidates and PhCHD, possibly reflecting an alternative metabolism occurring in this plant. For example, *trans*-cinnamoyl-CoA may be converted to 3-oxo-3-phenylpropionyl-CoA by two separate enzymes rather than a single bi-functional enzyme. However, sequence divergence could also reflect evolutionary distance between *E*. *sinica*, a gymnosperm [27,28], and flowering plants.

### Carboligase candidates belonging to AHAS and PDC enzyme families

**Fig 5:**
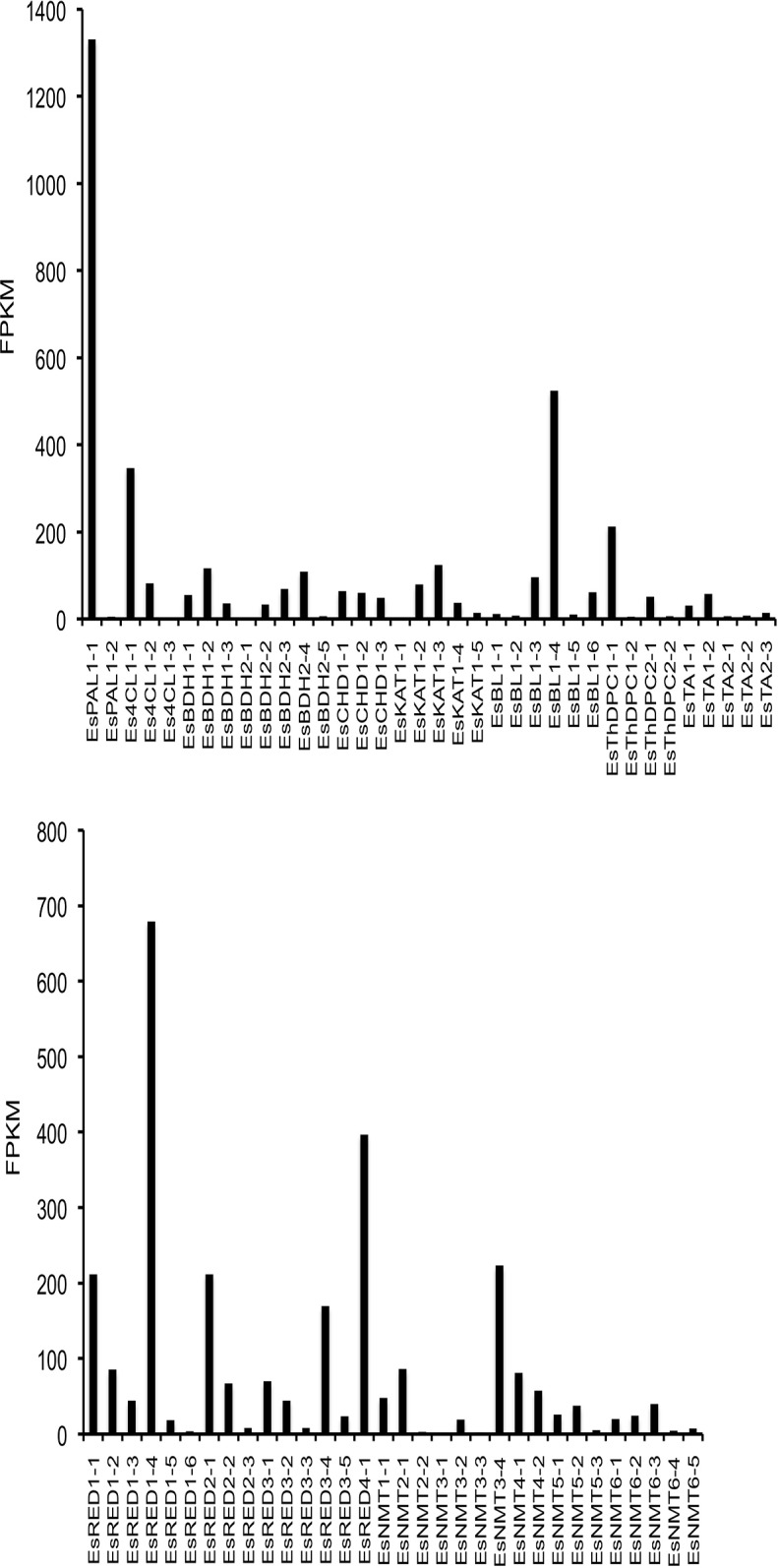
Relative expression of gene candidates identified in *Ephedra sinica* (ESI-Trinity). FPKM (fragments mapped per kilobase of exon per million reads mapped) is a normalizing statistic measuring gene expression while accounting for variation in gene length [23]. Abbreviations are defined in Tables 2 and 3.

While benzoic acid metabolism is largely elucidated at the molecular genetic level, enzymes catalyzing steps predicted to occur beyond this pathway have not been cloned. Therefore, plant enzymes catalyzing mechanistically similar reactions were used as queries to identify khat and *E*. *sinica* genes potentially involved in amphetamine analogue biosynthesis. The first key step in the formation of these alkaloids likely involves carboligation between a C_6_-C_1_ molecule (e.g. benzaldehyde, benzoic acid, or benzoyl-CoA) and pyruvate [29] (Fig. 1). Two distantly related ThPD-dependent enzymes isolated from microbes, acetohydroxyacid synthase (AHAS) and pyruvate decarboxylase (PDC) are known to convert benzaldehyde and pyruvate to (*R*)-phenylacetylcarbinol (R-PAC), an intermediate in the industrial, semi-synthetic production of ephedrine alkaloids. AHAS from diverse species normally catalyzes carboligation of two α-keto acids toward the biosynthesis of branched-chain amino acids. While AHAS from *Escherichia coli* was found to accept benzaldehyde as an alternative substrate [30] it is not known whether plant AHAS isoforms display this substrate flexibility. In contrast with AHAS, carboligation is only a minor side reaction for PDC; the latter enzyme largely performs pyruvate decarboxylation to form acetaldehyde and CO_2_. However, mutational analysis of a PDC from *Zymomonas mobilis* revealed that a single amino acid substitution effectively converted the enzyme from a decarboxylase to a carboligase, thereby creating a variant capable of R-PAC biosynthesis [31]. It is conceivable that PDCs from ephedrine alkaloid-accumulating plants could have acquired carboligation capacity, enabling the production of R-PAC and other C_6_-C_3_ compounds. Recently, extracts of *E*. *sinica* were shown to convert benzaldehyde to R-PAC, in addition to other products [11]. To identify putative ThDP-dependent enzymes responsible for this activity, functionally characterized *Arabidopsis thaliana* AHAS [32] and PDC [33] were used to query khat and *E*. *sinica* libraries. Candidate sequences ranged in identity from 40% to 83% to either query (Tables 2 and 3) and grouped in one of two clades based on similarity to AHAS or PDC (Fig. 3B). FPKM analysis revealed notable differences in expression levels between different ThDP-dependent carboligase candidates in *E*. *sinica*. For example, a 36-fold difference was observed between AHAS homologues EsThDPC1–1 and EsThDPC1–2, and a nearly 10-fold difference was noted between PDS homologues EsThDPC2–1 and EsThDPC2–2 (Fig. 5). Candidates identified in the khat library did not display marked differences in relative expression levels (Fig. 4).

### Evaluation of transaminase candidates

Ephedrine alkaloid biosynthesis occurring through 1-phenylpropane-1,2-dione intermediate would require transamination yielding (*S*)-cathinone (Fig. 1). The *in vitro* production of alternative C_6_-C_3_ structures such as R-PAC in *E*. *sinica* extract [11] raises the question of whether hydroxyketones can themselves undergo transamination, or whether they must first be oxidized to the diketone 1-phenylpropane-1,2-dione. Plant transaminases aren’t known to accept hydroxyketone substrates; in this case, such activity would yield cathine and (1*R*,2*S*)-norephedrine without the need for a reductase. In contrast, amino transfer reactions involving hydroxyketone substrates and aminoalcohol products have been reported in microorganisms [34,35]. Phylogenetic analysis revealed a distinct clade comprised of khat and *E*. *sinica* transaminase candidates closely related to aromatic amino acid transaminases, such as those from opium poppy and melon (*Cucumis melo*) (Fig. 3C). Opium poppy tyrosine aminotransferase, which also acts on phenylalanine and tryptophan, contributes to benzylisoquinoline alkaloid biosynthesis [36]. In melon (*Cucumis melo*), phenylalanine / tyrosine aminotransferase plays a role in the catabolism of amino acids to aroma volatiles [37]. A second distinct clade grouped khat and *E*. *sinica* sequences with prokaryotic-type amino transferases from pine (*Pinus pinaster*) and petunia (Fig. 3C). Prephenate [38] and aspartate [39] transaminases are two prominent members of the class 1b aspartate aminotransferase family, and both enzymes act on non-aromatic substrates. Based solely on the aromatic nature of C_6_-C_3_ intermediates, it is conceivable that an enzyme more similar to phenylalanine / tyrosine transaminases is involved in ephedrine alkaloid biosynthesis.

### Mining reductase candidates

The involvement of a reductase in amphetamine alkaloid metabolism is supported by the detection of (*S*)-cathinone reductase activity in both khat [12] and *E*. *sinica* [5] extracts. Varieties of *E*. *sinica* accumulating predominantly isomers with a (1*R*,2*S*) configuration exhibited stereoselective (*S*)-cathinone reductase activity yielding (1*R*,2*S*)-norephedrine. On the other hand, reductase activity in varieties of *E*. *sinica* featuring alkaloids with both 1*R* and 1*S* configurations yielded mixed isomer products (i.e. both cathine and (1*R*,2*S*)-norephedrine) (Fig. 1). These results support the existence of at least two stereospecific reductases, a feature observed in other systems such as menthol biosynthesis in peppermint (*Mentha* x *piperita*) [40] and tropane alkaloid biosynthesis in several Solanaceae members, including henbane (*Hyoscyamus niger*) [41] and Jimson weed (*Datura stramonium*) [42]. Two different tropinone reductases (TRI and TRII) form one stereoisomeric product each, either tropine for esterified alkaloids or pseudotropine as a precursor to calystegines. Stereospecific reduction of salutaridine and codeine is observed in opium poppy within the context of morphine biosynthesis [43]. A common feature in all these pathways, including ephedrine alkaloid biosynthesis, is the reduction of a keto group, creating a chiral center featuring a hydroxyl moiety. Queries, including TRI, TRII and codeinone reductase (COR), were drawn from a variety of plants to search khat and *E*. *sinica* databases for stereospecific reductases, with top hits shown in Tables 2 and 3, respectively. Overall, percent identities were low (<60%) compared with results obtained for genes of benzoic acid metabolism and upstream carboligase and transaminase steps (Tables 2, 3). This result was not surprising. Although evidence suggests that benzoic acid biosynthesis takes place in khat and *E*. *sinica* (substrates are expected to be similar, if not identical, to those of query enzymes), diverse alkaloid types have not been reported in these plants. (*S*)-Cathinone is structurally quite different from tropinone, menthone and morphinan alkaloids. Database mining for reductase candidates (and *N*-methyltransferase candidates) was expected to yield many more “false leads” compared with more conserved upstream steps. Further, potentially successful leads cannot be evaluated strictly on the basis of sequence identity to queries.

For comparison, sanguinarine reductase (SanR) from California poppy (*Eschscholzia californica*) was used to query khat and *E*. *sinica* libraries. SanR substrates (e.g. sanguinarine, chelerythrine) are charged, planar, highly aromatic molecules lacking a keto group, and reduction takes place through an alkanolamine intermediate yielding a non-chiral center [44]. Interestingly, querying with SanR revealed closer matches (top hits of 64% and 60% respectively, for khat and *E*. *sinica*) (Tables 2, 3). However, neither the nature of SanR substrates nor the SanR reaction mechanism supports involvement of homologues in ephedrine metabolism.

### Mining *N*-methyltransferase candidates

Alkaloid metabolism in *E*. *sinica* proceeds beyond reduction to include *N*-methylated products, such as diastereomers (1*S*,2*S*)-pseudoephedrine and (1*R*,2*S*)-ephedrine (Fig. 1). *N*,*N*-Dimethylated versions of these alkaloids are also present in *E*. *sinica* [5], suggesting that either a single enzyme performs consecutive *N-*methylations, or additional enzyme(s) are necessary for subsequent steps. Although *S*-adenosylmethionine (SAM)-dependent *N-*methyltransferase (NMT) activity was detected in *E*. *sinica* stem extracts [5], *N*-methylation of ephedrine alkaloids has not been characterized at the molecular level. SAM-dependent *N*-methylation is common to several plant natural product biosynthetic pathways. For example, *N*-methylation of benzylisoquinoline alkaloids by enzymes such as opium poppy (*S*)-tetrahydroprotoberberine *N*-methyltransferase (TNMT) [45] yields tertiary or quaternary amines. Caffeine biosynthesis in coffee (*Coffea arabica*) requires the participation of three closely related NMTs [46]. An early step toward the formation of several plant alkaloids, including nicotine, cocaine, calystegines, atropine and scopolamine, involves *N*-methylation by putrescine *N*-methyltransferase (PMT) [47]. Queries representing TNMT, PMT and caffeine synthase (CS) were used to search the *E*. *sinica* database for homologues, with the top hits shown in Table 3. Sequence identity between queries and hits was generally low, due in part to the evolutionary distance between *E*. *sinica* and the angiosperm species from which queries were drawn. Other reasons include those discussed as part of the reductase mining strategy.

We also considered the possibility that *E*. *sinica* could have recruited an *N*-methyltransferase unrelated to previously described enzymes of plant secondary metabolism. It is generally accepted that enzymes of plant specialized biochemistry arise through divergent evolution; for example, through the recruitment of “rogue” enzymes sustaining mutational changes compared with close homologues in primary metabolism [48]. A prominent example of *N*-methylation in primary metabolism is the mono-, di-, or trimethylation of lysine (Lys) or arginine (Arg) residues of histones and other proteins or peptides [49]. *N*-Methylation of histone Lys and Arg side chain amino groups forms a crucial part of the histone code. Proteins other than histones undergo similar modifications. For example, the ε-amino group of Lys-14 in the large subunit of Rubisco undergoes *N*-methylation via Rubisco LSMT (large subunit *N*-methyltransferase) [50]. Several homologues of protein Lys and Arg NMTs were identified in *E*. *sinica* using query sequences from *A*. *thaliana* (Table 3). High identity to query sequences could imply a conserved function in primary metabolism, as processes such as histone modification are expected to occur in *E*. *sinica* as with all other eukaryotes. Nonetheless, the involvement of an enzyme related to Arg / Lys NMTs in alkaloid metabolism should not be ruled out, as there exists some similarities substrate structure, such as availability of a terminal amino group attached to a flexible side chain. Arg / Lys NMTs are also known to perform successive methylations [49], which may occur in ephedrine alkaloid biosynthesis as well.

## Conclusion

Humanity has long been aware of the pharmacological properties of amphetamine-type alkaloids, firmly establishing plants that accumulate them as traditional sources of medicine. Yet, the widespread use of amphetamine analogues in modern medicine has relied almost exclusively on semi-synthetic, or entirely synthetic production of these compounds [1]. The existing fermentation-based commercial preparation of (1*S*,2*S*)-pseudoephedrine and (1*R*,2*S*)-ephedrine could benefit immensely from the isolation of plant genes involved in the biosynthesis of these substituted amphetamines. The partially mapped ephedrine alkaloid pathway provides a logical basis for the prediction of enzyme types involved in producing these compounds. The establishment of extensive and annotated sequence resources for khat and *E*. *sinica* represents a critical step toward the elucidation of the pathway at a molecular level.

## Supporting Information

S1 Dataset Complete sequences of candidate genes identified in khat (CED-trinity) (see Table 2). (PDF)

S2 Dataset Complete sequences of candidate genes identified in *Ephedra sinica* (ESI-Velvet) (see Table 2). (PDF)

S3 Dataset Complete sequences of queries used to mine khat (CED-trinity) for candidate genes. (PDF)

S4 Dataset Complete sequences of queries used to mine *Ephedra sinica* (ESI-Velvet) for candidate genes. (PDF)

S1 Fig Agilent Bioanalyzer scan results obtained for khat (upper panel) and *Ephedra sinica* (lower panel) total RNA preparations. Data reflect overall length and quantity of RNA molecules prior to preparation for Illumina GA sequencing.
(PDF)

S2 Fig Functional category analysis based on Gene Ontology (GO) annotations of ESI-Velvet unigenes. (PDF)

S3 Fig Phylogenetic analysis of gene candidates. Abbreviations: L-phenylalanine ammonia lyase (PAL) (A), 4-coumaroyl-CoA ligase (4CL) (B), 3-ketoacyl-CoA-thiolase (KAT) (C), cinnamoyl-CoA hydratase-dehydrogenase (CHD) (D), benzoate CoA-ligase (BL) (E), reductase (RED) (F), and *N*-methyltransferase (NMT) (G). Sequences were aligned and analyzed for phylogenetic relationships using the neighbor-joining algorithm. Numbers at each node represent bootstrap values calculated using 1000 iterations. Accession numbers are found in the Materials and Methods, and abbreviations are defined in Tables 2 and 3.
(PDF)
