# Supplementary material for: Transcriptome Profiling of Khat (Catha edulis) and Ephedra sinica Reveals Gene Candidates Potentially Involved in Amphetamine-Type Alkaloid Biosynthesis
Source: PLoS One. 2015 Mar 25;10(3):e0119701. doi: 10.1371/journal.pone.0119701 (PMC4373857; doi:10.1371/journal.pone.0119701)
Supplement: S1 Dataset — (PDF) [file pone.0119701.s001.pdf]

Dataset S1. Complete sequences of candidate genes identified in khat (CED-Trinity) (see Table 2)

>CePAL1-1
MGTEAVIVNGHQNGAFDSLCVKNGDPLNWGSAAESMKGSHIDEVKRMVAEFRKPVVRLG
GETLTISQVAAIGARESGTVEVQLAESARAGVKASSDWVMDSMNKGTDSYGVTTGFGAT
SHRRTKQGGALQKELIRFLNAGVFGNGTESCHTLPNSATKAAMLVRINTLLQGYSGIRF
EILEAITKLINHNITPCLPLRGTITASGDLVPLSYIAGLLTGRPNAKAVGPNGESLNAQ
EAFRKAGISSGFFELQPKEGLALVNGTAVGSGLAAMVLFDANVLAVLSEILSAIFAEVM
QGKPEFTDHLTHKLKHHPGQIEAAAIMEHILDGSSYVKAAKKLHEMDPLQKPKQDRYAL
RTSPQWLGPQIEVIRYATKSIEREINSVNDNPLIDVSRNKALHGGNFQGTPIGVSMDNT
RLAIASIGKLMFAQFSELVNDFYNNGLPSNLTGGRNPSLDYGFKGAEIAMASYCSELQY
LANPVTSHVQSAEQHNQDVNSLGLISSRKTAEAVDILKLMSSTYLVALCQAIDLRHLEE
NLKNTVKNTVNLVAKRVLTTGANGELHPSRFCEKDLLKVVEREHVFAYIDDPCSATYPL
MQKLRQVLVDHALDNGDNEKNVSTSVFQKIGAFEDELKAVLPKEVENARSAIESGNPTI
PNRIKECRSYPLYKFVREELGSAFLTGEKVTSPGEEFDKVFTAICLGKIVDPMLDCLKS
WDGAPLPIC

>CePAL1-2
MDSMNRGTDSYGVTTGFGATSHRRTKQGGALQKELIRFLNAGIFGHNTEACHTLPHMAT
RAAMLVRINTLLQGYSGIRFEILEAITKFLNHNITPCLPLRGTITASGDLVPLSYIAGL
ITGRPNSKAVGPNGESLDPTEAFKLAGIEGGFFELQPKEGLAMVNGTAVGSGLASMVLF
EANVLTILSEVISAIFAEVMQGKPEFTDHLTHKLKHHPGQIEAAAIMEHILDGSAYVKA
AKKVHELDPLQKPKQDRYALRTSPQWLGPQIEVIRAATKMIEREINSVNDNPLIDVSRN
KALHGGNFQGTPIGVSMDNTRLAIASIGKLMFAQFSELVNDFYNNGLPSNLTGGRNPSL
DYGFKGAEIAMASYCSELQYLANPVTSHVQSAEQHNQDVNSLGLISSRKTAEAIDILKV
MSSTFLVALCQAIDLRHLEENLKNTVKNVVSQIAKRVLTVGVNGELHPSRFCEKDLLKV
VDREYVFAYADDPCSITYP

>Ce4CL1-1
MEPQAETQEFIFRSKLPDIHIPNHLPLHSYVFENVSKFSSQPCLINGSTGDIYTYEQVQ
LTARRFASGLDKLGIKQREVIMLLLPNSPEFVLSFLGASFRGAIATAANPFFTPAEIAK
QAKASNAKLIITQACHVEKVKDFAHENDVKLVCIDVAPNGCLHFSELTQPDETELADVD
ILPDDVVALPYSSGTTGLPKGVMLTHKGLVTSVAQQVDGENPNLYFHSEDVILCVLPMF
HIYALNSIMLCGLRVGAAILIMPKFDINALLQLIQKHKVTIAPIVPPIVLAIAKSPETD
KYDLSSIRMLKSGAAPLGKELEDTVRAKFPNARLGQGYGMTEAGPVLAMCLSFAKEPFD
IKPGACGTVVRNAEMKIMDPDTGASLPRNQPGEICIRGDQIMKGYLNDPEATQRTIDKE
GWLHTGDMGFIDDDDELFIVDRLKELIKYKGFQVAPAELEAMLLAHPDISDAAVVGMKD
ESAGEVPVAYVVKSKNSQPTEDEIKLYISKQVVFYKRISRVFFIDAIPKAPSGKILRKD
LRARLTNVKN

>Ce4CL1-2
MEPKNDQEIIFRSKLPDIHIPNHLPLHTYCFEKISEFKDNPCIIDGRNDKIYSYADVEL
ASRKVASGLSKLGIKQGDVIMLLLQNCPEFVFAFFGASYLGAMSTTANPFYTPAEVAKQ
AKASECKVIITQSAYADKVKDLGLKVVTIDEATSVENCLHFSELLTAEDGEIPTVEIKP
DDVVALPYSSGTTGLPKGVMLTHKGLVTSVAQQVDGENPNLYFHENDVILCVLPLFHIY
SLNSVLLCALRVGAAILIMQKFEIVTLMELVQKYKVTIAPFVPPIVLAIAKNGEVDKYD
LSSIRTVLSGAAPMGKELEDSVRAKMPNAKLGQGYGMTEAGPVLSMCLSFAKEPFEIKS
GACGTVVRNAEMKIVDPETGASLPRNKAGEICIRGSQIMKGYLNDPEATAITIDKEGWL

HTGDIGYIDDDDELFIVDRLKELIKYKGFQVAPAELEAMLIAHPNISDAAVVPMKDEAA
GEVPVAFVVRSNGSKITEDEIKQYISKQVVFYKRINRVFFADSIPKAPSGKILRKDLRA
KLAEGIPS

>Ce4CL1-3
MISIETQNPEISSTISPPPPTSTEKTHVFTSKLPDIPIPNHLPLHTYCFQNLSEISEFS
EKPCLIIGSTGKTFTFGETHLISSNVGSGLSNLGIKKGDVVMILLQNSAEFVFTFMGAS
MIGAIITTVNPFYTSNEIFKQLNASNAKLIITQSQYVDKLRENPKFGEEFKVITIDDPP
ENCLHFSILSESKHETEINPDVEFDSQDPVALPFSSGTTGLPKGVILTHKSLITSVAQQ
VDGENPNMYMRPGDVVLCVLPMFHIYSMISVLLVSIRAGAAVLLMQKFEIGALLELIQR
HKVSVAAVVPPLVLALAKNPMVAEFDLSSIRIVMSGAAPLGKELELALQERLPQAIFGQ
GYGMTEAGPVISMSLGFAKQPFPTKYGSCGTVVRNAELKVVDPETGCSLDYNQPGEICV
RGSQIMKGYLNNPEATSSTIDVEGWLHTGDIGYVDDDDEIFIIDRVKEIIKFKGFQVAP
AELEALLVSHPSIADAAVVPQNDEVAGEVPVAFVVRSKGFELTEEDVKGFISKQVVFYK
KLHKVFFVQAIPKSPSGKILRKELRAKLATASPTS

>CeBDH1-1
MYDNNVKIVTFPEFLMRKMKSAMPLDSKRYHWYNPTSVDELQSLLKTVEEANYGARIKL
VVGDTGKGYYKEIEFHDIYIDLRHLPELSVIQRHQNEIEIGAAVTISKAIEALKEEYNA
EVHSQGETVFKKVAAHMEKIASRFLRNSASVGGNLVLAQRKHFPSDIATVLLAVDSLVK
IKTGTGIEKLSLEEFLGWPPLDSESVLLSVTVPNRNLVNNSTSETDTNLVFETYRAAPR
PLGNALPYLNAAFLAEMSHSKSANGIMLNNCRLAFGAYGTKHAIRARTVEEYLTGKKLT
VGVLYEALMLTNATVEPEDGTSSPAYRTGLATGFLFSFLSPLTETSTNSYNGWLDGYDI
PSLSEAKQNHEHLGLMKPQTLLSSGKQVFELGEEYRPVGEPITKSGAAIQASGEAVYVD
DIPAPPNCLYGAFIYSTKPLARVKGIEFKSKSIPNGVIGLISFKDIPETGENIGSEFIF
GSEPLFADEITRCAGQTIAFVVADTQKHADMAAKLAVVHYDTEDLEPPILSVEDAVKRS
SFFEILPFIYPKQVGDLSKGMAEADYKIFSAKMSIGSQYYFYMENQTALAIPDEENCMV
VYSSSQCPEYAGSTIARCLGIPEHNVRVITRRVGGGFGGKAIRAMPVSTACAVAAHKLQ
QPVRTYLNRKIDMIMAGGRHPMEITYSVGFKSDGKITALQLDILVNAGISADISPMMPR
NIVCTLKKYNWGALSFDIKVCKTNHYSKSAMRAPGEVQGSFIAEAVIEHVASTLSMDVD
SVRKMNLHTYETLNVFYENSSGELKEYTLSSIWDRLATSSSFEQRTKSVKEFNRHNIWR
KRGISRVPIVHEVILRSTPGKVSILSDGSVVVEVGGVEIGQGLWTKVKQMTAYALGLIK
CDGSGDLLEKIRVIQSDTLSLIQGGFTAGSTTSEASCEAVRLCCKVLVERLNPLKESLQ
EQMGSINWETLIVQATLQSVNLSASSYFVPELTSKAYLNYGAAVTEVEVNILTGESTIL
RTDIIYDCGKSLNPAVDLGQIEGAFVQGVGFFMLEEYLTNEDGLVVVEGPFTYEIPTID
TIPRQFNVEILNSGHHQKRVLSSKASGEPPLLLASSVHCATRAAIREARRQLCSWKGIE
GSDSAFQLPIPATMPVVKELCGLDMVESYLQWKMTRNSMS

>CeBDH2-1
MAARRISSLLSRSFSASMFPSLGRNPSRVRSIYRFSTAAMPEELIVPPVQVSHTQHLIN
GQFVDAASGRTFPAYDPRTAEVIAHVAEGDAEDINRAVAAARKAFDEGPWPKMSAYERS
RIMLRFADLVEKHNDELAALETWNNGKPYEQSSMAEIPMLARLFRYYAGWADKIHGLTV
PADGNHHVQTLHEPVGVAGQIIPWNFPLIMFAWKVGPALACGNTIVLKTAEQTPLTALY
AGKLFLEAGLPPGVLNIVSGYGPTAGAPLASHMDVDKIAFTGSTETGKVVLELAAKSNL
KPVTLELGGKSPFIVCEDADVDKAVELAHFALFFNQGQCCCAGSRTYVHERVYDEFLEK
SKARALRRIVGDPFKKGVEQGPQIDSEQFEKVLRYIRSGIEGNATLECGGERFGSRGYF
IQPTVFSNVQDDMLIAKDEIFGPVQSILKFKDLGEVIKRANTTRYGLAAGIFTKNLDTA
NTLSRALRVGSVWINCFDVFDAAIPFGGYKMSGIGREKGIYSLHNYLQVKAVVTPLRNP

AWL

>CeBDH2-2
MSAYERQRILLRFADLVEKHNDEIAALETWDNGKPYEQAAKIEVPMVARLIRYYAGWAD
KIHGLTVPADGSYHVQTLHEPIGVAGQIIPWNFPILMFAWKVGPALACGNTIVLKTAEQ
TPLSALYVSKLFHEAGLPEGVLNVISGFGPTAGAALASHMEVDKLAFTGSTVIGQKVLE
LAAKSNLKPVTLELGGKSPFIVCEDADVNKAVELAHFALFFNQGQCCCAGSRTFVHERV
YDEFIEKAKALAIKRAVGDPFKQGIEQGPQIDSEQFEKILKYIRSGIEHGATLETGGER
FGNKGYYVKPTVFSNVKDDMLIAKDEIFGPVQSILKFKDLNEVIRRANASSYGLAAGVF
TQNIDTANTLMRALRVGTVWINCFDTFDAAIPFGGYKMSGIGREKGIYSLSNYLQVKAV
VTPLTNPTWL

>CeBDH2-3
MGSQSSESFVKIPEIKFKKLFINGEFVNSVSGKTFETTDPRTGEVIARIAEGDKDDIDL
AVKAARHAFDHGPWPRFPGSERGRIMMKFAEIIEEHIEELAALDTIDAGKLFSWGKVVD
IPAAARFLRYYAGAADKIRGEVLKMSRELHGYTLREPIGVVGHIIPWNFPTTMFFMKVS
PALAAGCTIVLKPAEQTPLSALYYAHLAKKAGIPDGVLNVVTGYGSTAGAAITSHMDID
KVCFTGSTEVGRKVMQAAATSNLKQVSLELGGKSPLLIFDDADIDTATDLALRGALYNK
GEVCVASSRVYVQEGIYDEFVNKVVEKAKNWVVGDPFDPRSQQGPQVDKNQFEKILSYI
EHGKREGATLLAGGKPLGSKGYYIEPTIFADVKEDMLIGKDEIFGPVMSLMKFKTMEDG
IKSANNTKYGLAAGIVTKDLNVANTVSRSIRAGIIWINCYFGFDTDCPYGGYKMSGFGR
DFGLEALNKYLQVKSVVTPIFNSPWL

>CeKAT1-1
MEKAIKRQQVLLDHLRPFSSSSSNSYEAALSASACLAGDSAAYQRTSAYGDDVVIVAAY
RTALCKSKRGGFKDTFPDDLLAPVLRALIEKTNVNPSEVGDIVVGTVLAPGSQRASECR
MAAFYAGFPETVPVRTVNRQCSSGLQAVADVAAAIKAGFYDIGIGAGLESMTVNPMAWD
GSINPKVKTLEQAQNCLLPMGVTSENVAHRFSVTRQEQDQAAVESHRKAAAATASGKFK
DEIVPVATKIVNPKTGEEKPVTISVDDGIRPNASLSELSKLKPVFKKDGTTTAGNSSQV
SDGAGAVLLMRRSVAMQKGLPILGVFRTFSAVGVDPAIMGVGPAVAIPAAVKAAGLELD
DIDLFEINEAFASQFTYCRKKLGLDPEKINVNGGAMAIGHPLGATGARCVATLLHEMKR
RGKDCRFGVISMCIGTGMGAAAVFERGDAADDLCNARKVESNSLLSKDAR

>CeKAT1-2
MEKALNRQRVLLQHLKPTSLTYESTDFSASICAGYHRTSAFDDDVVIVAAYRTAICKSK
RGGFKDTPPEDLLAPVLKALIEKTNLNPSEVGDIVVGTVLAPGSQRATECRMAAFYAGF
PETVPIRTVNRQCSSGLQAVADVAACIKAGFYDIGIGAGLECMTLNQIDGIQKVNPKVH
DFVQARDCLLPMGITSENVAQRYGVTREEQDQAAVESHKRAASAISSGKFRDEIIPVST
KIVDPRTGEESPITVLVDDGIRPNTNMKDLAKLKPAFKKDGSTTAGNASQVSDGAGAVL
LMRRSLAIQKGLPILGVFRSFVAVGVDPAVMGVGPAAAIPVAVKAAGLELDDIDIFEIN
EAFASQFVYCCKKLELNPDNVNVNGGAIALGHPLGATGARCVATILNEMKRRGKDCRFG
VISMCIGSGMGAAAVFERGDCVDVLCNARPVERNSLLSMDAR

>CeCHD1-1
MAEIRVNMEVGRDGVAVIEICNPPVNALAIPILAGLKEKFDEATRRGDVRAIVLTGKGG
RFSGGFDINVFLKIHKTGDVSLMPDVSVDLVVNTIEDCKKPVVAAVEGLALGGGLELAM
GCHARVAAPKTQLGLPELTLGVIPGFGGTQRLPRLVGLSKAIQMMLLSKSIMSEEGNKL
GLIDAIVSTEELLKVSRQWAIDIAEMRRPWMRSLHRTDKLGSLAEAREILKTARQQAKK

TAPNMPQHFGCLDVIEDGIVHGGYTGVLKEAKVFKDLILSETSKGLVHVFFAQRATSKV
PHVTDIGLKPRQVKKVAVIGGGLMGSGISTALILSNIRVVLKEVNSEYLLKGIKLIEAN
VRSLVAKGKLTQDKAERALSMLKGVLDYSEFKDEDMVIEAIIENIPLKQKIFTEIEQEC
PSHCILATNTSTIDLNLIGQKTSSQDRIIGAHFFSPAHIMPLLEIVRTEKTSAQVIVDL
MAVGKLIKKVPVVVGNCTGFAVNRTFFPYSQSAHLLVHLGVDPFRIDRVISSFGLPMGP
FTLQDLAGYGVAIAVGKIFADAFPDRTFHTPLVELLIKNGRNGKNNGKGYYIYEKGSKP
KPDPSVVPIIEESRLLANLMPGGKPISVTDQEILEMILFSVVNEACRVLDEGVVVRASD
LDIASILGMSFPSYRGGIVFWADLVGAHHVFTSLKKWSQLYGNFFKPSRYLEERAKRGM
LLSDPSPSTASRSRL

>CeCHD1-2
MANRSKGRTIIEVGSDGVAVITIIHPPVNALSFDVLNSLKENYDQALRRDDVKAIVVTG
ANGKFSGGFDITSFGGIKEGKSTEPKPGFISVEILSDTMEGARKPSVAAIDGLALGGGL
EVAMACHARISTPTAQLGLPELQLGIIPGFGGTQRLPRLVGISKALEMMLMSKPVKGAE
AHNLGLVDALVSPNELVDTARRWALDILERRKPWVASLYKNDKLDSLGDAREIFKFAKA
QARKQAPNLTHPLVCIDVVEEGIVSGPRAGLWKEAEAFQELVRSDTSKSLIHIFFAQRG
TTKVPGVTDWGLVPRRVKKVAILGGGLMGSGIATALVLSNYPVILKEVNEKFLEAGIGR
VRANLQSRVKKGSMSQEKFEKTLSLLKGALDYEGFKDVDMVIEAVIENVSLKQQIFADL
EKYCPPYCILASNTSTIDLNLIGERTNSQDRIIGAHFFSPAHVMPLLEIVRTNHTSSQA
IVDLLDIGKKIRKTPVVVGNCTGFAVNRVFFPYTQAAILLVERGTDVYQIDKAITKFGM
PMGPFRLIDLVGFGVGIATGLQFVQNFPERTYKSMLLPIMQEDKRLGEATRKGFYLYDD
KRKANPDPELKKYIEKARSMSGATVDPKLVTLPEKDIVEMIFFPVVNEACRVFAEGIVV
KASDLDIASVMGMGFPPYRGGILFWADSLGSKYIYSRLEEWSRMYGEFFKPCAFLAERA
AMGAPLSAPVEQAKSRM

>CeBL1-1
MENLPKCEANYTPLTPITFLKRAAMAYANRTSVIHESTFFTWSETYRRCRCLASSLISL
NIAKNNVVSVLAPNIPAMYEMHFAVPMAGAVLNTINTRLDAKNIATILRHSEAKIFFID
YQFVPVAREAIGILMAESARIPAVVVIDDINSPTGLRLGVLEYEQLINNGDPNFVPLEI
EDEWDPIALNYTSGTTSDPKGVVYSHRGAYLSTLSLILGWEMATQAVYLWSLPMFHCNG
WTFTWGVAARGGTNVCIRNTTASDMYRNISLHKVTHMCCAPIVFNILLDAKPEERREIT
SPIQILTGGAPPPSSLLQKIEQLGFHVTHAYGLTEATGPALVCEWQAKWNRLPMEEQAK
IKARQGLSVLTLSDVDVKDADTMVSVPRDGKTIGEIVLKGSSIMKGYLKDEEATAKAFK
NGWFFTGDVGVIHPDGYLEIKDRSKDVIISGGENISSVELENVLYKHPRVMEAAVVAMP
HPLWGESPCAFIAVKKNPMSGKTDDVTEGEIVAYCRKNLPSFMVPKKVEFMAELPKTST
NKIQKFQLRALAKTFVVSDDKKISPQPHQHRRNLPLSRL

>CeBL1-2
MEGAIRCSANYVPLTPISFLERSAIVYRDRVSVVYRDVKYTWRETHQRCIRLASALDQL
GISPGDVVAALAPNIPALYELHFGVPMAGAVLCTVNIRQDSAMVSVLLKHSEAKIIFVD
YQFLNIAQGALEILSKTGTRLPLIVLITENDRPASTISTSHAALEYEKMLEMGKLDFEI
RWPKDEWDPISLNYTSGTTSSPKGVIYSHRGAYLNSMAAVLLNEMNSMPVYLWCVPMFH
CNGWCLTWAVAAQGGTNICLRNVNAKDIFSNIVRYKVTHTGGAPTVLNMIINAPNNEKK
PLPGKVSVMTGGAPPPPRVLHSMEELGFVVTHSYGLTETYGPGTVCTWKPEWDSLPREA
KAKLKARQGLHHLGMEELDIKDPVTMKSVPPDAKTLGEVMFRGNTVMNGYLKNLEATQE
AFKGGWFRSGDLGVKHPDGYIELKDRSKDIIISGGENISTIEVEAVLFSHPAVLEAAVV
GRPDDHWGETPCAFVKLKDGCSASPEELIGYCRNRLPHYMAPRTVVFEDLPKTSTGKTQ
KFVLREKAKAMGSISKRTTSKL

>CeBL1-3
MKHHHRLKTNFNRFHLYLLSRFSRRFSHFTGDPHERPESWKSIEGLVRCSANYAPLSPI
GFLERSAKVYRDRTSVVYGSLKYTWSETHERCLKLASSLTHLGISRGDVVATLAPNVPA
IYELHFAVPMAGAVICTLNSRHDSAMLSVLLKHSEAKIIFVDSQLLETACQALHLLENT
GAKLPILVLISESGDSNATSLFKSNSYEYESLLATGDNGFKIRRPESEFDPISVNYTSG
TTSRPKGVVYCYRGAYLNSLSTVFLHGMSSMAVYLWTVPMFHCNGWCLIWGVAAMGGTN
ICLRKVSAKGIFDSISEHSVTHMGGAPTVLNMIVNSPVSDRSPLPHKVEVMTGGAPPPP
QIIHKMEELGFGVSQLYGLTETYGPGTYCVWKPEWDSLPLEERAKLKARQGMQHIGLED
VDVKDPDTMESVPADGKTLGEIMFRGNTVMGGYFKDVKATEEAFRGGWFRSGDLGVKHP
DGYIEMKDRLKDIVISGGENVSTVEVETVLYSHPAVLEAAVVGRPDDHWGQTPCAFVKL
KDGFDDVDAQEFINYCRDRLPHYMAPKTVIFQDLPRTATGKLQKFILREKAKALGSLS*
V*TNHVLTETKAFSSIHY

>CeBL1-4
MEDLKPSLPNTSPLTPLGFLERAATVYGDCPSVIYNNTTYTWSQTHRRCLQVASSLSSL
GIQRGHVVSVIAPNIPAMYELQFAVPMSGAILNNINTRLDARTVYVLLLHSECKLVFVD
CLSRALVLEVISLFPPNIRPPTLVLIDDDDEPSSPAVEFFSTYESLVMNGDPDFKWVRP
KCEWDPFVLNYTSGTTSSPKGVVHCHRGIFIITVDSLIDWGVPKQPVYLWTLPIFHANG
WSYPWGMAAVGGVNICLRKFDASTIYGAIKRYGVTHMCGAPVVLNMLSSSSDIEPLKNP
VQILTAGAPPPAAVLFRTESLGFVVSHGYGLTETAGLVVSCAWKPKWNRLPATERARLK
ARQGVGIVGFTEVDVVDEGSGASVKRDGSTLGEVVLRGGCVMLGYLKDPEGTSKCMKEN
GWFYTGDVGVMHPDGYLEIKDRSKDVIISGGENLSSVEVESVLYTNPAVNEAAVVARPD
EYWGETPCAFVSLKTGLTRKPSEKVIIDYCRARLPHYMVPKTVVFKEELPKTSTGKIQK
FILREIAKAMGGSRLSKM

>CeBL1-5
MEGQKASPANSCPLTPLGFLERAATVYGDCPSLIYNNTTYTWSQTHRRCLQLASSLSSL
GIKRRDVVSVIAPNIPAMYELYFAVPFIGAVLNSINIRLDARTVSVLLLHSESKLVFVD
VMSLSLIRDAISLFPSDRVNRPLLVLIEEEEDYASQINASINIVCDSYEELVSKGDPNF
KWVRPETEWDPITLSYTSGTTSAPKGVVHCHKGVFIVTVTSLVEWSIPSQPVYLWTLPM
FHANGWSFPWGMAAVGGTNICLRKVDPSTVYGLINKHRVTHMCAAPVVLNLLSNSPNTK
LLNPVNILTGGAPPAAAILTRIESLGFVVTHGYGLTETGGVVVSCAWKPQWNRFPATER
AKLKARQGVRSIGLVEVDVLDPDTGGSVKPDGSTIGEIVLRGGGIMLGYLKDPEGTSKC
MRNGWFYTGDVGVMYPDGYLEIKDRSKDVIITGGENLSSVEVESVLYTNPAVNEAAVVA
RPDEFWGETPCAFVSFKAGIDWKPSEREIIEYCRKKLPHYMVPKTVVVEEELPKTATGK
IQKNLLRDMARALGPSRVSRM

>CeBL1-6
MRDIDDLPQNAANYTALTPLWFLERAATVHPERKSVVHGSVNYTWRQTYERCRRLASAL
SKHNIGVGCTVAVIAPNIPASYEAHFGVPMSGAVLNPVNIRLNAPTIAFLLGHSSSAIV
IVDQEFFSLAEEALKIMAEKSGSKFEPPLMVVVADESCDPKSLKYALGKGATEYEKFLE
TGDPAFAWEPPQDEWQSIALGYTSGTTASPKGVVLSHRGAYLMSLSGALIWGMNEGAIY
LWTLPMFHCNGWCFTWTLAALCGTNICLRQVTAKAVYSAIVNDGVTHFSAAPIVLNTIV
NAPKEDTILPLPRIVHVTTAGAAPPPSVLFAMSERGFRVTHTYGLSETYGPSTVCAWKP
EWNSLPPIDQARLNARQGVRYIGLEGLDVVDPKTMKPVPADGNTLGEIVMRGNLVMKGY
LKNPKANEESFANGWFHSGDLAVRHPDSYIEIKDRSKDIIISGGENISSLEVENMLYMH
PAVFEASVVARPDERWGESPCAFVTLKPDVDKADEGRLAEDIMKFCRSKMPAFWVPKSV

VFGPLPKTATGKIQKHVLRAKAKDMGTAKISKL

>CeThDPC1-1
MAAASSTTLFSSPYPFHYYNNSSTKPCLPILRFGTVNHGNTSLSRPLHVTNSIPKAAAA
ADTTTASPTDSPPRFAPDEPRKGADILVEALERQGVTNVFAYPGGASMEIHQALTRSNI
IRNVLPRHEQGGIFAAEGYARSSGLPGVCIATSGPGATNLVSGLADASLDSVPIVAITG
QVPRRMIGTDAFQETPIVEVTRSITKHNYLVLDVDEIPRVVKEAFFLATSGRPGPVLID
IPKDIQQQLACPNWNQPIKLNGYISRLPKSPNEAHLEQIVRLISEAKKPVLYVGGGCSN
SGDELRQFVELTGIPVTNTLMGLGTYPASDDLSLQMLGMHGTVYANYAVDKSDLLLAFG
VRFDDRVTGKLEAFASRAKIVHIDIDSAEIGKNKQPHISVCGDVKLALKGMNRVLESKG
SKLGLDFSAWREELDEQKLKYPLSYKTFGDAIPPQYAIQVLDELTGGNAIISTGVGQHQ
MWAAQYYKYKRPRQWLTSGGLGAMGFGLPAAIGAAVANPDAVVVDIDGDGSFMMNVQEL
ATIRVENLPVKILLLNNQHLGMVVQWEDRFYKANRAHTYLGNPEKETEIFPNMLKFAEG
CGIPAARVTRKDDLRAAIQTMLDTPGPFLLDVIVPHQEHVLPMIPSGGGFDDTITEGDG
RIKKGDGTTKK

>CeThDPC2-1
MDTKIGSLDTCKAESNNVGCPSNGGVSSIQSSVPATFVNSAESTLGRHLARRLVQVGVT
DVFSVPGDFNLTLLDHLIAEAGLNLVGCCNELNAGYAADGYARSRGVGACVVTFTVGGL
SVLNAIAGAYSENLPVICVVGGPNSNDYGTNRILHHTIGLPDFSQELRCFQTVTCYQAV
VNNLEDAHELIDTAISTALKESKPVYISVSCNLPAIPHPTFSREPVPFSLTPRLSNRMG
LEAAVEATAEFLNKAVKPVMVAGPKMRSAKACDAFVELADACGYALAVMPSAKGLVPEH
HPHFIGTYWGAVSTAFCAEIVESADAYLFAGPIFNDYSSVGYSLLLKKEKAIIVQPDRV
TIANGPAFGCVLMKDFLKALAKKLKRNTTAYENYHRIYVPEGHPLKSGPKEPLRVNVLF
EHIQKMLSHETAVIAETGDSWFNCQKLKLPRGCGYEFQMQYGSIGWSVGATLGYAQAVP
EKRVIACIGDGSFQVTAQDVSTMLRCEQRSIIFLINNGGYTIEVEIHDGPYNVIKNWNY
TGLVDAIHNGEGKCWTAKVLCEEDLIEAIETATGPKKDCLCFIEVIVHKDDTSKELLEW
GSRVSAANSRAPNPQ

>CeThDPC2-2
MEAGNQIGSVSKPSSAPAPVRGGTCSGTLGRHLARRLVEIGVRDVFSVPGDFNLALLDH
LIAEPELNLIGCCNELNAGYAADGYARAKGVGACVVTFTVGGLSVINAIAGAYSENLPL
ICIVGGPNSNDYGTNRILHHTIGLPDFSQELRCFQTVTCTQAVVNNLDDAQELIDTAIS
TALKESKPVYISVSCNLPGIPHPTFTRDPVPFSLAPKVSNQLGLEAAVEATAEFLNKAV
KPVLVAGPKLRVAKAQKAFVELADESGYPIAVMPSGKGLVPEQHPHFIGTYWGAVSTSF
CGEIVESADAYVFVGPIFNDYSSVGYSLLIKKEKAVMVQPNRITIGDGPSFGWVFMADF
LHALAKKLKKNNTAMENHRRIFVPPGMPLKCGNDEPLRVNVLFKHIQEMLTGDTAVIAE
TGDSWFNCQKLRLPENCGYEFQMQYGSIGWSVGATLGYAQAAKDKRVIACIGDGSFQVT
AQDISTMIRCGQRSIIFLINNGGYTIEVEIHDGPYNVIKNWNYTGLVDAIHNGEGKCWT
VKVKTEDELREAIATATGEQKDSLSFIEVLVHKDDTSKELLEWGSRVSAANSRPPNPQ

>CeTA1-1
MAVSLRSSTSISRASVGHRFPTPNFASDSSGSVSFPFLPHNLNLSLKSREITRISAMAK
AESRIEEIGVDISLSPRVNSVKPSKTVAITDQATALVQAGVPVIRLAAGEPDFDTPTPI
AEAGINAIREGYTRYTPNSGTQELRAAICHKLKEENGLTYTPDQILVSNGAKQSILQAL
LAVCSPGDEVIIPAPFWVSYPEMARLADATPVILPTQISENFLLDPKLLRSKISDKSRV
LILCSPSNPTGSVYPKELLQEIAEIVAKHPRLLVLSDEIYEHIIYSPATHTSFASLPGM
WERTLTVNGFSKAFAMTGWRLGYLAGPKHFVSACNKIQSQFTSGASSISQKAAVAALGL

GYAGGEAVSNMVKAFRERRDFLIKSFGDMQGVKMSEPQGAFYLFIDVSSYYGTKAEGFG
AITNSESLCRYLLDEAQVALVPGDAFGDDSCIRISYAASLTTLQAAVERIKKALASLRS
AVPV

>CeTA1-2
MQSQCTWTSSQMLCPLSFKPTLFAFSKHLRTTSSHRIWTRGSSSRVANYPSFMATLSPV
STEKDAVSSQNDSAQEPQKPLQVSKRLEKFKTTIFTQMSSLAIKHGAINLGQGFPNFDG
PEFVKEAAIQAIKDGKNQYARGYGVPEFNSTIAARFKKDTGLVVDPEKEITVTSGCTEA
IAATMLGLINPGDEVILFAPFYDSYEATLSMAGAKIKSITLRPPDFAVPINELKSAITK
NTRAILINSPHNPTGKMFTREELNTIASLCIENDVLVFTDEVYEKLAFEMEHISMASLP
GMYERTVTMNSLGKTFSLTGWKIGWAIAPSHLTWGVRQAHAFLTFATSTPMQYAAATAL
DAPDSYYVELKRDYMAKKEILVEGLKTVGFKVFPSSGTYFVVVDHTPFGLDNDIAFCEY
LIKEVGVVAIPTSVFYLNPEDGKNLVRFTFCKDEKTLRDAVERMKEKLRRR

>CeTA1-3
MSTVIVTEDEERPSHSANYLSGKLSKVARTFTPSPIQELSHLAQRSNAINLAEGFPDFP
APPHIKNAAVSAINSDFNQYRHVQGICDHLSRKMKDEHGLIVDPSTDIAICCGQTEAFA
AAVFSIIDTGDEVVLFDPCYETYEGCITMAGGVPVYVALDPPHWTLDPDRFLKSFTSKT
KAVVLNSPHNPTGKVFTKDELEIIAGACCTRDCLAITDEVYEHIAFDDEKHISLASLPN
MQEKTIITSSISKTFSVTGWRIGWAIAPAVIASAIRNIHVKVTDSAPAPFQEAALTALT
SPPEFYESLRREYESKRNYIVEFLGEVGFQIEFKPKGSFFLFAKLPDQCALSDVEYVRE
LINKAGVVAVPGCAFFHTEVSCEKPSQEGEDRSYQGRYIRFAFCKSNDTLTAAAEKLRT
WFYRTKQYLGLH

>CeTA2-1
MESNGSLLTNPETEMASRITIKGILSLLMQSVDEKCEKRVISLGIGDPSAYSCFKTTHA
AEDAVSDSLHSQKFNGYSPTVGLLQTRRAVAEYLSRDLPYKLSPDDVFVTSGCTQAIDV
SLAMLARPGANILLPRPGFPIYELCASFRNLEVRHFDLLPEKGWEVDLDAIEALANQNT
VAIVIINPGNPCGNVYSYQHLKKIAETASKLKTLVIADEVYGHLAFGQNPFVPMGVFGS
IVPVLTLGSLSKRWIVPGWRLGWFVTSDPSGMFREPKTIERIKKYFDILGGPATFIQAA
VPQIIEQTNAVFFEKTIDLLKQTSDICCVMIKEIHCITCPHKPEGSMAFMVKLNLSLLE
DISSDIDFCFKLAKEESIIILPGTAVGLKNWLRITFAVDPSSLEEGLKRVQIFCQRHSK
QLKAC

>CeTA2-2
MENGGKKWNFQATQDMTDTSAITVRGILNKLMGNLNKDDDRQVIPLGHGDPSAFPCFRT
AITAEDALVDALRSAKYNCYSPSTGLLPSRRAIADYLNDDLPYKLSPDDVYVTLGCTQA
IEIAITVLSRPGANILLPKPGFPYYEARAAVSHLEARHFNLLPESGWEVDLENVEAIAD
ENTVAMVIINPGNPCGNVYSYEHLKKIAETARRLGILVIADEVYNHLAFGSNPFVPMGT
FGSIAPVLTLGSISKRWIVPGWRLGWLVTSDPNGILQKAGVVDSIKDSLDLTPDSVTFI
QGAIPQILENTKVDFFSKIIGLLREDADTCCDKIEEIPCITCPNKPEGSMFVMIKLNLA
LLEDIDDDMDFCLKLAQEESVIVLPGIVVGLRNWLRVTFAIEPSALEEGLERMKAFYLR
HAKKQAMNSIQT

>CeRED1-1
MADTDLDSREKRWSLNGMTALVTGGTRGIGHAIVEELAGFGVLVHTCSRNQVELDQRLK
EWESKGFKVTGSVCDLLNRDQRTNLMENVSSFFHGKLNILVNNVGRSITKHTLEYTAED
YSFLMTTNFESTYHLCQLAHPLLKASGNGSIVNISSIAGSIATQKSSIYAASKGATNQV

TRNLACEWAKDKIRVNAIAPGLIRTSLIDALIMEDPEAKEFVNNIVTRTPLLRAGEPNE
VSSLVAFLCFPAASYITGQVLLVDGGFTVNGF

>CeRED1-2
MAKSETSFNNSRWSLKGMSALVTGGTRGIGRAVVEELAGLRATVHTCSRNETELKKCLK
EWEGKGFVVTGSVCDLSSRAQREKMMEEVGSVFNGRLNILVNNVGTNIRKQTTEYSAEE
FSFLMATNLESAYHLSQLTYPLIKATGVGSIVFISSVAGLLHVGSGSIYGATKGAINQL
TKNLACEWAKDNIRTNCVAPWYIRTPLVEHLLGKKEFLEKVISRTPLQRVGEPEEVSSL
VAFLCSPAASYITGQVISVDGGFSVNGFSPEMRLD

>CeRED1-3
MAEQEQRGMEQRWSLKGMTALVTGGTRGIGFAIVEELAGFGAKVHTCSRNQKQLNERIQ
EWKNKGFQVSGSVCDLNSKDQREKLTQTVSSVFDGKLNILINNAATTLLKDYTNHTCED
YAMLMSINVESPLHLCQLSYPLLKASGNASIVFISSIGGVIALPRVSVYAATKGAINQM
TKNLACEWAKDKIRTNSVAPWGVKTTISQELDVDPDTIKAYTGAVSRTPISRLAEPCEV
SSLVAFLCLPAASYITGQVICVDGGHTVSGF

>CeRED1-4
MISSNSRASSRNNNRWSLHGMNALVTGGTRGIGFAIVEELVNLGASVHTCARNESELQK
CLSEWNGSGFGISGSVCDVSVRPQREELIDTVSSVFDGKLNILINNVGTNIRKPMVEFT
AEDFSTLMGTNFESAFHLSQLSYPLLKASGVGSVVFTSSVTGFVSLKSMSVHGATKGAI
NQLTKNLACEWAKDNIRSNAVVPWYIKTSMVKQVLSNEEYLEEVFSRTPLRRLGDPSEV
SSLVAFLCLPASSCITGQIICVDGGMSVNGFYPTHD

>CeRED1-5
MLGSHTNRDTHTEIMAGSREDSRWSLQGMTALVTGGTKGLGFAVVEELAKLGASVHTCA
RNEAQLKECLSEWKGKGFQVTGSVCDLASRPERENLMTEVSTLFNGKFNILVNNVGTNI
RKSTVEYTADDFSFMINTNLESAYHMCQLVHPLMKSSGLGSIVFISSVGGVVAVNTGFS
IYAATKGAMNQLAKNLACEWAKDNIRVNSVAPWFILTELAKPYLDDEKFGAAVNSRCPM
GRTGEPKEVSSLVAFLCMPAASYITGQTICVDGGTTVNGFSFP

>CeRED1-6
MAGNSNRWSLQGMTALVTGGSSGIGFAVVEELAGLGATVHTCSYNESQLNECLREWKTK
GFPVTGSVCDLKYRTQREELMASVSSLFNGKLNILINNVGTVLSKPTTEFTADEFSLIM
NTNLESGFHLSQLAYPLLRNSGNGSIVFTASVAGVVSVSVGTPYGLTKGALIQLSKDLA
CEWAKDNIRVNAVAPWFIITDLTKAYFEDEKFNEAVISRTPMGRTAEPKEVASLMAFLC
MPAASYITGQTISMEE

>CeRED1-7
MAGNSIDSRWSLQGMTALVTGGSSGIGLAVVEELAGLGATVHTCSFNESQLNDCLGHWK
SKGFRVTGSFCDLTNRTQREELIATVSSLFNGKLNILINNVGTGMVKPTTEFTAEEFSF
MMSTNVESGFHLSQLAHPLLKNSGAGSIVFTSSVAGVVSLGVGSLYGLTKGALIQLTKN
LACEWAKDIIRVNAVAPWFVKTELTKPIFEDKNLLEAVNSRNPMGRTGEPEEISSVMAF
LCMPAASYITGQTICIDGGLSVNGFSFP

>CeRED2-1
MGSTPLLALGSTGRSIPQLGYGTAEFPFGANPEIARESILHAIKIGYRHFDTAAYYKTE
QPLGEAIAEALRLGFIQSRDELFITSKLWCSDAHPNLVIPALRKSLGNIGVDYLDLYLV

HFPVSMKPGPYEYPLKDTFPMDFKAVWEDMEECQKLGLTKSIGVSNFSYKKLESLLATA
KIPPSVNQVEMNSKWQQKKLRELCDRNNILIVAYSPLGAKGTPWQGNLVTECEVLKDIA
AANGRTVAQVCLRWLCQQGVGIVVKSFNKERIEENFDIFSWELKQEEVDKIGETPQHRG
YLAQGFISDDAPYKSVEEFWDGEL

>CeRED2-2
MLSIPVAPLDSTGKTTIPLVGFGTAEYPFGSSQSVKQYILQAIEIGYRHFDTASVYQSE
VPLGEAVAEALRLGLIKSRNDLFITSKLWCSDACRDPPCTTKIPPVYNIHFKINLTINV
FCQSC*IIEFNFCRNLGMEYLDLYLIHFPVRLMEGTPSTFKKKDIFEMDLVSVWEAMEE
CQNLGLTKSIGVSNFTCKKLERLLANARIPPAVNQVSLSLLPLISSVCEKGRSL*C*NV
NFGQKLCVDKVEMHPVWQQKKLRQFCDEKGIHITAYSPLGAKGTPWAISGLMDFEVLKE
IANARGKTVAQVCLRWVYEQGVSLVVKSFNKERMKQNLDIFDWKLSEDDLKKIDQIPQH
QGSSAEIFVSDEGPYKSIAELWDGEI

>CeRED2-3
MEGDQIPAVILNSGHKMPSIGMGTVTLPLPPTDTLASIFIDAIETGYRHFDTAAVYGSE
EAVGLAVRQAVERGLVKSREEIFITSKLWCEDAHPGLVIPALKRTLERLGMDYVDLYLI
HWPARLKQGSPPFSFQKEDMLPFDIIGTWKDVEECSRLGLAKSIGVSNCGHKKLTQILE
QATIPPANNQVEMNVACQQHNLLKFCKEKGICLSAWSPLRAVMETPILREIADAKHKTV
AQIALRWVYQQGAIPISKSFNKQRMKENLQILDFELSQDEIEKIKQVPHKRMVSGDSWI
HEKYGNYRSLEELWDGDV

>CeRED2-4
MERKDNSNGPLYFDLNTGGKIPSIGLGTWKAPPGVVGEAVIDAVNAGYRHIDCASVYGN
EKEVGEALKVLFSTGVVKRNEMFITSKLWCSDQAPEDVSKALTKSLEDLQLDYIDLYLI
HWPFRTKLGLSGWDPEIMAPLCLPETWTAMEGLYASGQARAIGVSNFSTKKLQDLLTYA
KVPPAVNQVECHPVWQQPGLHNLCKSTGVHLAAYSPLGSPGTWIKGEILKEPILIEIAE
KLNKSPAQVALRWGIQSGHSVLPKSTNETRIKENISLLDWCIPPELFAKFSYIHQRRLL
RGDFAIHETCSPYKSLQELWDGEI

>CeRED2-5
MGSLDIGTEIRCFELNTGARIPSVGLGTWQADPGVVGEAVISAVKLGYRHIDCAHVYGN
EKEIGDALKKLFDDGVVRRQDIWITSKLWCRDHAPEDVPKALETTLRNLHLDYLDLYLI
HWPCAYKRGSLENKPENLTKTDIPSTWRAMEELYYSGKARAIGVSNFSTKKLEDLLDVA
RVPPAINQVECHPQWQQRKLHDFCKINGIHVTAYSPLGSPGNEFMKGDVVLENPIICSI
AKKLGKSPAQVTLRWGLQMGHSVLPKSTVDAWTKENLDVLDWSIPQDMISEFSQIKQEK
LLKGGEFLHETLSGYTTLKELWDGEI

>CeRED2-6
MAITLNNGFKMPVVGLGVWRMEGKDIRDLILNAIKIGYRHFDCAADYKNEAEVGEALAE
AFQTGLVKREDLFITTKLWNSDHGHVLEACKDSLKNLQLDYLDLYLVHFPIATKHTGVG
TTDSALDKDGVLDIDTTTSLETTWHAMEDLVSKGLARSIGISNYDIFLTRDCLAYSKVK
PAMNQIETHPYFQRDSLVKFCQKHGICVTAHTPLGGAVANTELFGSVSCLDDPVLKGLA
EKYNKSAAQIVLRWGIQRNTVVIPKSSKLERLKENYQVFDFELSKEDMDLIKGVDRKYR
TNQPAKFWGMDLYA

>CeRED3-1
MADISPSTVLVTGAGGRTGQIVYKKLKERSEHYVARGLVRTQESKEKIGGADDVFIGDI

RDAGGIVPAIEGIDSLIILTSAVPKMKPDFDPSKGGRPEFYFEDGAFPEQVDWIGQKNQ
IDAAKAAGVKQIVLVGSMGGTNPNHPLNSLGNGNILVWKRKAEQYLADSGIPYTIIRAG
GLQDKDGGVRELIVGKDDELLQTETRTIARADVAEVCIQALQFEEAKFKAFDLASKPEG
TGTPTKDFKSLFSQITTRF

>CeSMR3-2
MASRLAPSPATLLCSHLPVELPKQPSCITLFALPSLTVPWRVNVSSSRRQLLSSINAVQ
EELTESPNSETTDDYDSRTSSSSTSKLVLVVGASGGVGQLVVASLISRNIKARLLLRDP
DKATTLFGMQDKDNLEVFKGDTRCPEDLDPSIFEGVTHVICCTGTTAFPSRRWDGDNTP
ERVDWEGVRNLVSALPSSLTRIVLVSSVGVTKYDALPWSIMNLFGVLKYKKMGEDFVRN
SGLPFTIIRAGRLTDGPYTSYDLNTLLKATAGQRRAVLIGKGDKLVGEVSRLVVAEACI
QALDIEFTQGEVYEINSIEGDGPGSDSQKWQELFKNAQSL
