# Supplementary material for: Transcriptome Profiling of Khat (Catha edulis) and Ephedra sinica Reveals Gene Candidates Potentially Involved in Amphetamine-Type Alkaloid Biosynthesis
Source: PLoS One. 2015 Mar 25;10(3):e0119701. doi: 10.1371/journal.pone.0119701 (PMC4373857; doi:10.1371/journal.pone.0119701)
Supplement: S2 Dataset — (PDF) [file pone.0119701.s002.pdf]

Dataset S2. Complete sequences of candidate genes identified in *Ephedra sinica* (ESI-Velvet) (see Table 2).

>EsPAL1-1
MVAGAEMAQTAFVQHVKDGGIREFLCKGSDSSNDPLNWVAAAKSMSGSHFDMVRDMVEV
YLNAKEVSIEGKTLTVAQVTAVARKAEKTAIKLDAEAAKERVEKSANWVLTQMNKGTDT
YGVTTGFGATSHRRTNQGAKLQKELIRFLNAGVLGCDDNVLPVETTRAAMLVRTNTLMQ
GYSGIRWEILAAVENLLNAGLTPKLPLRGTISASGDLVPLSYMAGLLTGRPNCKVNTRD
GTVLSGSEALKQIGIEKPFELQPKEGLAIVNGTAVCAALASLVCFDANVLALLSEVMAA
MFCEVMNGKPEFADPLIHRLKHHPGQMEAAAIMEYVLDGSSYMKHAVEIHERNPLQKPK
QDRYALRTSPQWLGPQIEVIRAATHMIEREINSVSDNPMIDVARDKALHGGNFQGTPIG
VSMDNLRLAVAAIGKLMFAQMSELVNDYYNGGLPSNLSGGPNPSLDYGFKGAEIAMASY
CSELQYLANPVTNHVESAEQHNQDVNSLGMVSARKSEEALEILRLMLSTYLTAICQAID
LRHLEENMLATVKQTVAQVARKTLSTGANGELLPGRFCEKELLQVVENEHVLSYIDDPC
SPNYVLMQKLRGVLVEHALKNREAEKDLRTSIFHRIEEFETELKVQLESQATTVRANFD
NGVTALPNRIKDCRSYPLYSFVRETLGTQLLSGDRDISPGEDIEVVYKAVKANDIIVPL
FKCLDGWKGTPGPF

>EsPAL1-2
MDQIEAMLCGGGEKTKVAVTTKTLADPLNWGLAADQMKGSHLDEVKRMVAEFRRPVVNL
GGETLTIGQVAAISTVGGSVKVELSETSRAGVNASSDWVMESMNKGTDSYGVTTGFGAT
SHRRTKNGVAFQTELIRFFNAGIFGSTKETCHTLPHSATRAAMLVRVNTLLQGYSGIRF
EILEAITSLLNHNISPSLPLRGTITASGDLVPLSYIAGLLTGRPNSKATGPDGESLTAK
EAFEKAGISTGFFDLQPKEGLALVNGTAVGSGMASMVLFEANVQAVLAEVLSAIFAEVM
SGKPEFTDHLTHRLKHHPGQIEAAAIMEHILDGSSYMKLAQKVHEMDPLQKPKQDRYAL
RTSPQWLGPQIEVIRQATKSIEREINSVNDNPLIDVSRNKAIHGGNFQGTPIGVSMDNT
RLAIAAIGKLMFAQFSELVNDFYNNGFPSNLTASSNPSLDYGFKGAEIAMASYCSELQY
LANPVTSHVQSAEQHNQDVNSFGLISSRKTSEAVDILKLMSTTFLVGICQAVDLRHLEE
NLRQTVKNTVSQVAKKVFTTGVNGEFHPSRFCEKDLLKVVDREQVFTYVDDPCSATYPL
IQRLRQVIVDHALFNGESEKNAVTSIFHKIGAFEEELKAVLPKEVEAARAAYGNGTSAI
PNRIKECRSYPLYRFVREELGTEFLTGEKVVSPGEEFDKVFTAICEGKLIDPLMDCLKE
WNGAPIPIC

>Es4CL1-1
MKAAVEGEYLYRSKLPDIDIPDNLPVHDYCFQHIEKFANNPCLIDGATERVWTYAEVEL
NSRKVAAGLTASGVKSGDVVMLLVQNCAEFAFTFLGASMIGAIVTTANPFYTPGEIAKQ
AAGSNARIVVTHAAFTAKLQGLDNISAIYTIDGPVPEGCKAFSELLDADEEKDFPKGIR
IDPSDVVALPYSSGTTGLPKGVKLSHRGLVASIAQLADGENPNLYFNSEDVLLCVLPLF
HIYCLHTVLLCALRVGSAMVIMPKFNITSMLQYIDKYKISILPIVPPIVVEITKCDQIV
AEKVSSVRMIICGGAPLAKEMEAELRERFPRVVFGQGYGMTEAGPVLAMNLAFAKHPFP
VKSGSCGTVVRNAQVKIIDTETGVSLPHNKAGEICIRGPEIMIGYLNDDEATEETIDKE
GWLHTGDVGYIDDNEELFIVDRVKEIIKYKGFQVAPAEIEALLMQHPSISKAAVVPEKH
ELAGEVPVAFVVRSDGNEISEQEIKAYLEKKVIFYKRIHRVIFTDSIPIAPSGKILRKD
LKARLQQAASA

>Es4CL1-2
MEEYVFRSRLPDIYIPDNMSLHDYCFERLHELRDRPCLIQGSTGKILTYGEVEASSRRV
AAGLRKIGMAKGEVVMLLLQNCPEFVITFLGASMGGNVVTTANPFYTPADIAKQASASN
TRIVVTSSSYVNKLGDLMSGGVRVFTVDEPQEGCEHFSVLTDVEDEGPLENVSVGAEDT

VVLPYSSGTTGLPKGVMLTHRSLVTSVAQQVDGDNPNLNLLPEDVVLCVLPLFHIYSLN
SVLLCSLRAGSAVLLMHKFEIGNLLEFVPKYKISVAAVVPPIVLAIAKNQMVESFDLSS
VRFVLSGAAPLGKELEEALRKRVPNAIFGQGYGMTEAGPVLSMCLGFAKEPTPMKFGSC
GCVVRNAEMKIVDSDTGISLPRNKPGEICIRGAQIMKGYLNDPDSTARTIDEEGWLHTG
DIGYVDDDDEVFIIDRVKEIIKYKGFQVPPAELEALLINHPSIADAAVVPLNDELAGEV
PVAFLVKSENGPGVTEEDIKQFVAKQVVYYKRLHKVNFIHAIPKSPSGKILRKDLKAKL
NEPIPNGEPEAKATP

>Es4CL1-3
MEKQSNNNNSDVIFRSKLPDIYIPNHLSLHDYIFQNISEFATKPCLINGPTGHVYTYSD
VHVISRQIAANFHKLGVNQNDVVMLLLPNCPEFVLSFLAASFRGATATAANPFFTPAEI
AKQAKASNTKLIITEARYVDKIKPLQNDDGVVIVCIDDNESVPIPEGCLRFTELTQSTT
EASEVIDSVEISPDDVVALPYSSGTTGLPKGVMLTHKGLVTSVAQQVDGENPNLYFHSD
DVILCVLPMFHIYALNSIMLCGLRVGAAILIMPKFEINLLLELIQRCKVTVAPMVPPIV
LAIAKSSETEKYDLSSIRVVKSGAAPLGKELEDAVNAKFPNAKLGQGYGMTEAGPVLAM
SLGFAKEPFPVKSGACGTVVRNAEMKIVDPDTGDSLSRNQPGEICIRGHQIMKGYLNNP
AATAETIDKDGWLHTGDIGLIDDDDELFIVDRLKELIKYKGFQVAPAELEALLIGHPDI
TDVAVVAMKEEAAGEVPVAFVVKSKDSELSEDDVKQFVSKQVVFYKRINKVFFTESIPK
APSGKILRKDLRAKLANGL

>EsBDH1-1
MAQQQECKNLIVFGINGHKKEVDLWELHPSTTLLHYIRNYTDFRGPKFGCGEGGCGACV
VHLSKCNPNTGDREEYSVASCLTLVGSLHGCSVTTSEGLGNSRDGYHAIHSRIGGFHAS
QCGFCTPGMSMSIFSALRQAENKANDKSDLHHGFPNLSVNEAHEWIAGNLCRCTGYRPL
ADVCKSFAGDVDLEDLGLNTFWNKNQNENTKDVTSLPSFTSRKVCTFPKFLESVNSSCY
NQIEKPHFNFNYIHKYNGQKYSWIKPTSLDGVFETLNELNCNGNSVPELKLVVGNTSSG
VYRESRPSLFIDISSIPELNFISKKESCIEIGAAVTIARAIESLEDKDSMVGLDHNSVF
KTIASHLKKVASKFVRNTASIGGNLIMAQQLSFESDIATVLLGVGASIKIMASHKTETV
LSMDDFLEKNTQDRKSQLLISIIIPSWNEVGSLKQENIVDEQVLGEDKFLFMDDFLEKN
TQDIKSQLLISIIIPRWNEVGSLKRENIVDEQVLGEDKFLFKTYRAAPRPLGNAVSYVN
AAFLVQVTPAQLYHQWKLGPKIQLAFGAFGTPHAIRSTDVEKFLSGKVMTAEILLQAIE
LLKLCVIPMENTPKAAYRQSVAVGFLFEFFWPLVKDTMVPSKPLCTRKDDGPGLGNLLC
NSKGPVAGKQVLEIHNNYSPVGQPAQKIGVELQASGEAGFVNDIPAPNNCLYGEFVLSA
KPLALVEKINVQHIIDTPNSLSFVSVSDIPVGGKNLAIQSIFGDEPLFADKIVECVGQP
IGLMVANSPLEAKMAAEKVKVHYNCETTGPPILTVEDAVKRESFFQVPDFWSPKPVGDF
QKGMLEAESKIESAEVRTGSQYFFYLETQTALAIPDEDHGMTVYSSTQNPGILQTVIAK
CLATSENNVRVITRRVGGGFGGKAFRAMPVAVACALAAYKLKRPVRMFLDRKTDMLTTG
GRHPVIAKYTVGYKNDGKITALHVDLFINAGFSMDISPMMPRFIVSALKKYNWGSLSID
FKVCKTNLPSKSAMRGPGDTQGSFIAEVIIEHVASALCLDTSLVRERNMHTFESSELFY
ENSIGHPNFYTMPSIWEKLKHSASLERRKKSVDTFNESHCWNKRGLSMVPCFFEVFLNC
RPARVTIFTDGSISVEVGGIELGQGLWTKVKQVTAYVLSQLGDIPSEVIYSKIRVVQHD
TISLAHGGYTAGSTTSEESCAAVEQACKILIERLFTVKKNLTKRKQDQLSWNELISKAS
LQSADLSAQVYWVPDSSARSYVNFGAAAAEVEVDLLTGATTILQVDIIYDCGRSLNPAI
DIGQIEGAFVQGIGYFTTEEVIVDQSGKVVSDGTWTYKVPTVDTVPRKINVELLSSPMN
QKRVLSSKASGEPPLLLASSVHCAIREAIRSARKDQKDNSYFRMDSPATMDVIKSLCGF
HNVEYFLQNLSSK

>EsBDH1-2

MRVEGRKIEEKAVLGNYLRDELGMKGLQMFCKQGGCGSCTVMISYTNQASGEVCHRTVN
SCLLPLCSVDGMHVTTVEGVGSIKDGLHPIQDALVREHGTQCGFCSPGMVMSMYGLSCN
KSVLSPHDIEDGIDGNLCRCTGYRPILNAFQLFACQEKNNCNKEILNNCPNFDIDIEDI
NFNGTSKDVLKKSFILRGGATWIRVGSLETLYNVFQEYKNQRKVRLVRGNTSSGIYPKF
SSDVYVDISQVSELLITTVTRNGITIGGAVSISDFMTVLEENKELSSSYGAILSHLKRV
AHPQVRNLGSVSGNLIITRNHPDFISDIVALLMAAEAKITIGSAYSDKQSVSVSIEEFL
KMEMDDKVIIEIHVPVLPVNSHFVSQKVALRRANAHAVVNTAFKFELFRDKDYINRAVI
VYGGIKPFPQRARECERLLLGKSFKDPKVFEACLQALNKELVPDSSFGQKEYRSFLVNT
LFYKSILSFWALKSLPPRLQTNFTNEERPISSGYVSFDEGDPSEYPVSLPVPKLTAISQ
VTGEVEYLDDIKMGKSWHAKLVVSTVANAKIKSIDPSKALAMKGVMSFLSADTITADGY
CNFISDYECVFAPKKVDYCGQIVGLIVAKTMGIAEAAANLVEVKYIDVKKPILTIKDAM
ESNSFHDSRSFSFEKGNMNDCISKSQFIIEGQVEVGHQFHFHLETQRSLCVPGEEGCMT
VYSSTQNPSKVQQCVAIGLNRPQHKVNVTAKHIGGAFGAKINRSTSVAMACAMAADKLQ
RPVRLLLDISTNMQLVGGRNPYLCRYKVAARENGRIDGIEIQLIMNQGAHFDFEHPDMS
SLLLFIDGVYNVTNWKIEGKVAKTNLPACTYMRGPVFVETTVMIETIIEHVSKNFGLQP
QLVREINMYKKGEELLCGQKVRNWNADLVTDAVKVSSEYENRLKEVQGFNKKSQWVKRG
ISLVPIKFAAVWEAQDMSCLINIHPDYSISLYHSGCEIGQGLDVKVAQVAAMSLGSLIE
DHLEMKNIYVHSTTTIVANNTASTGGSVTSELCAKAAQCESIDSILAYKMEDRINGGR

>EsBDH1-3
MGSIQTDSATTTKEEEQQPILYVNGKRRILQDNLAHKTLLEYLRDLGLTGTKLGCGEGG
CGACTVMISSHDPIKKKTSHRAVNACLAPLYSVEGMHVITVEGIGNQHNLHPVQEALCK
AHGSQCGFCTPGFVMSMYALLRSTKELPDQHQIEESLAGNLCRCTGYRPILDAFRVFSK
TDNSLYLNENSQHVHKDAKFICPSTGRPCDCGGGVTNVNSHIQCSPRPKQLSYSEIDGS
FYSSRELIFPPELLNRKMYPLSLKGLRGLLWFRPLNLKDVLNLRSRFPDSKLVVGNTEV
GIETRFKNMQYKVIIAVTHVPELNILTIKSDGIEIGSSVTLTDLLDTLNQCIKQRSMEE
TSACLAFVEQLRWFAGTQIRNVASVGGNICTASPISDLNPLWIAARANFNIVDSKGTLR
TVTARNFFRAYRTVDLNKGEILLSVFLPFTRKHEYVKEFKQAHRRDDDIALVNAGMRVY
LQQDKDRWAVADVSLAYGGVAPVTLSAVKTEEFLKGKPWTLETLRGALKILQQDIHISK
DAPGGMTEFRQTLTSSFLFKFLLWVAEKVMPQSHELLELPDAFKSAIAPYNRPSSFGMQ
HYENSQIGTAVGHPAMHLSGKLQVSGEAEYADDMPLPPGGLHAALVLSKKPHARILSID
DSAAREITGFEGFFSAKDVPGGNDIGPVIHDEELFASEIVTCVGQVIGIVVADTRENAK
LATQLIKIEYEELPAVLCIEDALKVSSFHCNTEKKLQKGDVDECFSSRSCYKIISGNVQ
VGGQEHFYLEPNSTFIWTVDGCNEIHMVSSTQAPQKHQKYVAHVLGLPMSKVVCKTKRI
GGGFGGKETRSAFISAAAAVPCYHLRRPVKLTLDRDIDMMITGQRHAFLGKYKVGFTED
GKVLALDLEIYNNGGNSLDLSLAVLERAMFHSDNVYDIENVRIRGKVCLTNLPSNTAFR
GFGGPQGMLITENWIEHIARSVGKLPEAIRELNFQKEGSALHYNQKVELCRLQDVWEEL
KISSSFSDLRPEVDRYNKFNRWKKRGLAMVPTKFGISFTTKFMNQAGALVQVYTDGTVL
VTHGGVEMGQGLHTKVAQIAATSFDIPISQVFISETSTDKVPNSSPTAASASSDMYGAA
VLDACEQIKARMQPIAERVKHASFAELALACYLERIDLSAHGFYVTPDIGFDWQSGKGK
PFSYFTFGAACAEVEVDTLTGDFHLRNVDIVMDLGCSLNPAIDIGQVEGAFIQGLGWIA
LEEVKWGDPSHPWIRPGHLFTQGPGNYKLPSVNDIPLKLKVSLLKDAPNPRAIHSSKAV
GEPPFFLASAAFFAIKDAILAARRDAGYDGWFSLDSPATPERIRMACADEFTQPFADMT
FKAKLSV

>EsBDH2-1
MASRRVSSLLSRSFMSSSRSIFSLRGMNRGAQRYSNLAAAVENTITPPVKVEHTQLLIG
GRFVDAVSGKTFPTLDPRNGEVIAQVSEGDAEDVNRAVAARKAFDEGPWPKMTAYERS

KILFRFADLIEKHNDEIAALETWDNGKPYEQSAQIEVPMLARVFRYYAGWADKIHGMTM
PGDGPHHVQTLHEPIGVAGQIIPWNFPLLMLSWKLGPALACGNTVVLKTAEQTPLSALL
VGKLLHEAGLPDGVVNIVSGFGATAGAAIASHMDVDKVAFTGSTDVGKIILELASKSNL
KAVTLELGGKSPFIVCEDADVDQAVELAHFALFFNQGQCCCAGSRTFVHERVYDEFVEK
AKARALKRNVGDPFKSGIEQGPQVDSEQFNKILKYIKHGVEAGATLQAGGDRLGSKGYY
IQPTVFSDVKDDMLIATDEIFGPVQTILKFKDLDEVIARANNSRYGLAAGVFTQNLDTA
HRLMRALRVGTVWINCFDVLDASIPFGGYKMSGIGREKGIYSLNNYLQVKAVVTSLKNP
AWL

>EsBDH2-2
MAALLRTSLLSSCFVAAKSCKPSVFRNLPRRFNDTRCLGTAASSVLEDPITPQVQVKYT
QLLIDGKFVDAASGKTFPTIDPRTGDVIAHVAEGDKEDVDRAVKAARKAFDQGPWPKMT
AYERSRIMLRFADLLEKHSEELAILETWDNGKPYQQSLNIELPMVARFFRYYAGWADKI
HGLTVPADGPYHVQTLHEPIGVAGQIIPWNFPLLMFAWKVAPALACGNTVVLKSAEQTP
LTAIYAAKLFHEAGLPSGVLNIISGYGATAGAAISYHMDVDKLAFTGSTATGKSVLKAA
AESNLKPVTLELGGKSPFIVLEDADVDQAVELAHFALFFNQGQCCCAGSRTFVHEKVYD
EFVEKAKERCLKRVVGDPFKNGVEQGPQIDEEQFNKIMKYINYGKESGATLVTGGDRIG
SKGFYIMPTIFSDVKDDASIATDEIFGPVQSIMKFKSLEEVVKRANDTSYGLAAGLFTK
NIDVANSLTRALKVGTVWVNCFNIYDAAIPFGGYKMSGMGREKGIYGLQNYLQVKAVVS
PLTNPAWL

>EsBDH2-3
MASVWLRSLLSAPRRVAQNSSLFKPCVSRLFPGSISSVRNLGTAASALEEPLTPPVQVK
YTQLLIDGKFVDAESGKTFPTIDPRTGEVIANVAEADKEDVNRAVRAARKAFDEGPWPK
MTAYERSCILLRFADLLEKHTEELAALETWDNGKPYQQALKAEMPMAVRLFRYYAGWAD
KIHGLTVPADGPHHVQTLHEPIGVAGQIIPWNFPMLMFTWKVGPALACGNTVVLKTAEQ
TPLTAIYAAKLLHEAGLPPGVLNIVSGFGETAGSAISHHMDVDKLAFTGSTATAKDILK
AAAVSNLKSVTLELGGKSPFIVMDDADIDQAVELAHFALFFNQGQCCCAGSRTFVHESI
YDEFVEKAKERCLKRVVGDPFKKGVEQGPQIDQEQFNKVMKYIQYGKESGAELVTGGER
IGSKGFFIMPTIFSDVQDNAPIATDEIFGPVQSILKFKTLDEVIKRANATQYGLAAGVF
TKNIDVANTMTRALRVGTVWVNCFDVFDAAIPFGGYKMSGIGREKGIYSLQNYLQVKAV
VSPLRNPAWL

>EsBDH2-4
MANMLILFQFYDAGKTFETLDPRTGEVIAHVAEASKEDVDLAVKAARQAFDHGPWPRLP
GVARGRIMHKFADLIEQNAEELAALETWDNGKPLTLAQNLDLPGSLQMLRYYAGWADKI
HGETLKMEGPFQGYTLHEPIGVVGQIIPWNFPLVMFFMKVSPALACGCTIVIKPAEQTP
LTALFCAQLAKEAGIPDGVLNVVTGFGVPTGQAISMHMDIDKVAFTGSTEVGKAILHAS
ANSNLKRVSLELGGKSPLIIMNDADIDEAVNLANYAIYTNMGQVCIAGSRVFVQEDIYE
EFVKKATQKARERIVGDPFNRQVEHGPQVDKQQYEKILKYIEYGKRDGAKLETGGKKWG
GSGFYIEPTIFSQVMDNMKIAQEEIFGPVMSVLKFKTVDEVIERANKTSYGLAGGVMTK
NIDIANKVSRSLRAGIVWINCFLIIGVDTPFGGYKMSGIGRENGSHGLQNYLQVKSVIT
PLHDSPWL

>EsBDH2-5
MGSFVDASPAPVTVKYNKLFINGEFVDSVTGETFETLDPRTGEVIATVAKGSKEDVDLA
VKAARQAFDKGPWPRLPGIARGHLMLKLAGLVMEHKDELAALETLDNGKPISLCHAMDV
PACGGFLQYYAGWADKIHGQTSKMDGPYMGYTLHEPIGVAGQIIPWNFPMVMFITKIAP

ALACGCTVVIKPAEQTPLSALFLASLVKEAGIPDGVVNVITGFGDAGAAISSHMDIDKV
AFTGSTEVGRLVMEAAAKSNLKPVTLELGGKSPLFIMEDADIDEAVEIAHRAVFTNMGQ
VCLAGTRIFVQESIHDAFVKKAAERARKQIVGDPFRKDVDHGPQVDETQYKKILKYVEY
GKKQGARLVEGGNSCSPNGKGYYIQPTIFCNVEDDMKIAQEEIFGPVMSVLKFKTVEEA
IERGNKTIYGLGAGVVTNNMNVANRMSRSLRAGTVWINCYLVQGLDVPIGGFKASGFGR
EFGEYNLHSYLQVKSVITPLQDSPWL

>EsKAT1-1
MEKAIERQRVLLEHLRPSSSSSHNYEASLSASACLAGDSAAYQRTSLYGDDVVIVAAHR
TPLCKSKRGNFKDTYPDDLLAPVLRALIEKTNLNPSEVGDIVVGTVLAPGSQRASECRM
AAFYAGFPETVAVRTVNRQCSSGLQAVADVAAAIKAGFYDIGIGAGLESMTTNPMAWEG
SVNPAVKKFAQAQNCLLPMGVTSENVAQRFGVSRQEQDQAAVDSHRKAAAATAAGKFKD
EIIPVKTKLVDPKTGDEKPITVSVDDGIRPTTTLASLGKLKPVFKKDGTTTAGNSSQVS
DGAGAVLLMKRSVAMQKGLPVLGVFRTFAAVGVDPAIMGIGPAVAIPAAVKAAGLELDD
IDLFEINEAFASQFVYCRNKLGLDPEKINVNGGAMAIGHPLGATGARCVATLLHEMKRR
GKDCRFGVVSMCIGTGMGAAAVFERGDGVDELRNARKVEAQGLLSKDAR

>EsKAT1-2
MNRIHMRQEVLLNHLRPQVAPRSSTDLVASACSAGDSAAYLRNGDFGDDVVIVAAYRTP
LCKSKRGSFKDTPADDLLAPVLKAVIERTGVNPAEVGDIVVGTVLAPGSQRASECRMAA
FYAGFPECVPVRTVNRQCSSGLQAVADVVSAIKAGFYDIGIGAGLESMTINAMAWEGSV
NPKVEMNQKAQDCLLPMGITSENVAERFGVTRQVQDKAAVESHKKAAAATATGRFKDEI
IPVETKIVDPKTGEEKLVTISVDDGIRPNANLADMAKLKPAFKKSGTTTAGNSSQVSDG
AGAVLLMKRSKALEKGLPILGVFRSFAVTGVDPAIMGVGPAVAIPAAVKAAGLEIEDID
LFEINEAFASQFVYCAQKLNLDTEKINVNGGAMALGHPLGATGARCVATLLHEMKKRGK
DCRFGVISMCIGTGMGAAAVLESGGSVDQLSNAKPISKHNLLSRDAK

>EsKAT1-3
MERSRIHMRQEVLLNHFRPQVASRTTADLVASACFAGDSAAYLRNGEFGDDIVIVAAYQ
TPLCKSNRGSFKDTPADDLLAPVLKAVIERTGVNPAEVGDIVVGTVLAPGSQGASDCRM
AAFYAGFPETVPVRTVNRQCSSGLQAVADVVSAIKAGFYDIGIGAGVESMTVNAMPQER
SVNLKVEMNKKAQDCLMPIGILFENFAERFGVSRQVQDKAAVESHRKAAAAIATGRFKD
EIIPVQTKIVHPITGEEKLVTISVDDGIRPNANLADMAKLKPAFKNSGTTTAGNSSQVS
DGAAAVLLMKRSKASEKGLPILGVFRSFAVTGVDPLDTGVGPAVAIPAAVKAAGLEIDD
IDLFEINEAFASQFVYCAQKLDLDTEKVNVNGGAMALGHPLGATGARCVATLLHEMKKR
GKDCRFGVISMCIATGMGAAAVLECGGSVDQLSNAKPISKHNLLSRDAKQ

>EsKAT1-4
MEKFQTRQQVLLGHLLPSSSSSSSSHSNLRYSVCGGQQNVRNEDDIVIVAAYRTPICKA
KRGGFKDSRPEDLLEPVLRAVVEKSGIKGEEVGDIVVGTVLAPGSERAIECRIAALLAG
IPASVPIRTVNRQCSSGLQAVADVAAAIKFGSYDIGIGAGVELMSADLVGIPPVKNAKL
EANRDARDCLLPMGMTSENVAERYGVTREEQDLAAVQSQKKAASATATGRFEDEIIPVK
TQIVDPKTGERTNVIISVDDGIRPNTSMSDLAKLKPAFRKDGSTTAGNASQVSDGAGAV
LLMKRATANRKGLPILGVFRSFVAVGCEPAVMGIGPAVAIPPAVKAAGLEIKDIGLFEI
NEAFASQYVYCCKKLELDEKKVNVNGGAIALGHPLGATGARCVGTLLHEMKKRGNDCRF
GVISMCIGTGMGAAAVFEKEI

>EsKAT1-5

MDYSKLATNGSSLCAKSDDDVVIVAAYRTPICKAKKGGFKDTRPEDLLEPVLRAVVEKT
GVKAEEVGDIVVGTVLAPGSHRAMECRVAAFLAGFPETVPIRTVNRQCSSGLQAVADVV
AYIKAGFYDIGIGAGVELMSETPLRFDPIYNPKLETNKYAKECLVTMGITSENVAERYG
ITREEQDLAAVRSHQNAAAAIAAGKFKDEIIPVLTKIVDKDSGESKAVTITADDGIRPD
TSMPGLAKLKPVFKEGGSTTAGNCSQVSDGAGAVLLMKRSIATKKCLPILGVFRSYAVV
GCEPSVMGIGPSVAIPPAVKAAGLGIEDIGLFEINEAFASQFVYCCKKLGLDQEKVNVN
GGAIALGHPLGATGARSVATLLNEMKRRGKGCRFGVISMCIGTGMGAAAVIENESALE

>EsCHD1-1
MSNVWVTIDVGSDGVALITISNPPVNALAPTILAGLKEKYDEAVRRDDVKAIVVTGNAG
KFSGGFDINVLRKVQATGDTSHLGRVSVDLMMNTIEEAKKPSVAAIQGLALGGGLELAM
SCHARISAPGAQLGLPELSLGIIPGFGGTQRLPRLVGVKKAVQMMLTSKPITTDEGKKL
GLIDQIVPPGELMATARKWALDIADRHKPWLVTLSRTDKLEPLAEARAIINAARVQAKR
TAPNLPHPQVCLASIEEGIVSGGVAGILKEEQGFFEVALSDTAKGLIHMFFSQRSTSKV
PGVTDLGLKPRKISRVAVIGGGLMGSGIVTAFIHNNIPVILKEVNEKFLQQGLNRISAN
LKSYVRKGILKQEKAEKAMSLVTGTLDYSEFKNVDLVIEAVIEKISLKQEIFSDLEKIC
SPHCILATNTSTIDLNVVGAKTQSQDRIIGAHFFSPAHIMQLLEIVRTDKTSPQVVLDL
LNVGKIIRKVPVVIGNCTGFAVNRMFFPYSQAAHMLANLGVDIYRVDKVIKGFGMPMGP
FRSGVFFKII

>EsCHD1-2
MGRGRADMEVRPDGVAVITISNPPVNSLSVEVLLGLKTKLEEALQRHDVKALVLTGSGG
KFSGGFDITTLLDLQNGIVPRGVEQLQMEFADISVKLITEFLEGARKPLVAAIDGLALG
GGLEIAMACHGRISTPQAQLGLPELQLGVIPGFGGTQRLPRLVGLSKALEMILLSKPIK
SEEANELGLVDAIVPPSELLNTACRWALDIAEYRKPWLISLYRTDKIEPLGEAREIIKF
TRMQAQKRAANFKHPLVCLHVIEEGIVSGPMVGLLKEASSFQELLHTDTAKSLVHVFLA
QRATSKVPGVTDIGLEPRKVRKVAVVGGGLMGSGIVTALILSGYPVVLKEVNTNFLNAG
LGRIKANLQSRVKKGKMSEEKYEKVISLVQGTIEYDKFSDIDMVIEAVIENLKLKQQIF
SDLEKICPEHCVLATNTSTIDLNLIGEKTNSQSRIVGAHFFSPAHIMPLLEIVRTEKTS
SQVIIDLMDVGKKIKKTPIVVGNCTGFAVNRMFFPYTQSALFLIDLGLDVYQIDKTITG
FGMPMGPFRLADLVGFEVAVATGMQYLENFPDRVYKSLLIPMMLEDKRAGEKTLKGFYI
YDNKRKARPDPEIKKYVEKSREVAGLITKDKPISLTSKDIIEMIFFPVVNEACRVLDEG
IAVQASDLDIASVMGMGFPPYRGGLMFWAESLGSSYIYSRLKSWAEAYGDFFRPSSFLE
ERARTNAKLSAPKNRMTSRL

>EsCHD1-3
MGGEAITCVQKGADGVAVITFDNPPVNALSVPVQNALYKIFIQLHEDKDVRAIVLTGAN
GKFCGGADIRGFQEVQKKGTAEKPMVSGYTLVNDVIEGSPKPVVAAIEGFALGGGLEIA
MACHARVSAPNVQLGLVELQLGIIPGLGGTQRLPRLVGVEKAIDMLLAAKTFNSNEALK
FGLVDSVVSNSGLIATARKLAIDICECSYKWERSLHKADKIPSLPELADLFKEAREKLK
KTHRNVRYPSVLLDVIEEGLLKGGIAGSSLESRAFPDLVKYPEAKALMHVFFAQRSSSK
IPGITDQGLRPRNIRKAAIIGGALMGSGIATALALAKIPVIVKEVNSKEMDAAIKRIHA
NLSNRARSGKISEEDAKHCMSLVKGVLDYKSFFDVDIVIEAVTENIPLKQKIFHDLELA
CPPHCILATNTSSIDLEIIAANLKSKDRLIGAHFFSPAHVMRLLEIVRSNTTSVQSIVD
MLNLTKVLNKVPVVVRSTPGFAVNRVFFPYGMGAHFLANLGVHPYRIDSVIKEFGMPMG
PFRMADLGGLQTSLLVGHIWKKAFPDRVYFSALMPMLYEDKRLGESHGKGYYNYQGRKE
QPAPELEHYLEKSRKAAGFSESHKITSITDQEIVEMILFPVINEASRVLEERVVESSSD
LDIATVLGMAFPAYRGGVVFWADLVGAPYIYSKLQKWAEKYGSFFTPSKALEACVVLKK

PLAQIPIRNASRL

>EsBL1-1
MEGVCGELKRCEANYAPLSPLTFLDRAEVSYGDRTSVIYGKSVRFTWAQTADRCRRVAS
SLVTYHNIERGDVISVLAPNVPAMYEAHFAVPMAGAVLNTINTRLDARNVATILSHCNP
KLFFVDYQFVPLAKQALSLLGARHPRVIVLNDDDTTQSMYEDLVRNGDSDFRGVRIQDE
WESISLNYTSGTTSAPKGVVYSHRGAYLSCLSMLLLWNMDTYPVYLWTLPMFHCNGWTF
TWALAARGGTSVCLRTMSKEEVYSAILNHSVTHMCCAPTVFNMLLADPRPMPTKVRVIT
GGAPPPPAVLHKIEALGFDVTHAYGLTEATGPALVCEWKDEWNELSAADRACMKRRQGL
PVLSLSEMDVKDPDTMKSVAKDGVQTGEIMLRGSGIMKGYLKDVEKTDAAFSGGWFRTG
DVAVVHGDGYVEIKDRSKDVIISGGENISSVEVETVLHEYPNVTEAAVVAMPHQHWGET
PCAFIRPSQGAVLSEKEILMFCKDRLPGFMVPKRVVFKEGGLPKTATGKVQKDVLRAEV
KKLVSVNTAKGSRSTKSTEVKHQQQHHQAQLMVELAPARSRL

>EsBL1-2
MEGEHGELKKCHANYVPLSPLSFLDRAETTYGDRTSVIYGKSRRFTWAQTADRCRRVAS
SLISYHGIKRGEVISILAPNVPAMYEAHFAVPMAGAVLNTINTRVDARNVAAILSHCNA
KLFFVDYQFIALAKEALGLLAHRSGIIVVNDDDATQSMYEDLVRNGNPHFRGVGIQNEW
ESISLNYTSGTTSAPKGVVYSHRGAYLSCLSMLLLWNMDTYPVYLWTLPMFHCNGWTFT
WALAARGGTSVCLRTLSAELVYSQIIEHSVTHMCCAPTVFNMILADPRHLPTKVRVITG
GAPPPPAVLHKVEALGFDVTHAYGLTEATGPALVCEWKEDWNKLSPSERACMKRRQGVS
VLSLSEMNVKDPDTMKSVAKDGAQTGEIMLRGSGIMKGYLKDVEKTDSAFSGGWFRTGD
VAVVHGDGYVEIKDRSKDVIISGGENISSVEVESVLHGYPNVAEAAVVAMPHQYWGESP
CAFITPSKGSVLNEKEILKFCKERLPGFMVPKRVVIEKEGLPKTATGKIEKNVLRAEAK
KIASVTVMNEYQHLKAELPCIVESALARSRL

>EsBL1-3
MEGLKKCRANYDALTPITFLERSGIVYSEETSIVYGDVKFSWGETLDRCVRLASSISSL
GVHVGDVVAVLAPNIPAMYELQFGAPMAGAVLCCLNIRQEAHAISVLLRHSEAKVLFVD
YKFLDMAEKALEILSKSNAKVPLIILINEPNSRTLAREKESGLEYESLLNSGDPNFEIK
KPEDELQPFALNYTSGTTANPKGVVYSHRGAYLNALSAAFMWGLTKSSVHLWTVPLFHC
NGWSIAWAVAALGGTNVCLRNVTAGAIFGEIAKHGVTHLGGAPTVLNIIANAKPQERKH
FTNRVEVLTGGAPPPPQILSTMDKLGFHVTHSYGLTETYGPASISLWKSQWDTLPDEEK
ARLHARQGVRHLGLSGIDVKDPETMQSVPQDGKTIGEVMLRGNTVMMGYLKDTKATEAA
FDGGWFHSGDLGVMHSDGYIELKDRSKDIIISGGENISSIEVESVLYSHPDILEAAVVS
RPDNYWGETPCAFVKLKENKVVSSDSIIAFCRERLPHYMAPRTVIFEDLPKTSTGKVQK
HVLRDKAKALGSLSKSRQSKL

>EsBL1-4
MNKMEKREANHPALSPIGFIERAATVYGDSTSVIYGNLRFTWSQTYERCRRLASALVAR
NISPQDVVSVVAPNVPAIYEMHFAVPMTGAVLNCINIRLDARAMAAQFAHCRPKMVFVD
YQFAATVSEALKELRDTVMVVIAEDDGDEANWSATYEGLVREGDPQYEMRWPDDEWDPI
ALNYTSGTTSAPKGVVHSHRGIYTMATDSLVLWGVTSAPKAVHLWTLPMFHANGWCFTW
AMAAVGGTNVCLRRFDASDIYSAIAEHGVTHLCGAPVVLSMIANAATNKSLNGRRVEVL
TAGSPPPAAVLKSMEDVGFSVTHGYGLTETAGLVICCAWKPEWDSLPAEERARLKARQG
VRVPCVTGVDVVDPVTMKSVARDGMSLGEIVLRGPSIMKGYLGNPEATEKALHGGWLHT
GDVAVMHADGYVEIKDRSKDVIISGGENISSVEIESVLYSLEGVMEAAVVAMPHPHWGE
TPCAFVVPKTGAVLSEERVVGLCRLKLPGYMVPKRVVVVKESDGGLPKTSTGKIQKFML

RDLARSLSLSSPTSKL

>EsBL1-5
MEANNQALTPLSFIERAERVFGERTSIVYGSQRFSWSETAQRCRRLASVLTSSLDIKPG
ETVAVVAPNIPATYEMHFGVPMAGAVLSTLNIRLDASTMAAILEHSEAKALFVDYQYLE
IAKAALRSLLQSRKKVPKLVIIAEQQGQLESTAGCNASDMEYENLLEEGNPNCEIYWPI
DECEAITLNYTSGTTSAPKGVLYSHRGAYVNTLSTLLMWEMKSRPRFLWTIPMFHCNGW
NFPWGIAAQGGTNICLRSVQAKEIFEAIDEHRVTHFGAAPTMLSKLANAPPNERRPLPH
RVHVMTGGSPLPPPLLSKIENMGFDITHIYGLTETYGPMSSCALNPEWDLLFYDAQAKL
RSRQGIPHVGVFKLDVKNPNTMEDVPHDGVTIGEVMVKGNTVMKGYYKDEEATSKAFEG
GWFHTGDLGVIHPDGYLELKDRAKDVIISGGENISSIQVETVIHCIPFVAEVAVVGRPD
DYWGETPCAFVKLNENCNAKPQEVIDYCRDHLPHYMAPRTVIFEDLPKTPTGKIQKHIL
REKAKKLGTIFPGNYVKH

>EsBL1-6
MVRDYKDIDDLPKQAANYTALTPLWWLERAAQVHPNHTSIIYNDLRHTWSQTYARCRRL
ASALRNHSIGTGQTVAVLAPNTPVSYETHFGVPMCGAVLNAVNIRLDARTVSFLLGHSA
ASAVIVDQEIYPLLQDALRRPGEKSVQPLIIVVRDPACDAKNFDLAIRDGGVIEYEEFL
GSGDAEYKWQPPSDEWQSIALGYTSGTTSDPKGVVLSHRGAYVGSIGSSLAWDMGQKTV
YLWTLPMFHCNGWCYTWAMAAHAGTNVCLRQVTAKGIFDAIAEHRVTHFCAAPVVLNTI
VNSAPSERRQIPSGVNVMTAGASPPASVLAAMESMGFNVTHTYGLSETYGPSTVCAWKG
DEWDALPLTERARLKARQGVRYVSLECLDVVDPTSMKPVPADGCTVGEIVMRGNMVMKG
YLRNPRANEETFRGGWFHSGDLAVKHPDSYIEIKDRSKDIIISGGENISSLEVENAIYA
HPAVLEASVVARADEQWGESPCAFVTLKDGGDGAKLSGDIIAFCRSRLPRYMVPKSVVV
VDSLPKTATGKIQKHVLRSKVKEMGPIKRSRL

>EsThDPC1-1
MAMASLKMNAVTMGTHVALARNMQLCPKSFPCIQNIHNRGQKINRKSARLQAEKSATIP
IAQSGSMQELSEIVVAPNPYKSRYADHEPRKGADIIVEALEREGVEHVFAYPGGASMEI
HQALTRSKTIKNVLCRHEQGEIFAAEGYAKSSGKVGVCIATSGPGATNLVSGLADALLD
SVPLVAITGQVPRRMIGTDAFQETPIVEVTRSITKHNYLIMDVDDIPRIIKEAFYLASS
GRPGPVLVDIPKDIQQQMNIPNWNNAMKLDGYIQRLPKAPEDSQLQQILRLLYSSKKPV
LYVGGGCCSASAELRRFVELTNIPVASTLMGLGNFPSSDPRSLGMLGMHGTVYANYSVD
KSDLLLAFGVRFDDRVTGKLEAFASRASIVHIDIDPAEIGKNKQPHVSLCADMKLALQQ
LNSLLERNAERLDFSLWRKELDEQKAKWEMNFQEVGDLIAPQHAIQLLHQLTDGKAIVS
TGVGQHQMWAAQWFKYDQPRSWLTSGGLGAMGFGLPAAVGAAVANPGMLVVDIDGDGSF
IMNVQELATIRVEDLPVKVMVLNNQHLGMVVQWEDRFYKANRAHTYIGHPTVEGEIFPN
FLKMAEACSIPAARVSKKSDLKDALIKMLNTPGPYLLDVIVPHQEHVLPMIPAGGSFND
IITEGDGRREY

>EsThDPC1-2
MEASVIQNFSKSGTSHFMPWVKPLPLTKMNYGRFRVSEWRVTRLRSSLIESHSTKTSEE
IIDNSREDVSCTANQLNGAFESRNEPRKGADILVEALEREGVEHVFAYPGGASMEIHQA
LTRSGMIRNILCRHEQGEIFAAEGYARSSGRVGVCIATSGPGATNLVSGLANAMMDSVP
VVAITGQVPRKLIGTDAFQETAIVDVTRCITKHNYLIMDVADIPRVMKEAFFIASSGRP
GPVLVDFPKDVQQQMHIPNWTIPMKIPGYVQRLPGVPNFTQLQIIFKMIVESKKPVLYV
GGGCMNSSREMREFVEITGIPVATTLMGLGCFPSSHPRSLGMLGMHGSVYCNYAIDNAD
LLLAFGVRFDDTVTGKLESFATRAKIVHVDIDPAELGKNKVPHVSVCADMVCALEHLNE

LLKRNRFKSCLDSWVQELELQKEKWPLSFNDHGNATIQPQYAIWMLHEASNGNVIVTTG
VGQHQMWAAQWFKYERPRQWLTSGGLGAMGFGLPAAVGAAVAHPGVTVVDIDGDGSFLM
NAQEMAIVRTENLPIKIMILNNQHLGMGVQWEDRFYKANRAHTYIGNPSQGDEIFPDFL
KMAESCLIPSARVKDKAQLRDAIVTMLETPGPYLLDVVVPHSEHVLPMIPAGGSFKDTI
THGDGTQSY

>EsThDPC2-1
MSCCDVGGAPTKPVTGGPERPVMVPINADAKASSATLGRHLARRLVQIGCDSIFSVPGD
FNLTLLDHLIAEPGLNVVGCCNELNAGYAADGFARSRGVGACVVTFTVGGLSVINAIAG
AYSENLPVICIVGGPNSNDYGTNRILHHTIGIPDFSQEFRCFQTVTCAQVIVNNLDDAH
ELIDHAVSTALKESKPVYISVSCNLPAIPHPTFAREPVPYCINPSLSNPRSLEAAVDAA
AEFLDGAVKPVLVAGPKLRVAKAGDAFMHLADTSGYALAVMPSAKGQVRENHPHFIGTY
WGAVSTAFCAEIVESADAYVFAGPIFNDYSSVGYSFLLKNEKSILVHPNRVTIGQGPTF
GCVLMKDFLEALAKKVKRNQTAFENYQRIFVPDGVPLQSAPEEPLRVNVLFKHIQAMLS
NDSAVIAETGDSWFNCQKLKLPERCGYEFQMQYGSIGWSVGATLGYAQATPSKRVIACI
GDGSFQVTAQEVSTMIRNGQNSIIFLINNGGYTIEVEIHDGPYNVIKNWNYTGLVDAFH
NGEGKCWTTKVKTEEELVAAIKNTQGPKKDCLCFIEVVVHRDDTSKELLEWGSRVAAAN
GRPPNPQ

>EsThDPC2-2
MELVDACGYAYAVMPSAKGQVLETHPHFIGTYWGAVSTAYCAEIVESADAYLFAGPIFN
DYSSVGYSFLVKREKAVIVNENRVKIADGATFGCVLMKDFLEALSKKLRKNTTAFENYQ
RIFVPEGEVPKSAPKEPLRVNVLFKHIQALLSDDTAVIAETGDSWFNCQELKLPEKCRY
EFQMQHGSIGWSVGATLGYAEAAKDKRVIACIGDGSFQVTAQDVSTMIRNNQKSIIFLI
NNGGYTIEVEIHDGPYNVIKNWNYTGLVDAFKNGEGNCWTVKVKTEEELLQAIETASGP
KKDDLCFIEVLAHRDDTSKELLEWGSRVAAANGRPPNPQ

>EsTA1-1
MANLLSNSQPGSNFVGSRRHPSFQSRFGDVMISVSANPSSRKKVYRSTHIISMASLENG
KLPVDLKLSPRVSSLRPSKTMVISDHATALVQAGVPVIRLAAGEPDFDSPDPVVEAGVK
AIQEGFTRYTPNAGTMEIRTAICHKLKEENGLTYTPDEILVSNGAKQSIMQAVLAVCSP
GDEVIIPAPYWVSYPEMARLADATPRIVPTSVSDNFLLKPEVLESVLNEKSRLLILCSP
SNPTGSVYPKEVLEEIAKIVAKHPHLLVLTDEIYEHIIYPPAKHTSFASLPGMFEKTLT
VNGFSKAFAMTGWRLGYLAAPKQFVTACGRIQSQSTSGPSSISQKAGVAALKLGYAGGV
VVSTMVQAFRDRRDFLVEKFQAMDGVKLSEPQGAFYLFPDFSSYYGTKVEGFGVIDGSD
ALCKFFLEKAEVALVPGDAFGNNDCIRISYAASLDVLRTAIGNIENAMKLLKPCTEKTL
V

>EsTA1-2
MEMEALNPSVASLTPSNIGFIFSKVHQMKRQGKPVIILSVGEPDFDTPSPISQAGKEAI
DEGYTRYLPTPGALDLRTSISHKLKEENGLEYSPDQIVVSNGAKQSIMQAILAVCSPGD
EVIIPAPYWECYPGMVQLANASSVVISTYLEDDFLLKSESLSAILTERSRLLILCSPSN
PSGTVYPVDRLKAISKVVSQHPRLLVLADEVYEKIIYPPAMHTSFASLPGMWPRTFTVN
AFSKSFAMTGWRLGYLAAPTRFAMACCRIQEQLSSCANSIAQRAGLVALQSNSVKDSVT
MMVKAFEERRDYIIKRLTSINGVKLSIPMGAFYVFPDISYYFGHKYEGTATIDDAESFC
RYLLENAEVALVPGSAFGTPTCVRISYAASYESIQEALDRIEKALALLSPPTTQNGVAG
D

>EsTA2-1
MASNGEWSNDNTSLRGVVGTLMRQAHIAEAQGKTIISLGQGDPSTYSCFAAPKNAQDAL
VKCTLSSLYNGYAPSLGLPQAKKAVADFLSKDLTYKLSPNDVYMTAGCSQAIQICLTVL
SSKSPNANILLPKPGFTLYQSLAAEIGIEAIFYDLIPEDEWKVNLDQVRQLADENTTAI
VVINPNNPCGSVFSREHATEIAQTARDLGISIVSDEIYAHIVYGDYEFVPMAEFASIVP
VITLGGLSKRWLIPGWRIGWLAICDPNGFLKPKKVQQAIEVLMNITPTPSTMIQAAVPS
VLQDTCQEFHEQTLQLLKTAANICYERIQKIDALSCYSRPNGSMFIMAKLNLHLLKGIK
DDMDFAWHLMNEEAVLVVPGFILGMKDWIRISFAAPSTLLEEAWDRIESFCCRSSYL

>EsTA2-2
MECHDMLGIRGILTDIRAQLDPNSKTPISLGVGDPAVFECFRTPMESEVALTKAIASGN
FNTYAPSHGLEQSRRAVAKHLSKGLSYDLSGDDVYMTCGTSQAIDIIISVLASKEANML
LPRPAYPKYEAFLAYHGIEQRYYDLVPERDWEIDLNQLAAIADNNTIAMIIINPNNPCG
SVFRLEHLLMVSQIAKRLGFLIISDEVYADIVFNEAEFFAFRRLSHIVPVITIGSISKK
YAVPGWRLGWLVTCDPHGILKASQVTDAIKKLVNFVMDPATIIQAAVPEILENTPEQFH
MQLLNAFSEGADIMYDRIQNIKCLHCPSKPRGSMFSMIKIDFSGLLDIHDEIGFAMQLA
REESVIVVPGTPLGLKNWIRVSFGIPPNLLKEGLERIEAFCSRHVRSL

>EsTA2-3
MVKWNFKPSKHLMECHDMLGVRGILQDIKAQLDPNGKTPIPIGHGDPAGFQCFRIPPEA
EAALHQTIASGKFNTYSSPEGLLQARRAVSNYLSQGLPYDLSVDDIYLTCGSTQAIDIA
ISVLASKGANILLPRPSYPQYEALLAYYSIEHRFYDLVPERGWEINVDQLHAIADKNTI
AMIIINPNNPCGSVYTSEHLLMVAQTAKHLGFLIIADEVYGDIVFNDAKFFPMRNFATT
VPVITIGSISKKYAVPGWRVGWLVTCDPHGVLKASQVTGAIRKLVNFVTDPATIIQAAI
PAIIENTPDQFHMQMLQYFSEAADILYDSIQNISFLHCPSKPRGAMFSMIKIDFSGFLD
LQDDVEFALKLAREESVVVLPGTPLAMKNWIRVTFGVPPKLLKEALERMEAFCRRHFRS
L

>EsRED1-1
MAAKRMEGKVAIVTASTQGIGLAIAERLGLEGASVVVSSRKKNNVDEAVETLKSKGIDV
LGVPCHVSNRAQRQELIRATVEKYGHIDILVSNAAVNPTTDGILEIPESVLDKLWEINV
KATILLVQEAAKHLTKNSSVIIITSIAGYSPQPSMSMYGVTKTALLGLTKALAVEMAPD
TRVNAVAPGFVPTHFADFLMRSEGIRKELEEKTLLGRLGKTTDMASTVAFLASEDASYI
TGETIIVAGGVQSRL

>EsRED1-2
MPQRISFPSTKKSLCTGIKAQIASVEKADVESPKVLEAPVAIVTGASRGIGKAIALALG
TAGCKVLVNYARSSKEAESVAKQIEENGGSAVVYGGDVSKEADVDAMIKTAVDTWGTID
ILVNNAGITRDTLIMRMKKSQWQEVIDLNLTGVFLCTQAAAKVMMRKRKGRIINISSVV
GLVGNVGQANYSAAKAGVIGLTKTVAREYASRNVTANAIAPGFIESDMTATLGEEIEKK
ILESVPLGRYGKPEEVAGLVKFLALDPAANYITGQVFNVDGGMVM

>EsRED1-3
MESPFKESVLRGKVAIITGGATGIGFEISTQFGRHGASVVLMGRRKNVVEQAAMSLQGQ
GIKAIGLAGDVRKKEDATYVVESTIRHFGKLDILLNGAAGNFLVSPEDLSPNGFRTVLD
IDTVGTYTMCHVALPYLKKGGVGKDPNDAGVILNISATLHYTAAWYQIHVSAAKAAVDS
LTRSLALEWGTDYGIRVNGIAPGPIKGTPGIEKLAPDELEGQHTKQPLYRLGEKWDIAM
AAIYLASDAGTYVNGATIVVDGGLWLSRYRFVSKDVVRLVSRTVETRSRKDNARPSSKL

>EsRED1-4
MASKGEEGQTRWLEGRGALVTGSSSGIGREIALTLANHGAKLVVHYSSNHAGANEAVEL
IKTNGGRAVAVKADISDPEQVKRLFDEAEAALGQLHVVVNNAGVIDNNYNKIMDMPVED
WDRIFTINSRGTFLCTREAARRVVRGGGGRIINITSTVAATNPVKYGAYAASKASVETM
TKVLAKELRGSGITANCVAPGAVDTEFFFKGKPQELVESLGKAPPFERLGKVTDVAPLV
AFLSSDQGEWVNAQVVRVNGGVA

>EsRED1-5
MEGECIYKGLGGKVALVTGASSGLGRQFALSLARHGCNVVAAARRKDMLSLLCNEINSM
NSGVTAKAVELDVTKSTANIELAVSEAWNLFGFIDILVNNAGFRGSVTSPIELTEEQWD
KEVITNMKGSWMVSKAVTKRMRDTGNGGSIINISSIGGLERGELPGGTAYIASKAAVNA
FTKIMALELGPYNIRVNAVAPGLFRSEITAGLMEKDWLATVARKIVPLQTYGEVDPSLT
SAILFLSSDSSAYITGNVFIVDGGQSLPGVPLYSSL

>EsRED1-6
MDNQSQEYPKYADLHDKVVLVTGASSGLGREFAIAFAKHGCRVVVTARRQDLLTSLCDQ
INNENEDVSSKPRAMAVPLDVSDNENHIDTAIEKAWKCFGSIHVLINNAGYRGSVRNPL
KYDENEWNAVMNTNVRGVWLVSKCVVKRMKEASIKGSVVNISSTAGLARGNLPGSMIYG
ISKAAVIQMTKIMALELGGYGIRINAIAAGIFRSDVTKGIFENQSTFKVAEKIVPLGRW
GNTNPDLTGLLFLLASDSSSVITGNVFIADGGATLPGIPLWSSL

>EsRED2-1
MAAKRMEGKVAIVTASTQGIGLAIAERLGLEGASVVVSSRKKNNVDEAVETLKSKGIDV
LGVPCHVSNRAQRQELIRATVEKYGHIDILVSNAAVNPTTDGILEIPESVLDKLWEINV
KATILLVQEAAKHLTKNSSVIIITSIAGYSPSPSMSMYGVTKTALLGLTKALAVEMAPD
TRVNAVAPGFVPTHFADFLMRSEGIALEIF

>EsRED2-2
MAGRALEGRGAIVTGGSAGIGKEIARALAAEGAFVIVNYSNNAHGAQQTVDAINKDGEK
RAHAVKANVSNEGDVRRLFKEAEAAFGGPGKLHILVNNAGVTDTTLPTVADTSVDGWDR
IFAVNCRGTFLCSQEGARRIVRGGGGRIINITSDAVGHLKEGHGAYGASKAAVETMTKI
MAKELRGTLITANCVAPGPISTDMFFVGRSQDTIDRAVADCPLERLGKVEDVAPTVVFL
ASDKGEWVNAQVVRVNGGYVS

>EsRED2-3
MHLFDEAERFLGDNLHIVVNNAGVMDTKYPTVANTEVEEWDRTFEVNCKGTFMCSREGA
RRVSKSGGGRIINVSTSVVGTLMPGYAAYAASKAAVETMTKILAKELRGCRITANCVAP
GPIETEFFYAGKSPQQISAIAKSAPLERLGKPEDVAPVVAFLASDQGEWVNAQVVRANG
GLV

>EsRED3-1
MENFAVLNDGNKIPLVGLGTGTMDTSDHVVEQAVLAAVQAGYRHFDCADVYGSKIVLGN
ALNKAFSEGLVARNEVFITSKLWCSDLHPDDVLPTIHRILKEMRLEYLDLFLIHWPVRV
KKDAPIMRYDSDSFLPVDIPATWKSMEKCVEMKLTRSIGVSNFSAKKIKDLLVSASIPP
AVNQVEMHPNWNQKSLREFCKENNVHVTAYSPLGAQSNFSFINASGILTHPHVLSIASK
HGKTPSQIVLRWEIEEGVSVIPKSCNAERIRENINIFDFCLDEDDKELMNKMEQRKVFA
GDAFVNPEGGPYHTLFELWDEELPN

>EsRED3-2
MGEASTQMSYELNNGTRIPAVGLGTWQSPPGAVASAVKAAIKVGYRHIDCAHIYGNEKE
IGSALKEMFDANIVKREELWITSKLWLADCAPDKVSNAVDVTLKDLQLDYLDLYLIHWP
NENVASTWQQMEELLDTGKVRAIGVSNFSVKKLQDLIKTAKVIPAVDQVECHPAWQQNK
LHEFCKSNNIHVTAYSPLGSPGSEWMKQPVKVLEHPVVKDTAEKLGKSPAQVALRWGLQ
SGHSVLPKSCNENRIKENFDVFGWSIPQEMFDSFKQIDQVRFLRGDFLISRGMFKSPED
LWDGEI

>EsRED3-3
MAQNLRVVEQEYPWAVTSHGRYKLLSGNEIPAVGLGTWKSGNAANYSVFSAIVQDGYRH
IDTAAEYGVEEEVGHGLRAAMEAGIDRKDIFVTSKLWCTDLSPERVRPALNKTLNDLQL
DYLDLYLIHWPFRLKEGASSPPKAGDVLDLDMEGVWRAMEKLVQDKKVREIGIANFTVK
KLESLLDYASIMPSVCQVEMHPGWRNDKILEACRKHNIHVTAYSPLGSHTRDLIDDPTV
QLVAKKLNKTPGQVLIRWAVQRGTSAIPKSTNPDRISENVKVFDWSLPPLEFEALSSLP
QQKRELDGSNLFVCPEGPYTTVEMLWDGEGC

>EsRED3-4
MATITLNNGNKMPIMGLGVWRMENHLVRDLILNSIQLGYRHFDCAADYKNEKEVGEALA
EAFQKGLVKREELFITTKLWNSDHGHVLEACKDSLKKLQLDYLDLYLIHFPVATKHTGV
GTTGSALDENGVLAIDTTISLETTWHAMEELVSAGLVKSIGISNYDIFLTRDCLAYSKI
KPVVNQIETHPYFQRHSLVQFCQKHGIAVTAHTPLGGSTANIQWFGSVSCLEDPCLQTI
AKKYKKTAAQVSLRWGIQRNTIVIPKSSKVDRVKENMDIFDFELSEEDMKAIAGIDRKL
RTNTPGVFWGIDLFA

>EsRED3-5
MRAKGNSVTQQDHYFTLNTGHRIPAVGLGTWQADGDTCTEAVKTALKVGYRHLDCAHLY
GNELEVGQALKAALNSGVPGLKREDIFVTSKLWCTTNAPKRVETSLRACLKSLGLSYLD
LYLVHWPVSYPFGDATDPPPRKGGAEPKTMRLKATWQAMECLMEKGLVHSIGVSNFSVQ
QIEELLTFAKIVPAVNQVELHPFWRQDDVLKYCQSKGIHVSAHTPLGVPASNTLSDSGF
SGEEECESPRIAFRRCRSVHAPMLKSSVIASIAARLHKTPEQVILRWGMQRGTSVLPRS
LRSERIKTNFDIFDWSLTEDDWKKINSLEPQYRMIDSGYSPSAEARSLQVVQEIDDE

>EsRED4-1
MAQSPMTVLVTGAAGRTGQIVYKKLKERSSEFAARGLVRTEESKQKIGGAEDVFIGDIT
KPETLNEPFQGIDALVILTSAVPKMKPGYDPSKGGRPEFYFEDGCYPEQVDWIGQKNQI
DIAKALGAKQVVVVGSMGGTNPNHPLNSLGNGNILIWKRKAEEYLAGSGVPYTIIRAGG
LLDKEGGMRELLVGKDDELLATDTKSVPRSDVAEVCVQALLHNEAKDKAFDLASKPDEA
GSPTKDFKALFSQVSTKF

>EsNMT1-1
MATLGGASYAMIVKTMMRSLEANLIPDFVLRRLTRILLASRLKLGYKQTAELQLADLMS
FVASLKTMPIALCTEEAKGQHYELPTSFFKLVLGKHLKYSSAYFSEHTRTLDEAEEAML
ALYCERAKIEDGQKILDIGCGWGSFSLYVAERYPKCEITGLCNSSTQKAFIEQQCSERR
LCNVTIYADDISTFDTESTYDRIISIEMFEHMKNYSTLLKKISKWMNQECLLFVHYFCH
KTFAYHFEDVDEDDWMARYFFTGGTMPASSLLLYFQDDVSVVDHWLINGKHYAQTSEEW
LKRMDHNLSSILPIFNETYGENAAKKWLAYWRTFFIAVAELFKYNDGEEWMVSHFLFKK
K

>EsNMT2-1
MAANGFFSDERSVQLSYWKEHTVVPTVEAMMLDSQASTLDQEERPEILSLLPCIEGKSV
IELGAGIGRFTGELAKKADSVLAMDFIDNAIKKNEEINGHHKNVKFVCADVTSPDLGIE
PESADLVFSNWLLMYLSDKEVEDLAQRMLQWVKPGGYIFFRESCFHQSGDHKRKLNPTH
YREPSYYLKVFKESIVKDSSEASYELALVGCKCIAAYVKNKKNQNQICWLWQKVESSDD
KDFQKFLDNSQYTANGILRYERVFGEGYVSTGGIETTKEFVEMMDLKEGEKVLDVGCGI
GGGDFYMAAEYDVEVLAIDLSINMISFALERAIGRKCAVGFEVADCTTKNFPENSFDVI
YSRDTILHIQDKSTLFKSFYKWLKPGGRLLISDYCKAAGTASPEFQEYINQRGYDLHDV
EAYGQMLKNAGFSDVKAEDRSDQFLNILQRELDGMEKQKEAFIKDFSEADYLSIVEGWK
AKIVRSSQGEQKWGLFLAKKQPLC

>EsNMT2-2
MCNRAGSWESGCFTGELAKKAGSVLAVDFINNAIKKNEEINGNHKNVKFVCADVTSPDL
GIEPESADLVFSNWLLMYLSDREVEDLAQRMLQWVKPGGYIFFRESCFHQSGDHKRKVN
PTHYREPSFYLKVFKESIVKDSSKASFELVLVGCKCIVAYVKNKKNQNQICWLWQKVES
SDDKDFQKFLDNYQYTTNGILRYERVFGEGFVSTGGIETTKEFVEMMDLKAGERVLDVG
CGIGGGDFYMAEEYDVEVLGIDLSINMISFALERAIGRKCAVEFEVADCTTKNFPENSF
DVIYSRDTLLHIQDKPTLFRSFYKWLKPGGRVLISDYCKAAGVPSTEFQEYINQRGYDL
HDVESYGQMLKNAGFPDVKAEDRSDQFLNILQRELDDVEKQKDAFIKDFSEADYSSIVD
GWNAKIVRSSQGEQKWGLLLAKKDPFY

>EsNMT3-1
MGVLSMKGGDGEHSYANNSEGQKRLASDAKPVVVETVKEMIVKTDFPGCIKVADLGCSS
GENTLLVMSEIVNTIITSYQQKGKNLPEINCCLNDLPDNDFNTTFKLVPAFHKLLKMDV
KGKCFISGVPGSFYSRLFPSKSLHFVHSSLCLHWLSKVPDGLEDNKKNVYLRSPCPPNV
YKSYLTQFKNDFSLFLRLRADETVPNGRMALTFVGRKSLDPLSKDCFQNWSSISDSLLD
LVSEGIVKESDVDSFNLPFYNPDESEVREVIESEGSFKISNFETIFGLLFSYKTGRTEV
KDDDDNLDQSCRFEVIRKRASIIRSITEPMLGAHFGDAIMDRLFERYTYHLAERYDTLR
NKPTVQFFVSLTRK

>EsNMT3-2
MELQKDKQNQGGQDEIFRMKEGIGDASYYQNSKFQGTAIKLVRKFLEESVHETVRSHGN
QCKTIHIADMGCSSSYNALDASNVIITTTLRALSEDEKPSIPQIFYFFNDLPSNDFNTL
FKIISQTDSQLKNSSVFNAVVSGSFHVRVFPAKSLHFVYSSYALHWLSKVPEEVKDEHS
IAWNPGNVSYDRKTQPSVTSAYLKQFKSDFSSFLKARGEEIVAMGRLFLILGGMDSEDP
QSGGRYEAISKLFNLSFQEMISKGLLSKDIVDTFNIPMFHPSVEEIKEIVNYEGRFEFC
RGKFLKNSEYLLKEDAEGRKRMGKVITKHVQAIFESLVESHFGTTISVRSFEIMNQIVE
EIWEDVVNCWGHGGIVFVELVCK

>EsNMT3-3
MQGGKGETSYAKNSQGQAKHWKSVRPLLEETLKEMALPTGEETVCIADLGCSFGQNTLG
VVGFVVDELQQRYKRSSLVLPEFQAFFSDLPSNDFNTLFHLIDDPDTCDDSNKEKDKDK
PRAYYAAGVPGSFYRRLFPRKSIHVFHSSFSLHWLSQVPDEVQDRSLEAFNKGRVFIHG
GPEAPAEAYLAQFRRDFESFLRARAEETKKGGCMFLVCLGRSTPQLNDQGGGGILFGTH
FESAWSDLVQEGVIEEEERDTFNLPLFAPTAEELLGVVESEGSFRANKVETVRGGSPLV
VKGAGDAEDIAKEYANTCKTVCGVLVEDHIGADKSNQLFKRVGKHAVNNASWLLENLQF
YHVVASLTKK

>EsNMT3-4
MKKPLKEIQSVLHMYVGEGEARYSDNSDRQKYALRVLYPFFEEALFGFKEKFQESNDDS
PLKIADLGCATGPNTLAAFKFVLHSLNDIIYKKNSQPREVQAFFNDLPSNDFNDLFKHL
EKQVEASCYIAGVPGSFYNVLFPKSSIHFCYSVMALHWLSKVPQEVTLEDCDAYNKGSV
WINGGRNEVAEAYARQSQEDLCKFLNCRAHEMVSGGVMFLVLMGRPDSSPPRDQIFTGG
EFCGQDFEEAWNELISLGMITAQQRDAFNLPWYFPNADEITRAANRTGKFDMLSVKVYD
VPSKSEEDFEQYIEDKIKFGRMKSNYVKSFVGSLVEAHLGKDLSDKLFDLFAEKASLLM
EIKPPSRYTVCLVASLLRK

>EsNMT4-1
MWPGEAHSLKVEEVLYQGKSKYQDIMVFQSATYGKVLVLDGVIQVTERDECAYQEMITH
LPLCSIPDPKKVLVIGGGDGGVLREVARHSSVEQIDLCEIDDLVIEVSKKYFPVLSVGF
EDPRVTVHVGDGVAFLKNCPEGTYDAVIVDSSDPIGPAEELFKKPFFELVAKALRPGGV
VSTQAESIWLHMHIIEDIVSVCKQTFKGSVNYAWTTVPTYPSGVIGFMLCSTDGPTVDF
KHPVNPIEKLVQDSSKGPLKFYNSEMHTASFCLPAFARKAIGSQVQQ

>EsNMT4-2
MAEEKKGSANLCCEKARGGQGDVPAAIPGWFSSPPIPTNTNGSKDGANYFNNPMWPGEA
QSLKVDKVLFEGKSEYQELMVFESETYGRVLVLDRVIQLTEKDECAYQEMIAHLPLCSV
PSPKNVLIVGGGDGGVLREVTRHNTVEKVDICEIDKMVIDVSKKFFPELAVGYEDPRVS
LHVGDGAAFLRVCPAGTYDVIIVDSSDPVGPAQELFERPFYETVARALKPGGVAISQAE
SLWLHMHLLQDIISACRQAFRGTVNYAWTSVPTYPSGAIGFVLCTTEGSSIDLKNPVNP
IEKQVDKENLKRPLRFYNSEMHAAAFALPTFVRQALDPLLLHHNSCYRDHKSVHF

>EsNMT5-1
MISSQIVKETKSTESLLGIQPSSRAWLDGISVQQAERMFPFPKAEPSSSPAMQASQQQQ
KMQYTSNVKREREGSSGSNAIEIYDQDRIVTDLRLTVYKSLNRYRDLSLLEPDSASPPS
SALQAEDEMSTRKRKRTIVVHGSAEEAETPTGEERERVRKSLRTFEAIRRHIINEDELL
KDSGGGNRRPDTKAGTVMLDKGLWINRGRRIIGSVPGVHVGDIFFFRLELCVLGLHGPP
QAGIDYVTGKLSGYNEDPIAISIISSGGYKDDEDEGETLIYTGEGGNNYVLDKRQQSFD
QKLERGNLALERSMHYGVEIRVIRGLKDPKSPSGKIYIYDGLYKIEDSWLEKGALGTAV
FKYKLKRIPGQPELGSEIFKKTRTWISFPLCRPSVHHPDLSLRAENYPVCVSNTFGKEI
PFFTYITKVRYPYSLLQSLNLKAKICECSGGCANGPSCSCVETNGGQLPYSSSRVLVKW
KPFIIECNSLCKCPSNCRNRVTQGGLTVHLDVFRTSDRSWGVMSWDAIPAGTFICEYVG
EVLPIEGQSVLSSNERYVFDVHKAQMRWTEEWGNISDLLSEDTQGELVSAFDSLPALNF
IIDARRYGNMSRFIKHSASPNVIVQLVLYDHMDTRYPHVMLFARENIPPLTELTLDYGH
DPPVIL

>EsNMT5-2
MRLFGVLFRKFLQEEEAHSKEPGQRPPRRPDIRASQILKENNRWVNVGKQILGRVPGVE
VGDVFHYRIELCIVGLHRQSQGGIDYLKNGKQIIATSVVAAKGDDVDTGDVLVYKGQGG
FLPGERRGQGEDQKMERGNLSLKNCIDLKSPVRVIRGLKGAEKGGNDSKSKCGITYSYD
GLYLVEKYWDEKGPNGHKVFKFQMRRIPGQPDLPWKIVEDVEKMKSSVKVRVGLRMRDI
SHGKENRPIPVVNTVDDETAPANFEYTSTMMYPSWYDPKPHEGCDCVDGCSDSDKCYCA
VKNGGELPFNYNGAIVEAKPIIYECGPACKCPPTCHNRVSQHGLKIELEVFKTESRGWG
VRALSTIPSGSFVCEYTGELMSDAEAEKRTGNDEYLFDIGSTNDQSLWDGLSDLVNEQP
SNAITEVVEDIGYTIDAYKYGGVGRFINHSCSPNLYAQNVLYDHDDKTLPHIMLFAAEN

IPPLQELSYHYNYKLGQVRDANGIIKKKECYCGSEECEGRLY

>EsNMT5-3
MTIEEVYAARKEAMNSAKHCIINNHQKIQHPTAQRYVHKQRTPLTLNWGSKPATTSRVN
SKPRFQIHLKLNKVAKKPKLALRTNRFESRLCSQFNHGSEEVSARAKVRKVLSLFQFLC
RIFVQNDESPSGTRKRVDFKAAKILRENRRDANPGKQIIGLVPGVEVGDQFRYRMELCL
VGLHRQVQGGIDFIKQKGKSLATSIIVSGGYEDDLDDGEVLVYTGQGGNNYVGDKRQVT
DQKLERGNLALVNSMNKKSPVRVIRGYKNNPSEKNKGGTLYTYDGLYDVERSWDEKGKS
GYIVFKYQLRRHTGQPVLHLGTVQFLGRQKKEAEFKGIEIADISEGKERRCIKAVNEVD
DENGPPKFIYRTKMIYPVTPLPHSGCSCTNGCFDRTKCACVRKNGGEFPYNPKGELVWV
KPLVYECGPDCSCPASCQNRVTQHGVRLNLEVFKTDNKGWGVRSRSPTASGTFVCEYTG
TILNEDQVEECIGSDQYLFNIAGNSNTQTYWTDSLSMLVPNSKYGTDVGVVEDAGFTID
ALKCGNVGRFINHSCSPNLYSVNVLYDHDDKRLPHIMFFAVENIPPMRELSYSYNYPID
QVCDSKGKIKQKECHCGSSECRGMMY

>EsNMT6-1
MAENSRDYESFSGLSVTVVQPNDKYEDVVANFGTDDDNGKFVDFKISNQLDIRAYLNHS
QIYRVGPTLLTLITKTGSFHAKKNLYRSITIGFPTETTCSSFCKTVDDLQGDRLDAKQG
NELVNGDTDLCMKASKFDEKIEASSAKMYFHYYGQLLHQQNMLQDYVRTGTYYAAVVEN
RIDFQGRVVVDVGAGSGILSLFAAQAGARHVYAVEASEMVEYARKLISGNPALGQRITV
IKGKVEEVELPEKADILISEPMGTLLVNERMLESYVIARDRLLHPGGKMFPSVGRIHMA
PFSDEYLFVEVANKALFWQQQNFYGVDLTPLHGSAFQGYFSQPVVDAFDPRVLVAPAIQ
HTLDFTTIKEEELYEIDIPLNFIASVGCRIHGLACWFDVLFNGTAVPRFLTTAPGAPTT
HWYQLRCVLSQPIYVMAGQAITGRLHLVAHSSQSYTMYLTMSAKMWGPGSDQGVIQQTS
SNKLDLKEPYYRMSQPQVYSWPQEQSNQQSTQQQVNGGSFSQLDGISVQQSLPQPDVMQ
QPPSENFQQHSSILQSQEEARMIS

>EsNMT6-2
MLSKRFATLGYHFLPTSACLMHANGKTNNNSNSSGGFRIKKKKMTRHNNSKSSIATLDS
QVTKMSVDDDAQDQLYTGTDRTSADYYFDSYSHFGIHEEMLKDSVRTKTYQRVIYQNSF
LFKDKVVLDVGAGTGILSLFCAKGGAKQVYAIECSEMADMAKEIVKSNGYENVITVIKG
KVEEIELPVEKVDIIISEWMGYFLLFENMLNTVIYARDKWLNTDGIVLPDKASLYLTAI
EDADYKQEKIDYWDNVYGFDMRCIKKQAMVEPLVDTVEPSQIVTNSQLLQSMDISKINS
GEESFTATFSLVAQRNDFIHALVAYFDVAFTKCHKVTSFSTGPRSRATHWKQSVLYLED
VITVCEGEALTGSMTVSPNPKNPRDIDIVLNYAIAGKRCNLSRTQFYRFR

>EsNMT6-3
MSGPSNFSQPPASRVVDKGADFANYFCTYGYLYHQKEMLSDRVRMDAYYSAIFNNKHHF
QDKTVLDVGTGSGILAIWCAQAGARKVYAVEATKMSEHARKLAENNGVGHIVEVIEGNM
EDINLPEKVDVIISEWMGYFLLRESMFDSVIYARDHFLKPNGLMYPSHSRMWLAPIRSG
QGEIKMSDYEAAMSDWHNFVEDTNDFYGVDMSNLSNSYSEEQKRYYLQTSLWNNLHPNQ
IIGNPVVIKEFDCHTATVEEIATLYAKYETSFFIDQGRLNGFAGWFDVHFKGNASNPAC
DDIELSTAPSVENPTHWGQQVFLLNPSERVVNGDVLEGTILMTRSKDNHRLMDVQYAFQ
LKFTSGEISKPITTNYCIE

>EsNMT6-4
MSMHQDFQMGCRENVKRRLRKTLKSDKREFSPPPAFNDHDAAYFGSYSHLGIHEEMIKD
RVRTETYQDAIRYHQDSIRDKVVLDVGCGTGILSIFCALVGARKVYAIDASDIAVQAAE

VVKANNLSDKITVLHGRVEELEIDEEVDVIVSEWMGYMLLYESMLSSVIFARDRWLKRG
GLILPSHATLYLAPVTHPERYSGSIDFWKNVYGIDMSAMIPLAKQCAFEEPCIELISGE
NVLTFPVVIKDIDCSTVTVRDLESITTKFSVVSIMNAPLHGFAIWFNVAFGDPMTSNSV
FSESSFDNRLSGDAYENGMHSRKKLKASPLVLSTAPEEAPTHWAQTVLYIYEPLEVRQD
QMIEGSIRLSQSRENTRFLNIHLEYSSAGRCFVKESILR

>EsNMT6-5
MSAATTEKSHYQTEDEDTDEETNNAWGDWNDDDDNGSISESSSVTCLLCPNSLDSSQQV
FSHCIENHGFDFHKLTSSLRQNFYTTFKIINFLRSQVAENKCWICGSVHGSNDGLLEHL
KLYGHLSIPFEKCPTWQDDRYLKPFLQDDPLLYSLDFEEQDDGKDGFDDIPVVEEECFK
DVGDEALNNMRSLVEDLRIDGEEDSSALEVANGGNCQSKSVENGLSGVLDPKKKSKVSF
AKVAEKEIKNVNENYFGAYSGYTIHREMLGDKVRMDAYGGAILKNPSLFKGAVVMDVGC
GTGILSLFAAQAGAAKVIAVEASQKMASVARQIAKENNLLKEDNGDVGPTNSAGVISVV
EGMMEELDTRMGTAPKTIDVILSEWMGYCLLYESMLSSVIYARDRWLKPGGAILPDTAE
MFVAGFGKGGTSLSFWEDVYGFNMSCIGKEVVEDASQQPIIDVLESKDMVTETSTLQVF
DLMTMKSEEMDFTANFELKLKHESEVCGSGSNKACQENCMQTVCENHVSYEKSIAWCYG
LVLWFETGFTARFCKEMPTILSTSPYNPKTHWSQTILTFKQPIALLADFPNKGLNDSKQ
EIGSASLPASCIKGRISIARSTRHRSIDISVETMATDHYGNVRKLPIQIFNI
