# Supplementary material for: Transcriptome Profiling of Khat (Catha edulis) and Ephedra sinica Reveals Gene Candidates Potentially Involved in Amphetamine-Type Alkaloid Biosynthesis
Source: PLoS One. 2015 Mar 25;10(3):e0119701. doi: 10.1371/journal.pone.0119701 (PMC4373857; doi:10.1371/journal.pone.0119701)
Supplement: S3 Dataset — (PDF) [file pone.0119701.s003.pdf]

Dataset S3. Complete sequences of queries used to mine khat (CED-Trinity) for candidate genes.

>P35510.3_AtPAL1
MEINGAHKSNGGGVDAMLCGGDIKTKNMVINAEDPLNWGAAAEQMKGSHLDEVKRMVAE
FRKPVVNLGGETLTIGQVAAISTIGNSVKVELSETARAGVNASSDWVMESMNKGTDSYG
VTTGFGATSHRRTKNGVALQKELIRFLNAGIFGSTKETSHTLPHSATRAAMLVRINTLL
QGFSGIRFEILEAITSFLNNNITPSLPLRGTITASGDLVPLSYIAGLLTGRPNSKATGP
NGEALTAEEAFKLAGISSGFFDLQPKEGLALVNGTAVGSGMASMVLFETNVLSVLAEIL
SAVFAEVMSGKPEFTDHLTHRLKHHPGQIEAAAIMEHILDGSSYMKLAQKLHEMDPLQK
PKQDRYALRTSPQWLGPQIEVIRYATKSIEREINSVNDNPLIDVSRNKAIHGGNFQGTP
IGVSMDNTRLAIAAIGKLMFAQFSELVNDFYNNGLPSNLTASRNPSLDYGFKGAEIAMA
SYCSELQYLANPVTSHVQSAEQHNQDVNSLGLISSRKTSEAVDILKLMSTTFLVAICQA
VDLRHLEENLRQTVKNTVSQVAKKVLTTGVNGELHPSRFCEKDLLKVVDREQVYTYADD
PCSATYPLIQKLRQVIVDHALINGESEKNAVTSIFHKIGAFEEELKAVLPKEVEAARAA
YDNGTSAIPNRIKECRSYPLYRFVREELGTELLTGEKVTSPGEEFDKVFTAICEGKIID
PMMECLNEWNGAPIPIC

>Q42524.1_At4CL1
MAPQEQAVSQVMEKQSNNNNSDVIFRSKLPDIYIPNHLSLHDYIFQNISEFATKPCLIN
GPTGHVYTYSDVHVISRQIAANFHKLGVNQNDVVMLLLPNCPEFVLSFLAASFRGATAT
AANPFFTPAEIAKQAKASNTKLIITEARYVDKIKPLQNDDGVVIVCIDDNESVPIPEGC
LRFTELTQSTTEASEVIDSVEISPDDVVALPYSSGTTGLPKGVMLTHKGLVTSVAQQVD
GENPNLYFHSDDVILCVLPMFHIYALNSIMLCGLRVGAAILIMPKFEINLLLELIQRCK
VTVAPMVPPIVLAIAKSSETEKYDLSSIRVVKSGAAPLGKELEDAVNAKFPNAKLGQGY
GMTEAGPVLAMSLGFAKEPFPVKSGACGTVVRNAEMKIVDPDTGDSLSRNQPGEICIRG
HQIMKGYLNNPAATAETIDKDGWLHTGDIGLIDDDDELFIVDRLKELIKYKGFQVAPAE
LEALLIGHPDITDVAVVAMKEEAAGEVPVAFVVKSKDSELSEDDVKQFVSKQVVFYKRI
NKVFFTESIPKAPSGKILRKDLRAKLANGL

>NP_563711_AtAAO4
MAGDDLVFAVNGEKFEVLSVNPSTTLLEFLRSNTCFKSVKLSCGEGGCGACIVILSKYD
PVLDQVEEYSINSCLTLLCSLNGCSITTSDGLGNTEKGFHPIHKRFAGFHASQCGFCTP
GMCISLYSALSKAHNSQSSPDYLTALAAEKSIAGNLCRCTGYRPIADACKSFASDVDIE
DLGFNSFWRKGESREEMLKKLPPYNPEKDLITFPDFLKEKIKCQHNVLDQTRYHWSTPG
SVAELQEILATTNPGKDRGLIKLVVGNTGTGYYKEEKQYGRYIDISHIPEMSMIKKDDR
EIEIGAVVTISKVIDALMEENTSAYVFKKIGVHMEKVANHFIRNSGSIGGNLVMAQSKS
FPSDITTLLLAADASVHMINAGRHEKLRMGEYLVSPPILDTKTVLLKVHIPRWIASSTT
GLLFETYRAALRPIGSALPYINAAFLAVVSHDASSSGIIVDKCRLAFGSYGGYHSIRAR
EVEDFLTGKILSHSVLYEAVRLLKGIIVPSIDTSYSEYKKSLAVGFLFDFLYPLIESGS
WDSEGKHIDGHIDPTICLPLLSSAQQVFESKEYHPVGEAIIKFGAEMQASGEAVYVDDI
PSLPHCLHGAFIYSTKPLAWIKSVGFSGNVTPIGVLAVITFKDIPEVGQNIGYITMFGT
GLLFADEVTISAGQIIALVVADTQKHADMAAHLAVVEYDSRNIGTPVLSVEDAVKRSSL
FEVPPEYQPEPVGDISKGMAEADRKIRSVELRLGSQYFFYMETQTALALPDEDNCLVVY
SSTQAPEFTQTVIATCLGIPEHNVRVITRRVGGGFGGKAIKSMPVATACALAAKKMQRP
VRIYVNRKTDMIMAGGRHPLKITYSVGFRSDGKLTALDLNLFIDAGSDVDVSLVMPQNI
MNSLRKYDWGALSFDIKVCKTNLPSRTSLRAPGEVQGSYIAESIIENVASSLKMDVDVV
RRINLHTYESLRKFYKQAAGEPDEYTLPLLWDKLEVSADFRRRAESVKEFNRCNIWRKR
GISRVPIIHLVIHRPTPGKVSILNDGSVAVEVAGIEVGQGLWTKVQQMVAYGLGMIKCE

GSDDLLERIRLLQTDTLSMSQSSYTAGSTTSENCCEAVRLCCGILVERLRPTMNQILEN
ARSVTWDMLIQQANAQSVDLSARTFYKPESSSAEYLNYGVGASEVEVDLVTGRTEIIRS
DIIYDCGKSLNPAVDLGQIEGAFVQGIGFFMYEEYTTNENGLVNEEGTWDYKIPTIDTI
PKQFNVQILNSGHHKNRVLSSKASGEPPLLVAASVHCATRSAIREARKQYLSWNCIDDD
HRERCDLGFELPVPATMPVVKQLCGLESIEKYLEWKTYP

>ACM89738.1_AmBALDH
MAAHRFSSLLSRSVPLLSRGGKQSYLGRGVYRYGTAAAAALEEPIKPPVSVQYDKLLIN
GQFVDAASGKTFPTLDPRSGEVIAHVAEGDAEDINRAVAAARKAFDEGPWPKMPAYERQ
KIMLRFADLVEKHNDEVAALEAWDSGKPYEQCAQVEIPMFVRLFRYYAGWADKIHGLTI
PADGPHHVQTLHEPIGVAGQIIPWNFPLVMFGWKVGPALACGNSVVLKTAEQTPLSALL
VSKLFHEAGLPEGVLNIVSGFGPTAGAALCRHMDVDKLAFTGSTETGKIVLELSAKSNL
KPVTLELGGKSPFIVCEDADVDKAVELAHFALFFNQGQCCCAGSRTFVHEKVYDEFVEK
AKARALKRTVGDPFKAGMEQGPQVDADQFEKILKYIRSGAESGATLETGGDRLGTKGYY
IQPTVFSDVKDDMLIAKDEIFGPVQTILKFKELDEVIRRANNSSYGLAAGVFTQNLDTA
NTMMRALRAGTVWINCFDTFDAAIPFGGYKMSGIGREKGEYSLKNYLQVKAVVTALKNP
AWL

>JX142126.1_PhCHD
MAQVKVTMEVGTDGVAVITIFNPPVNALAIPIINALKEKWTEATIRNDVKAIVLTGNGG
RFSGGFDINVFQKVHGTGDISQMPDVSVDLVVNTMEDCKKPAVAAIEGLALGGGLELAM
GCHARIAAPRAQLGLPELSLGVMPGFGGTQRLPRLIGLSKAVEMMMTSKPIMSEEGKKL
GLIDAIVPSSELLKVSRQWALDIAERRKPWMRSLHKTDKIGSLSEAREVLKVARQQVKQ
TAKNMPQHVACIDVIEEGIIHGGYAGVLKEAKVFKDLVLSETSKGLVHVFFAQRATSKV
PNVTDIGLKPRTVKKVAVIGGGLMGSGIATALALSNTFVVLKEINSEYLQKGMKAIEAN
VRGLVARKKLPQDKADKALSMVKGALDYSDFKDVDMVIEAVIESVPLKQKIFSEIEKVC
PPHCILASNTSTIDLNIIGENTRSKDRIIGAHFFSPAHIMPLLEIVRTEKTSTQAILDL
MAVGKAIKKVPVVVGNCTGFAVNRTFFPYSQGAHILVNLGVDAYRIDAQITSFGLPMGP
LQLQDLTGYGVAVAVGKEFGSAFSDRTFKSPLIDLLIKSGRNGKNNGKGFYIYEKGSKP
RPDLSVLPIIEESRRLTNIMPGGKPISVTDQEIVEMILFPVVNEACRVLDEGIVVRASD
LDVASVLGMSFPSYRGGIVFWADTVGAGHIYKSLTKWSELYGNFFKPSRFLEERATKGI
ALSAPATASSASRSRM

>ACV70032.1_PhKAT1
MEKAIQRQRVLLEHLQPIRHHTHDHSSSLTTSICAAGDSAAYQRTAAFGDDVVIVAAYR
TAICKSKRGGFKDTLSDDLLAPVLKAVIEKTNLDPKEVGDIVVGTVLAPGSIRAMECRM
AAFYAGFPETVPIRTVNRQCSSGLQAVADVAASIKAGFYDIGIGAGLELMTVDNIGRVQ
QRNTKVDTFAQARDCLLPMGITSENVAQRFGVTRLEQDQAAVNSHQRAAAATASGKFKD
EIIPVLTKIVDPQTGKEKPVVISVDDGIRPNTNLTSLGKLKPAFKNDGTTTAGNASQVS
DGAAAVLLMKRSVAMKKGLPILGVFRSFAAVGVDPAVMGIGPAVAIPPAVKSAGLDLDD
IDLYEINEAFASQFVYCQKKLNLDPEKVNVNGGAMALGHPLGATGARCVATLLHEMKRR
GKDCRFGVISMCIGSGMGAAAVFERGDAVDDLCNARVSNNNSFLSKDAK

>NP_176763.1_AtBZO1
MDDLALCEANNVPLTPMTFLKRASECYPNRTSIIYGKTRFTWPQTYDRCCRLAASLISL
NISKNDVVSVMAPNTPALYEMHFAVPMAGAVLNPINTRLDATSIAAILRHAKPKILFLD
RSFEALARESLHLLSSEDSNLNLPVIFIHENDFPKRASFEELDYECLIQRGEPTPSMVA
RMFRIQDEHDPISLNYTSGTTADPKGVVISHRGAYLCTLSAIIGWEMGTCPVYLWTLPM

FHCNGWTFTWGTAARGGTSVCMRHVTAPEIYKNIEMHNVTHMCCVPTVFNILLKGNSLD
LSPRSGPVHVLTGGSPPPAALVKKVQRLGFQVMHAYGQTEATGPILFCEWQDEWNRLPE
NQQMELKARQGISILGLADVDVKNKETQKSAPRDGKTMGEILIKGSSIMKGYLKNPKAT
FEAFKHGWLNTGDVGVIHPDGHVEIKDRSKDIIISGGENISSVEVENVLYKYPKVLETA
VVAMPHPTWGETPCAFVVLEKSETTIKEDRVDKFQTRERNLIEYCRENLPHFMCPRKVV
FLEELPKNGNGKILKPKLRDIAKGLVVEDEINVIAKEVKRPVGHFISRL

>ABJ80681.1_AtAHAS
MAAATTTTTTSSSISFSTKPSPSSSKSPLPISRFSLPFSLNPNKSSSSSRRRGIKSSSP
SSISAVLNTTTNVTTTPSPTKPTKPETFISRFAPDQPRKGADILAEALERQGVETVFAY
PGGASMEIHQALTRSSSIRNVLPRHEQGGVFAAEGYARSSGKSGICIATSGPGATNLVS
GLADALLDSVPLVAITGQVPRRMIGTDAFQGTPIVEVTRSITKHNYLVMDVEDIPRIIE
EAFFLATSGRPGPVLVDVPKDIQQQLAIPNWEQAMRLPGYMSRMPKPPEDSHLEQIVRL
ISESKKPVLYVGGGCLNSSDELGRFVELTGIPVASTLMGLGSYPCDDELSLHMLGMHGT
VYANYAVEHSDLLLAFGVRFDDRVTGKLEAFASRAKIVHIDIDSAEIGKNKTPHVSVCG
DVKLALQGMNKVLENRAEELKLDFGVWRNELNVQKQKFPLSFKTFGEAIPPQHAIKVLD
ELTDGKAIISTGVGQHQMWAAQFYNYKKPRQWLSSGGLGAMGFGLPAAIGASVANPDAI
VVDIDGDGSFIMNVQELATIRVENLPVKVLLLNNQHLGMVMQWEDRFYKANRAHTFLGD
PAQEDEIFPNMLLFAAACGIPAARVTKKADLREAIQTMLDTPGPYLLDVICPHQEHVLP
MIPSGGTFNDVITEGDGRIKY

>NP_200307.1_AtPDC2
MDTKIGSIDACNPTNHDIGGPPNGGVSTVQNTSPLHSTTVSPCDATLGRYLARRLVEIG
VTDVFSVPGDFNLTLLDHLIAEPNLKLIGCCNELNAGYAADGYARSRGVGACVVTFTVG
GLSVLNAIAGAYSENLPLICIVGGPNSNDYGTNRILHHTIGLPDFTQELRCFQAVTCFQ
AVINNLEEAHELIDTAISTALKESKPVYISISCNLPAIPLPTFSRHPVPFMLPMKVSNQ
IGLDAAVEAAAEFLNKAVKPVLVGGPKMRVAKAADAFVELADASGYGLAVMPSAKGQVP
EHHKHFIGTYWGAVSTAFCAEIVESADAYLFAGPIFNDYSSVGYSLLLKKEKAIIVQPD
RVTIGNGPAFGCVLMKDFLSELAKRIKHNNTSYENYHRIYVPEGKPLRDNPNESLRVNV
LFQHIQNMLSSESAVLAETGDSWFNCQKLKLPEGCGYEFQMQYGSIGWSVGATLGYAQA
MPNRRVIACIGDGSFQVTAQDVSTMIRCGQKTIIFLINNGGYTIEVEIHDGPYNVIKNW
NYTAFVEAIHNGEGKCWTAKVRCEEELVKAINTATNEEKESFCFIEVIVHKDDTSKELL
EWGSRVSAANSRPPNPQ

>E9L7A5.1_PhPPA-AT
MAATTTTSSSSRIAYSRHNIPGLHSDSLNPKSISFSSNLHTFSLKSSGSRRQLYSRRTG
AVVIMQSMDKVEVDISLSPRVNSVKPSKTVAITDQATALVQAGVPVIRLAAGEPDFDTP
APIVEAGINAIREGHTRYTPNAGTMELRSAISHKLKEENGLSYTPDQILVSNGAKQSII
QAVLAVCSPGDEVLIPAPYWVSYPEMARLADATPVILPTSISEDFLLDPKLLESKLTEK
SRLLILCSPSNPTGSVYPRKLLEQIAEIVARHPRLLVISDEIYEHIIYAPATHTSFASL
PGMWDRTLTVNGFSKAFAMTGWRLGYIAGPKHFIAACNKIQSQFTSGASSISQKAAVAA
LGLGYAGGELVATMVKSFRERRDYLVKSFGEIEGVKISEPRGAFYLFIDLSSYYGVEVD
GFGSINNSESLCRYLLDKAQVALVPGDAFGDDTCIRISYAASLSTLQAAVERIKKALVT
IKPPVPV

>ADC45389.1_CmArAT1
MEIGAVNSEMDTASTISIKGILSLLVQNADENNGRRLISLGMGDPSAYSCFHTTRIAQD
AVVDCLESEKFNGYAPTVGLPQSRRAIAEYLSRDLPYKLTSDDVFITSGCTQAIDVALA

```
MLARPGANILLPRPGFPIYELCSSFQNLEVRHFNLLPQQGWEVDLHAIETLADKNTVAL
VIINPGNPCGNVYSYQHLKKIAETAEKLGILVIADEVYGHLAFGSRPFVPMGVFGSTVP
VLTLGSLSKRWIVPGWRLGWFVTSDPSGMFRKPKVIERIKKYFDTLGGPATFIQAAVPR
ILESTDEVFFKKTINILKQTSEICCRKIKEIPCITCTHRPEGSMAMMVRLNIDLLEDIS
DDIDFCFKLAKEESLVILPGTAVGLKNWLRITFAVDPSFLEEALGRLKSFCQRHTLML

>AAA33281.1_DsTRI
MEESKVSMMNCNNEGRWSLKGTTALVTGGSKGIGYAIVEELAGLGARVYTCSRNEKELD
ECLEIWREKGLNVEGSVCDLLSRTERDKLMQTVAHVFDGKLNILVNNAGVVIHKEAKDF
TEKDYNIIMGTNFEAAYHLSQIAYPLLKASQNGNVIFLSSIAGFSALPSVSLYSASKGA
INQMTKSLACEWAKDNIRVNSVAPGVILTPLVETAIKKNPHQKEEIDNFIVKTPMGRAG
KPQEVSALIAFLCFPAASYITGQIIWADGGFTANGGF

>AAF13739.1_PsCOR1
MESNGVPMITLSSGIRMPALGMGTAETMVKGTEREKLAFLKAIEVGYRHFDTAAAYQSE
ECLGEAIAEALQLGLIKSRDELFITSKLWCADAHADLVLPALQNSLRNLKLEYLDLYLI
HHPVSLKPGKFVNEIPKDHILPMDYKSVWAAMEECQTLGFTRAIGVSNFSCKKLQELMA
AAKIPPVVNQVEMSPTLHQKNLREYCKANNIMITAHSVLGAIGAPWGSNAVMDSKVLHQ
IAVARGKSVAQVSMRWVYQQGASLVVKSFNEGRMKENLKIFDWELTAEDMEKISEIPQS
RTSSAAFLLSPTGPFKTEEEFWDEKD

>ADE41047_EcSanR
MADSSKKLTVLLSGASGLTGSLAFKKLKERSDKFEVRGLVRSEASKQKLGGGDEIFIGD
ISDPKTLEPAMEGIDALIILTSAIPRMKPTEEFTAEMISGGRSEDVIDASFSGPMPEFY
YDEGQYPEQVDWIGQKNQIDTAKKMGVKHIVLVGSMGGCDPDHFLNHMGNGNILIWKRK
AEQYLADSGVPYTIIRAGGLDNKAGGVRELLVAKDDVLLPTENGFIARADVAEACVQAL
EIEEVKNKAFDLGSKPEGVGEATKDFKALFSQVTTPF
```
