# Supplementary material for: Transcriptome Profiling of Khat (Catha edulis) and Ephedra sinica Reveals Gene Candidates Potentially Involved in Amphetamine-Type Alkaloid Biosynthesis
Source: PLoS One. 2015 Mar 25;10(3):e0119701. doi: 10.1371/journal.pone.0119701 (PMC4373857; doi:10.1371/journal.pone.0119701)
Supplement: S4 Dataset — (PDF) [file pone.0119701.s004.pdf]

Dataset S4. Complete sequences of queries used to mine *Ephedra sinica* (ESI-Velvet) for candidate genes.

>AB300199.1_EsPAL1
MVAGAEMAQTAFVQHVKDGGIREFLCKGSDSSNDPLNWVAAAKSMSGSHFDMVRDMVEV
YLNAKEVSIEGKTLTVAQVTAVARKAEQTAIKLDAEAAKERVEKSANWVLTQMNKGTDT
YGVTTGFGATSHRRTNQGAKLQKELIRFLNAGVLGCDDNVLPVETTRAAMLVRTNTLMQ
GYSGIRWEILAAVENLLNAGLTPKLPLRGTISASGDLVPLSYIAGLLTGRPNCKVSTRD
GTVLSGSEALKQIGIEKPFELQPKEGLAIVNGTAVCAALASLVCFDANVLALLSEVMAA
MFCEVMNGKPEFADPLIHRLKHHPGQMEAAAIMEYVLDGSSYMKHAVEIHERNPLQKPK
QDRYALRTSPQWLGPQIEVIRAATHMIEREINSVSDNPMIDVARDKALHGGNFQGTPIG
VSMDNLRLAVAAIGKLMFAQMSELVNDYYNGGLPSNLSGGPNPSLDYGFKGAEIAMASY
CSELQYLANPVTNHVESAEQHNQDVNSLGMVSARKSEEALEILRLMLSTYLTAICQAID
LRHLEENMLATVKQTVAQVARKTLSTGANGELLPGRFCEKELLQVVENEHVLSYIDDPC
SPNYVLTQKLRGVLVEHALKNREAEKDLRTSIFHRIEEFETELKVQLESQATTVRANFD
NGVTALPNRIKDCRSYPLYSFVRETLGTQLLSGDRDISPGEDIEVVYKAVKANDIIVPL
FKCLDGWKGTPGPF

>AAB42382_Pt4CL
MANGIKKVEHLYRSKLPDIEISDHLPLHSYCFERVAEFADRPCLIDGATDRTYCFSEVE
LISRKVAAGLAKLGLQQGQVVMLLLPNCIEFAFVFMGASVRGAIVTTANPFYKPGEIAK
QAKAAGARIIVTLAAYVEKLADLQSHDVLVITIDDAPKEGCQHISVLTEADETQCPAVK
IHPDDVVALPYSSGTTGLPKGVMLTHKGLVSSVAQQVDGENPNLYFHSDDVILCVLPLF
HIYSLNSVLLCALRAGAATLIMQKFNLTTCLELIQKYKVTVAPIVPPIVLDITKSPIVS
QYDVSSVRIIMSGAAPLGKELEDALRERFPKAIFGQGYGMTEAGPVLAMNLAFAKNPFP
VKSGSCGTVVRNAQIKILDTETGESLPHNQAGEICIRGPEIMKGYINDPESTAATIDEE
GWLHTGDVGYIDDDEEIFIVDRVKEIIKYKGFQVAPAELEALLVAHPSIADAAVVPQKH
EEAGEVPVAFVVKSSEISEQEIKEFVAKQVIFYKKIHRVYFVDAIPKSPSGKILRKDLR
SRLAAK

>NP_563711_AtAAO4
MAGDDLVFAVNGEKFEVLSVNPSTTLLEFLRSNTCFKSVKLSCGEGGCGACIVILSKYD
PVLDQVEEYSINSCLTLLCSLNGCSITTSDGLGNTEKGFHPIHKRFAGFHASQCGFCTP
GMCISLYSALSKAHNSQSSPDYLTALAAEKSIAGNLCRCTGYRPIADACKSFASDVDIE
DLGFNSFWRKGESREEMLKKLPPYNPEKDLITFPDFLKEKIKCQHNVLDQTRYHWSTPG
SVAELQEILATTNPGKDRGLIKLVVGNTGTGYYKEEKQYGRYIDISHIPEMSMIKKDDR
EIEIGAVVTISKVIDALMEENTSAYVFKKIGVHMEKVANHFIRNSGSIGGNLVMAQSKS
FPSDITTLLLAADASVHMINAGRHEKLRMGEYLVSPPILDTKTVLLKVHIPRWIASSTT
GLLFETYRAALRPIGSALPYINAAFLAVVSHDASSSGIIVDKCRLAFGSYGGYHSIRAR
EVEDFLTGKILSHSVLYEAVRLLKGIIVPSIDTSYSEYKKSLAVGFLFDFLYPLIESGS
WDSEGKHIDGHIDPTICLPLLSSAQQVFESKEYHPVGEAIIKFGAEMQASGEAVYVDDI
PSLPHCLHGAFIYSTKPLAWIKSVGFSGNVTPIGVLAVITFKDIPEVGQNIGYITMFGT
GLLFADEVTISAGQIIALVVADTQKHADMAAHLAVVEYDSRNIGTPVLSVEDAVKRSSL
FEVPPEYQPEPVGDISKGMAEADRKIRSVELRLGSQYFFYMETQTALALPDEDNCLVVY
SSTQAPEFTQTVIATCLGIPEHNVRVITRRVGGGFGGKAIKSMPVATACALAAKKMQRP
VRIYVNRKTDMIMAGGRHPLKITYSVGFRSDGKLTALDLNLFIDAGSDVDVSLVMPQNI
MNSLRKYDWGALSFDIKVCKTNLPSRTSLRAPGEVQGSYIAESIIENVASSLKMDVDVV
RRINLHTYESLRKFYKQAAGEPDEYTLPLLWDKLEVSADFRRRAESVKEFNRCNIWRKR
GISRVPIIHLVIHRPTPGKVSILNDGSVAVEVAGIEVGQGLWTKVQQMVAYGLGMIKCE

GSDDLLERIRLLQTDTLSMSQSSYTAGSTTSENCCEAVRLCCGILVERLRPTMNQILEN
ARSVTWDMLIQQANAQSVDLSARTFYKPESSSAEYLNYGVGASEVEVDLVTGRTEIIRS
DIIYDCGKSLNPAVDLGQIEGAFVQGIGFFMYEEYTTNENGLVNEEGTWDYKIPTIDTI
PKQFNVQILNSGHHKNRVLSSKASGEPPLLVAASVHCATRSAIREARKQYLSWNCIDDD
HRERCDLGFELPVPATMPVVKQLCGLESIEKYLEWKTYP

>ACM89738.1_AmBALDH
MAAHRFSSLLSRSVPLLSRGGKQSYLGRGVYRYGTAAAAALEEPIKPPVSVQYDKLLIN
GQFVDAASGKTFPTLDPRSGEVIAHVAEGDAEDINRAVAAARKAFDEGPWPKMPAYERQ
KIMLRFADLVEKHNDEVAALEAWDSGKPYEQCAQVEIPMFVRLFRYYAGWADKIHGLTI
PADGPHHVQTLHEPIGVAGQIIPWNFPLVMFGWKVGPALACGNSVVLKTAEQTPLSALL
VSKLFHEAGLPEGVLNIVSGFGPTAGAALCRHMDVDKLAFTGSTETGKIVLELSAKSNL
KPVTLELGGKSPFIVCEDADVDKAVELAHFALFFNQGQCCCAGSRTFVHEKVYDEFVEK
AKARALKRTVGDPFKAGMEQGPQVDADQFEKILKYIRSGAESGATLETGGDRLGTKGYY
IQPTVFSDVKDDMLIAKDEIFGPVQTILKFKELDEVIRRANNSSYGLAAGVFTQNLDTA
NTMMRALRAGTVWINCFDTFDAAIPFGGYKMSGIGREKGEYSLKNYLQVKAVVTALKNP
AWL

>JX142126.1_PhCHD
MAQVKVTMEVGTDGVAVITIFNPPVNALAIPIINALKEKWTEATIRNDVKAIVLTGNGG
RFSGGFDINVFQKVHGTGDISQMPDVSVDLVVNTMEDCKKPAVAAIEGLALGGGLELAM
GCHARIAAPRAQLGLPELSLGVMPGFGGTQRLPRLIGLSKAVEMMMTSKPIMSEEGKKL
GLIDAIVPSSELLKVSRQWALDIAERRKPWMRSLHKTDKIGSLSEAREVLKVARQQVKQ
TAKNMPQHVACIDVIEEGIIHGGYAGVLKEAKVFKDLVLSETSKGLVHVFFAQRATSKV
PNVTDIGLKPRTVKKVAVIGGGLMGSGIATALALSNTFVVLKEINSEYLQKGMKAIEAN
VRGLVARKKLPQDKADKALSMVKGALDYSDFKDVDMVIEAVIESVPLKQKIFSEIEKVC
PPHCILASNTSTIDLNIIGENTRSKDRIIGAHFFSPAHIMPLLEIVRTEKTSTQAILDL
MAVGKAIKKVPVVVGNCTGFAVNRTFFPYSQGAHILVNLGVDAYRIDAQITSFGLPMGP
LQLQDLTGYGVAVAVGKEFGSAFSDRTFKSPLIDLLIKSGRNGKNNGKGFYIYEKGSKP
RPDLSVLPIIEESRRLTNIMPGGKPISVTDQEIVEMILFPVVNEACRVLDEGIVVRASD
LDVASVLGMSFPSYRGGIVFWADTVGAGHIYKSLTKWSELYGNFFKPSRFLEERATKGI
ALSAPATASSASRSRM

>ACV70032.1_PhKAT1
MEKAIQRQRVLLEHLQPIRHHTHDHSSSLTTSICAAGDSAAYQRTAAFGDDVVIVAAYR
TAICKSKRGGFKDTLSDDLLAPVLKAVIEKTNLDPKEVGDIVVGTVLAPGSIRAMECRM
AAFYAGFPETVPIRTVNRQCSSGLQAVADVAASIKAGFYDIGIGAGLELMTVDNIGRVQ
QRNTKVDTFAQARDCLLPMGITSENVAQRFGVTRLEQDQAAVNSHQRAAAATASGKFKD
EIIPVLTKIVDPQTGKEKPVVISVDDGIRPNTNLTSLGKLKPAFKNDGTTTAGNASQVS
DGAAAVLLMKRSVAMKKGLPILGVFRSFAAVGVDPAVMGIGPAVAIPPAVKSAGLDLDD
IDLYEINEAFASQFVYCQKKLNLDPEKVNVNGGAMALGHPLGATGARCVATLLHEMKRR
GKDCRFGVISMCIGSGMGAAAVFERGDAVDDLCNARVSNNNSFLSKDAK

>NP_176763.1_AtBZO1
MDDLALCEANNVPLTPMTFLKRASECYPNRTSIIYGKTRFTWPQTYDRCCRLAASLISL
NISKNDVVSVMAPNTPALYEMHFAVPMAGAVLNPINTRLDATSIAAILRHAKPKILFLD
RSFEALARESLHLLSSEDSNLNLPVIFIHENDFPKRASFEELDYECLIQRGEPTPSMVA
RMFRIQDEHDPISLNYTSGTTADPKGVVISHRGAYLCTLSAIIGWEMGTCPVYLWTLPM

FHCNGWTFTWGTAARGGTSVCMRHVTAPEIYKNIEMHNVTHMCCVPTVFNILLKGNSLD
LSPRSGPVHVLTGGSPPPAALVKKVQRLGFQVMHAYGQTEATGPILFCEWQDEWNRLPE
NQQMELKARQGISILGLADVDVKNKETQKSAPRDGKTMGEILIKGSSIMKGYLKNPKAT
FEAFKHGWLNTGDVGVIHPDGHVEIKDRSKDIIISGGENISSVEVENVLYKYPKVLETA
VVAMPHPTWGETPCAFVVLEKSETTIKEDRVDKFQTRERNLIEYCRENLPHFMCPRKVV
FLEELPKNGNGKILKPKLRDIAKGLVVEDEINVIAKEVKRPVGHFISRL

>ABJ80681.1_AtAHAS
MAAATTTTTTSSSISFSTKPSPSSSKSPLPISRFSLPFSLNPNKSSSSSRRRGIKSSSP
SSISAVLNTTTNVTTTPSPTKPTKPETFISRFAPDQPRKGADILAEALERQGVETVFAY
PGGASMEIHQALTRSSSIRNVLPRHEQGGVFAAEGYARSSGKSGICIATSGPGATNLVS
GLADALLDSVPLVAITGQVPRRMIGTDAFQGTPIVEVTRSITKHNYLVMDVEDIPRIIE
EAFFLATSGRPGPVLVDVPKDIQQQLAIPNWEQAMRLPGYMSRMPKPPEDSHLEQIVRL
ISESKKPVLYVGGGCLNSSDELGRFVELTGIPVASTLMGLGSYPCDDELSLHMLGMHGT
VYANYAVEHSDLLLAFGVRFDDRVTGKLEAFASRAKIVHIDIDSAEIGKNKTPHVSVCG
DVKLALQGMNKVLENRAEELKLDFGVWRNELNVQKQKFPLSFKTFGEAIPPQHAIKVLD
ELTDGKAIISTGVGQHQMWAAQFYNYKKPRQWLSSGGLGAMGFGLPAAIGASVANPDAI
VVDIDGDGSFIMNVQELATIRVENLPVKVLLLNNQHLGMVMQWEDRFYKANRAHTFLGD
PAQEDEIFPNMLLFAAACGIPAARVTKKADLREAIQTMLDTPGPYLLDVICPHQEHVLP
MIPSGGTFNDVITEGDGRIKY

>NP_200307.1_AtPDC2
MDTKIGSIDACNPTNHDIGGPPNGGVSTVQNTSPLHSTTVSPCDATLGRYLARRLVEIG
VTDVFSVPGDFNLTLLDHLIAEPNLKLIGCCNELNAGYAADGYARSRGVGACVVTFTVG
GLSVLNAIAGAYSENLPLICIVGGPNSNDYGTNRILHHTIGLPDFTQELRCFQAVTCFQ
AVINNLEEAHELIDTAISTALKESKPVYISISCNLPAIPLPTFSRHPVPFMLPMKVSNQ
IGLDAAVEAAAEFLNKAVKPVLVGGPKMRVAKAADAFVELADASGYGLAVMPSAKGQVP
EHHKHFIGTYWGAVSTAFCAEIVESADAYLFAGPIFNDYSSVGYSLLLKKEKAIIVQPD
RVTIGNGPAFGCVLMKDFLSELAKRIKHNNTSYENYHRIYVPEGKPLRDNPNESLRVNV
LFQHIQNMLSSESAVLAETGDSWFNCQKLKLPEGCGYEFQMQYGSIGWSVGATLGYAQA
MPNRRVIACIGDGSFQVTAQDVSTMIRCGQKTIIFLINNGGYTIEVEIHDGPYNVIKNW
NYTAFVEAIHNGEGKCWTAKVRCEEELVKAINTATNEEKESFCFIEVIVHKDDTSKELL
EWGSRVSAANSRPPNPQ

>E9L7A5.1_PhPPA-AT
MAATTTTSSSSRIAYSRHNIPGLHSDSLNPKSISFSSNLHTFSLKSSGSRRQLYSRRTG
AVVIMQSMDKVEVDISLSPRVNSVKPSKTVAITDQATALVQAGVPVIRLAAGEPDFDTP
APIVEAGINAIREGHTRYTPNAGTMELRSAISHKLKEENGLSYTPDQILVSNGAKQSII
QAVLAVCSPGDEVLIPAPYWVSYPEMARLADATPVILPTSISEDFLLDPKLLESKLTEK
SRLLILCSPSNPTGSVYPRKLLEQIAEIVARHPRLLVISDEIYEHIIYAPATHTSFASL
PGMWDRTLTVNGFSKAFAMTGWRLGYIAGPKHFIAACNKIQSQFTSGASSISQKAAVAA
LGLGYAGGELVATMVKSFRERRDYLVKSFGEIEGVKISEPRGAFYLFIDLSSYYGVEVD
GFGSINNSESLCRYLLDKAQVALVPGDAFGDDTCIRISYAASLSTLQAAVERIKKALVT
IKPPVPV

>ADC45389.1_CmArAT1
MEIGAVNSEMDTASTISIKGILSLLVQNADENNGRRLISLGMGDPSAYSCFHTTRIAQD
AVVDCLESEKFNGYAPTVGLPQSRRAIAEYLSRDLPYKLTSDDVFITSGCTQAIDVALA

MLARPGANILLPRPGFPIYELCSSFQNLEVRHFNLLPQQGWEVDLHAIETLADKNTVAL
VIINPGNPCGNVYSYQHLKKIAETAEKLGILVIADEVYGHLAFGSRPFVPMGVFGSTVP
VLTLGSLSKRWIVPGWRLGWFVTSDPSGMFRKPKVIERIKKYFDTLGGPATFIQAAVPR
ILESTDEVFFKKTINILKQTSEICCRKIKEIPCITCTHRPEGSMAMMVRLNIDLLEDIS
DDIDFCFKLAKEESLVILPGTAVGLKNWLRITFAVDPSFLEEALGRLKSFCQRHTLML

>AAA33281.1_DsTRI
MEESKVSMMNCNNEGRWSLKGTTALVTGGSKGIGYAIVEELAGLGARVYTCSRNEKELD
ECLEIWREKGLNVEGSVCDLLSRTERDKLMQTVAHVFDGKLNILVNNAGVVIHKEAKDF
TEKDYNIIMGTNFEAAYHLSQIAYPLLKASQNGNVIFLSSIAGFSALPSVSLYSASKGA
INQMTKSLACEWAKDNIRVNSVAPGVILTPLVETAIKKNPHQKEEIDNFIVKTPMGRAG
KPQEVSALIAFLCFPAASYITGQIIWADGGFTANGGF

>AAA33282_DsTRII
MAGRWNLEGCTALVTGGSRGIGYGIVEELASLGASVYTCSRNQKELNDCLTQWRSKGFK
VEASVCDLSSRSERQELMNTVANHFHGKLNILVNNAGIVIYKEAKDYTVEDYSLIMSIN
FEAAYHLSVLAHPFLKASERGNVVFISSVSGALAVPYEAVYGATKGAMDQLTRCLAFEW
AKDNIRVNGVGPGVIATSLVEMTIQDPEQKENLNKLIDRCALRRMGEPKELAAMVAFLC
FPAASYVTGQIIYVDGGLMANCGF

>AAF13739.1_PsCOR1
MESNGVPMITLSSGIRMPALGMGTAETMVKGTEREKLAFLKAIEVGYRHFDTAAAYQSE
ECLGEAIAEALQLGLIKSRDELFITSKLWCADAHADLVLPALQNSLRNLKLEYLDLYLI
HHPVSLKPGKFVNEIPKDHILPMDYKSVWAAMEECQTLGFTRAIGVSNFSCKKLQELMA
AAKIPPVVNQVEMSPTLHQKNLREYCKANNIMITAHSVLGAIGAPWGSNAVMDSKVLHQ
IAVARGKSVAQVSMRWVYQQGASLVVKSFNEGRMKENLKIFDWELTAEDMEKISEIPQS
RTSSAAFLLSPTGPFKTEEEFWDEKD

>ADE41047_EcSanR
MADSSKKLTVLLSGASGLTGSLAFKKLKERSDKFEVRGLVRSEASKQKLGGGDEIFIGD
ISDPKTLEPAMEGIDALIIILTSAIPRMKPTEEFTAEMISGGRSEDVIDASFSGPMPEFY
YDEGQYPEQVDWIGQKNQIDTAKKMGVKHIVLVGSMGGCDPDHFLNHMGNGNILIWKRK
AEQYLADSGVPYTIIRAGGLDNKAGGVRELLVAKDDVLLPTENGFIARADVAEACVQAL
EIEEVKNKAFDLGSKPEGVGEATKDFKALFSQVTTPF

>AAY79177_PsTNMT
MGSIDEVKKESAGETLGRLLKGEIKDEELKKLIKFQFEKRLQWGYKSSHQEQLSFNLDF
IKSLKKMEMSGEIETMNKETYELPSEFLEAVFGKTVKQSMCYFTHESATIDEAEEAAHE
LYCERAQIKDGQTVLDIGCGQGGLVLYIAQKYKNCHVTGLTNSKAQVNYLLKQAEKLGL
TNVDAILADVTQYESDKTYDRLLMIEAIEHMKNLQLFMKKLSTWMTKESLLFVDHVCHK
TFAHFFEAVDEDDWYSGFIFPPGCATILAANSLLYFQDDVSVVDHWVVNGMHMARSVDI
WRKALDKNMEAAKEILLPGLGGSHETVNGVVTHIRTFCMGGYEQFSMNNGDEWMVAQLL
FKKK

>AAG59894_SlPEANMT
MAAASGQEREVQKSYWIEHTAELTVEAMMLDSKAADLDKEERPEVLSLLPPYEGKTVLE
LGAGIGRFTGELAQKAGQLIALDFIEGAIKKNENINGHYKNVKFMCADVTSPNLIFSPE
SVDLIFSNWLLMYLSDEEVKALVERMVIWLKVGGHIFFRESCFHQSGDHKRKNNPTHYR

DPSFYTKVFRECHVNAGDGKSFELSLAGCKCIGAYVKNKKNQNQICWTWQKVTSKDDME
FQRFLDTVQYKCSGILRYERVFGQGFVSTGGLETTKEFVAMLDLQRGQKVLDVGCGIGG
GNFYMAEKYDVHVVGIDLSINMISFALERAIGLKCAVEFEVADCTKKTYPDCTFDVIYS
RDTILHIQDKPALFRSFYKWLRPGGKVLISDYCKRAGPASKEFEGYIKQRGYDLHDVEA
YGQMLRDAGFHEVVAEDRTEQFIKVLQKELDTVEKERESFIHEFSEQDYNEIVGGWKSK
LIRSSSGEQRWGLFIAKKK

>BAC75663_CaCS
MELQEVLHMNGGEGDTSYAKNSFYNLFLIRVKPILEQCIQELLRANLPNINKCIKVADL
GCASGPNTLLTVRDIVQSIDKVGQEKKNELERPTIQIFLNDLFQNDFNSVFKSLPSFYR
KLEKENGRKIGSCLIGAMPGSFYGRLFPEESMHFLHSCYCLHWLSQVPSGLVTELGISA
NKGCIYSSKASRPPIQKAYLDQFTKDFTTFLRIHSEELISRGRMLLTWICKEDEFENPN
SIDLLEMSINDLVIEGHLEEEKLDSFNVPIYAPSTEEVKCIVEEEGSFEILYLETFKVP
YDAGFSIDDDYQGRSHSPVSCDEHARAAHVASVVRSIFEPIVASHFGEAIMPDLSHRIA
KNAAKVLRSGKGFYDSLIISLAKKPEKSDV

>BAA82264_AbPMT
MEVNHNNGSTTKIILKNGSICNGDVNGNSHTHKKIENKLVECTNSIKPGWFSEFSALWP
GEAFSLKIEKLLFQGKSDYQDVMLFESATYGKVLTLDGAIQHTENGGFPYTEMIVHLPL
GSIPSPKKVLIIGGGIGFTLFEVSRYPTIDTIDIVEIDDVVVDVSRKYFPYLAAGFDDP
RVTLIIGDGAAFVKAAQPGYYDAIIVDSSDPIGPAKDLFERPFFEAVAKALRPGGVVCT
QAESIWLHMHLIKQIIANCRQVFKGSVNYAWTTVPTYPTGVIGYMLCSTEGPEVNFKNP
VNSIDKDTSHVKSKGPLKFYNSDIHKAAFILPSFARDLVEF

>NP_196113_AtSUVH
MERNGGHYTDKTRVLDIKPLRTLRPVFPSGNQAPPFVCAPPFGPFPPGFSSFYPFSSSQ
ANQHTPDLNQAQYPPQHQQPQNPPPVYQQQPPQHASEPSLVTPLRSFRSPDVSNGNAEL
EGSTVKRRIPKKRPISRPENMNFESGINVADRENGNRELVLSVLMRFDALRRRFAQLED
AKEAVSGIIKRPDLKSGSTCMGRGVRTNTKKRPGIVPGVEIGDVFFFRFEMCLVGLHSP
SMAGIDYLVVKGETEEEPIATSIVSSGYYDNDEGNPDVLIYTGQGGNADKDKQSSDQKL
ERGNLALEKSLRRDSAVRVIRGLKEASHNAKIYIYDGLYEIKESWVEKGKSGHNTFKYK
LVRAPGQPPAFASWTAIQKWKTGVPSRQGLILPDMTSGVESIPVSLVNEVDTDNGPAYF
TYSTTVKYSESFKLMQPSFGCDCANLCKPGNLDCHCIRKNGGDFPYTGNGILVSRKPMI
YECSPSCPCSTCKNKVTQMGVKVRLEVFKTANRGWGLRSWDAIRAGSFICIYVGEAKDK
SKVQQTMANDDYTFDTTNVYNPFKWNYEPGLADEDACEEMSEESEIPLPLIISAKNVGN
VARFMNHSCSPNVFWQPVSYENNSQLFVHVAFFAISHIPPMTELTYDYGVSRPSGTQNG
NPLYGKRKCFCGSAYCRGSFG

>NP_199713_AtPRMT
MEIPSLNKQQEFTLASVTDLTSPSSSLSSSPVVATFSCVNEVKELRFQESKSSDGFSFD
LSSTQLFKLGPLQFTCVSDGSISSAKEKSSFSRGVVIKFRDEKDSKEFCDSFEECKKDD
AVKQGSALPNGTVVSANKSKFDDKIEAASAKMYFHYYGQLLHQQNMLQDYVRTGTYHAA
VMENRSDFSGRVVVDVGAGSGILSMFAALAGAKHVYAVEASEMAEYARKLIAGNPLLAE
RITVIKGKIEDIELPEKADVLISEPMGTLLVNERMLETYVIARDRFLSPNGKMFPTVGR
IHMAPFADEFLFVEMANKALFWQQQNYYGVDLTPLYVSAHQGYFSQPVVDAFDPRLLVA
PSMFHVIDFTMMTEEQFYEIDIPLKFTASVCTRIHGLACWFDVLFDGSTVQRWFTTAPG
APTTHWYQIRCVLSQPIHVMAGQEITGRLHLIAHSAQSYTINLTLSAKMWGPGANQGGI
LQTSSCKLDLKEPYYRMSQPQVYPTQEPPAQSQDIHIHSDDLEELELLQQNANAQL
