# Supplementary material for: Transcriptome Profiling of Khat (Catha edulis) and Ephedra sinica Reveals Gene Candidates Potentially Involved in Amphetamine-Type Alkaloid Biosynthesis
Source: PLoS One. 2015 Mar 25;10(3):e0119701. doi: 10.1371/journal.pone.0119701 (PMC4373857; doi:10.1371/journal.pone.0119701)
Supplement: S1 Fig — Data reflect overall length and quantity of RNA molecules prior to preparation for Illumina GA sequencing. (PDF) [file pone.0119701.s005.pdf]

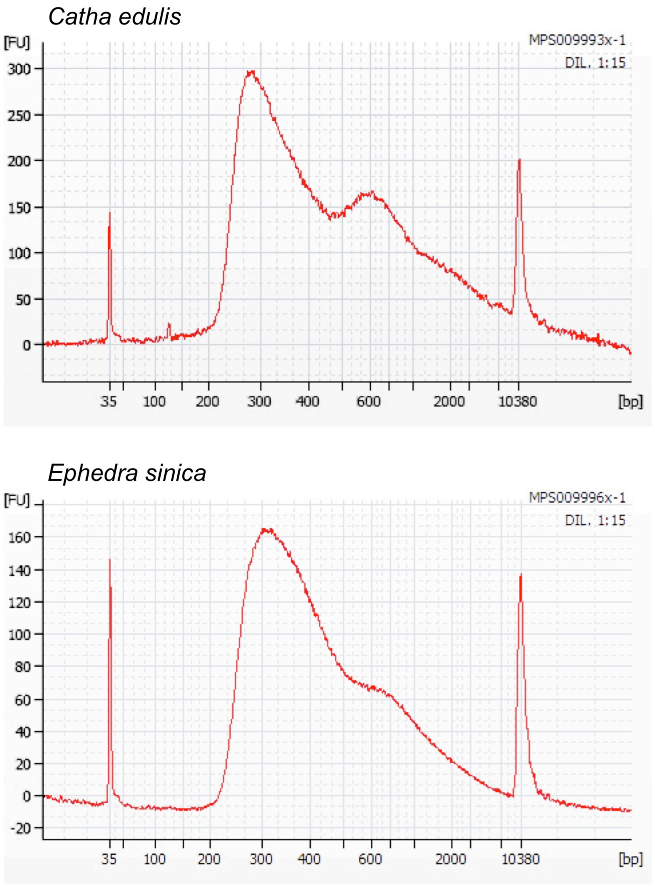

Figure S1. Agilent Bioanalyzer scan results obtained for khat (upper panel) and *Ephedra sinica* (lower panel) total RNA preparations. Data reflect overall length and quantity of RNA molecules prior to preparation for Illumina GA sequencing.
