# Supplementary material for: Transcriptome Profiling of Khat (Catha edulis) and Ephedra sinica Reveals Gene Candidates Potentially Involved in Amphetamine-Type Alkaloid Biosynthesis
Source: PLoS One. 2015 Mar 25;10(3):e0119701. doi: 10.1371/journal.pone.0119701 (PMC4373857; doi:10.1371/journal.pone.0119701)
Supplement: S2 Fig — (PDF) [file pone.0119701.s006.pdf]

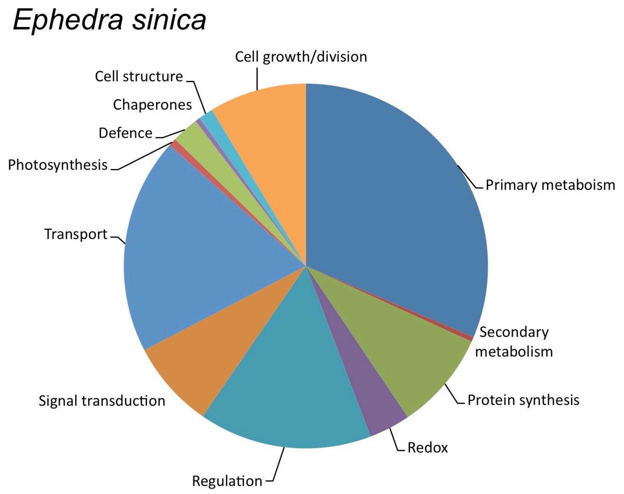

Figure S2. Functional category analysis based on Gene Ontology (GO) annotations of ESI-Velvet unigenes.
