# Supplementary material for: Transcriptome Profiling of Khat (Catha edulis) and Ephedra sinica Reveals Gene Candidates Potentially Involved in Amphetamine-Type Alkaloid Biosynthesis
Source: PLoS One. 2015 Mar 25;10(3):e0119701. doi: 10.1371/journal.pone.0119701 (PMC4373857; doi:10.1371/journal.pone.0119701)
Supplement: S3 Fig — Abbreviations: L-phenylalanine ammonia lyase (PAL) (A), 4-coumaroyl-CoA ligase (4CL) (B), 3-ketoacyl-CoA-thiolase (KAT) (C), cinnamoyl-CoA hydratase-dehydrogenase (CHD) (D), benzoate CoA-ligase (BL) (E), reductase (RED) (F), and N-methyltransferase (NMT) (G). Sequences were aligned and analyzed for phylogenetic relationships using the neighbor-joining algorithm. Numbers at each node represent bootstrap values calculated using 1000 iterations. Accession numbers are found in the Materials and Methods, and abbreviations are defined in Tables 2 and 3. (PDF) [file pone.0119701.s007.pdf]

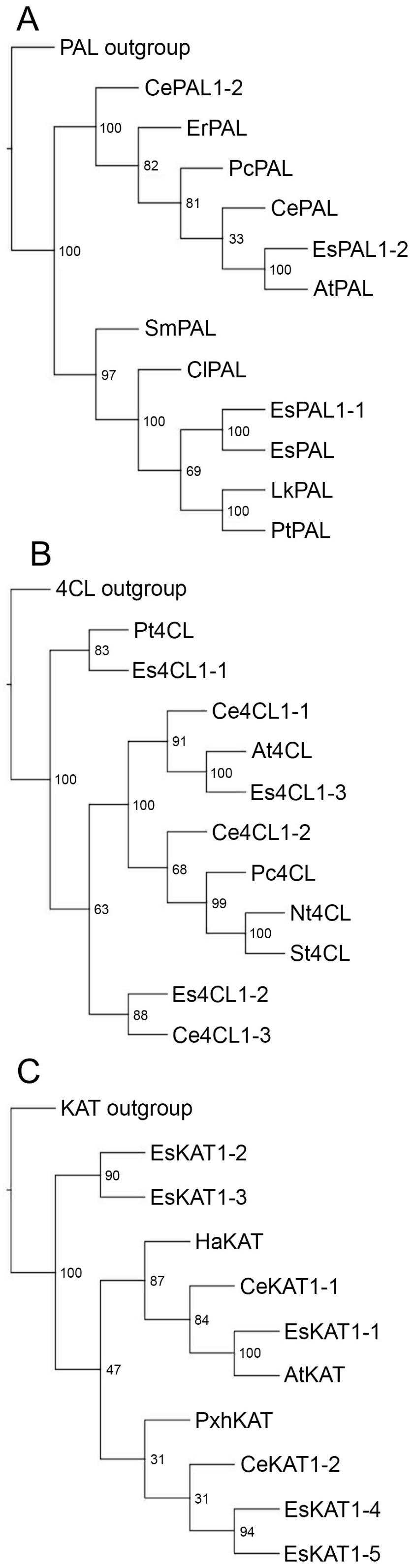

A
PAL outgroup
CePAL1-2
ErPAL
PcPAL
CePAL
EsPAL1-2
AtPAL
SmPAL
ClPAL
EsPAL1-1
EsPAL
LkPAL
PtPAL
100
82
81
33
100
100
97
100
69
100
100
B
4CL outgroup
Pt4CL
Es4CL1-1
Ce4CL1-1
At4CL
Es4CL1-3
Ce4CL1-2
Pc4CL
Nt4CL
St4CL
Es4CL1-2
Ce4CL1-3
83
91
100
100
100
68
99
100
63
88
C
KAT outgroup
EsKAT1-2
EsKAT1-3
HaKAT
CeKAT1-1
EsKAT1-1
AtKAT
PxhKAT
CeKAT1-2
EsKAT1-4
EsKAT1-5
90
100
87
84
100
47
31
31
94

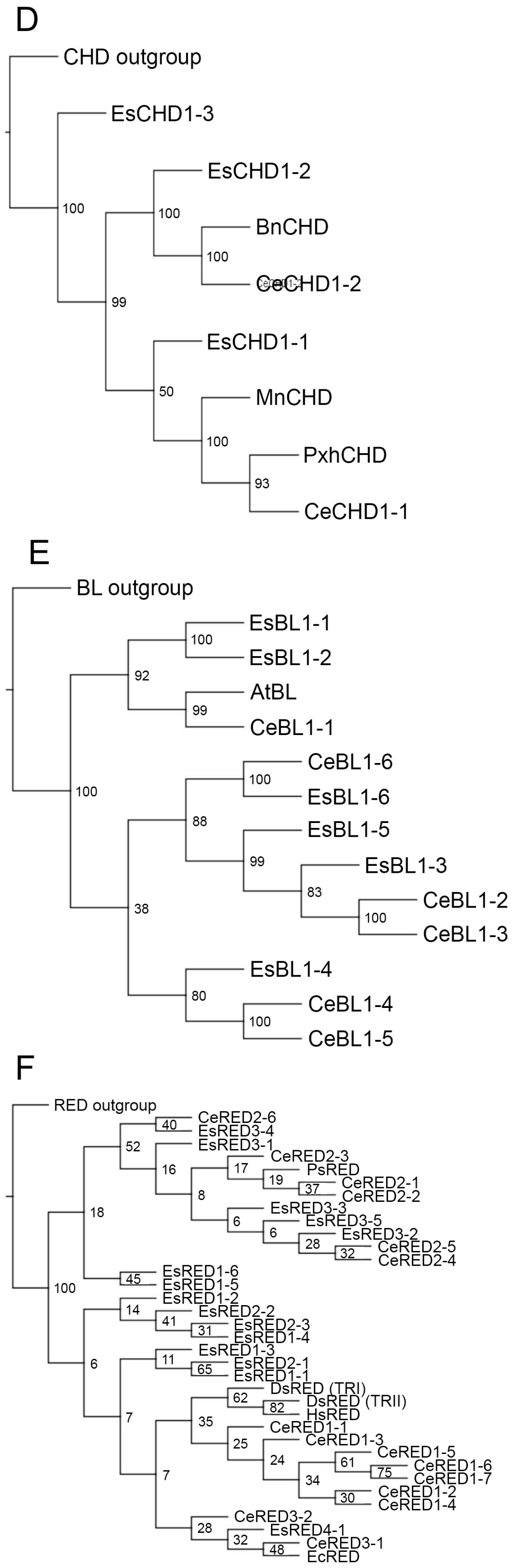
D
CHD outgroup
EsCHD1-3
EsCHD1-2
BnCHD
CeCHD1-2
EsCHD1-1
MnCHD
PxhCHD
CeCHD1-1
E
BL outgroup
EsBL1-1
EsBL1-2
AtBL
CeBL1-1
CeBL1-6
EsBL1-6
EsBL1-5
EsBL1-3
CeBL1-2
CeBL1-3
EsBL1-4
CeBL1-4
CeBL1-5
F
RED outgroup
CeRED2-6
EsRED3-4
EsRED3-1
CeRED2-3
PsRED
CeRED2-1
CeRED2-2
EsRED3-3
EsRED3-5
EsRED3-2
CeRED2-5
CeRED2-4
EsRED1-6
EsRED1-5
EsRED1-2
EsRED2-2
EsRED2-3
EsRED1-4
EsRED1-3
EsRED2-1
EsRED1-1
DsRED (TRI)
DsRED (TRII)
HsRED
CeRED1-1
CeRED1-3
CeRED1-5
CeRED1-6
CeRED1-7
CeRED1-2
CeRED1-4
CeRED3-2
EsRED4-1
CeRED3-1
EcRED

G

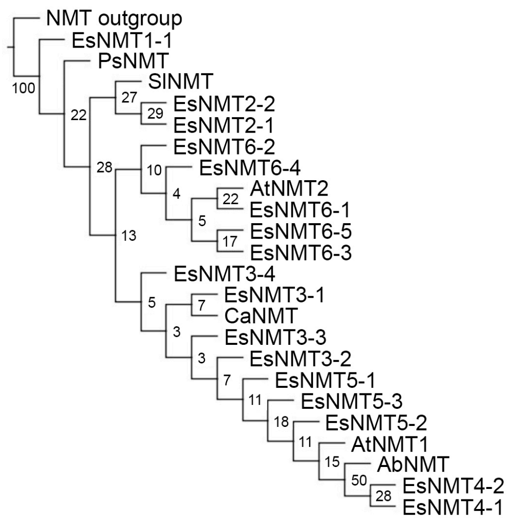

Figure S3. Phylogenetic analysis of gene candidates. Abbreviations: L-phenylalanine ammonia lyase (PAL) (A), 4-coumaroyl-CoA ligase (4CL) (B), 3-ketoacyl-CoA-thiolase (KAT) (C), cinnamoyl-CoA hydratase-dehydrogenase (CHD) (D), benzoate CoA-ligase (BL) (E), reductase (RED) (F), and *N*-methyltransferase (NMT) (G). Sequences were aligned and analyzed for phylogenetic relationships using the neighbor-joining algorithm. Numbers at each node represent bootstrap values calculated using 1000 iterations. Accession numbers are found in the Materials and Methods, and abbreviations are defined in Tables 2 and 3.
